# Gram-scale preparation of negative-type liquid crystals with a CF_2_CF_2_-carbocycle unit via an improved short-step synthetic protocol

**DOI:** 10.3762/bjoc.14.10

**Published:** 2018-01-15

**Authors:** Tatsuya Kumon, Shohei Hashishita, Takumi Kida, Shigeyuki Yamada, Takashi Ishihara, Tsutomu Konno

**Affiliations:** 1 Faculty of Molecular Chemistry and Engineering, Kyoto Institute of Technology, Matsugasaki, Sakyo-ku, Kyoto 606-8585, Japan

**Keywords:** gram-scale preparation, liquid crystals, short-step preparation, tetrafluorinated cyclohexadiene, tetrafluorinated cyclohexane

## Abstract

Herein, we demonstrate an improved short-step protocol for the synthesis of multicyclic molecules having a CF_2_CF_2_-containing cyclohexadiene or cyclohexane framework in a mesogenic structure. These molecules are promising candidates for vertical alignment (VA)-mode liquid crystal (LC) display devices owing to their large negative dielectric constant. The tetrafluorinated multicyclic molecules were successfully obtained in only five or six reaction steps without the need for special handling techniques, as is generally required for thermally unstable organometallic species, representing a reduction of three reaction steps. The improved short-step synthetic protocol was also amenable to the multigram preparation of these promising molecules, which may contribute significantly to the development of novel negative-type LC molecules containing CF_2_CF_2_ carbocycles.

## Introduction

Fluorine-containing organic compounds have attracted much attention in various areas, such as the medicinal, agrochemical, and materials science fields [1–3], due to the unique characteristics of the fluorine atom [4–6]. It is well known that fluorine atoms incorporated into organic substances very often lead to intriguing physical as well as chemical properties. Therefore, considerable attention has been devoted to the development of efficient synthetic protocols for fluorine-containing organic compounds.

**Figure 1:**
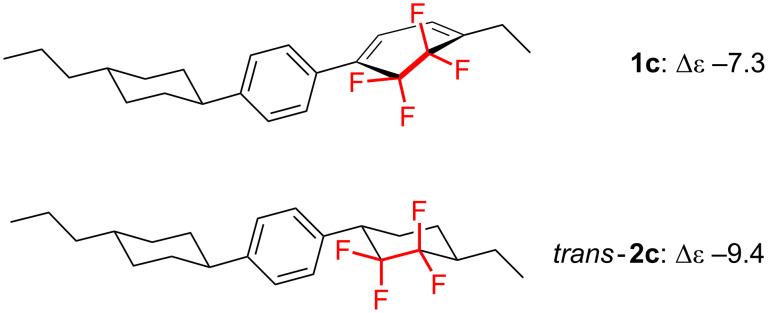
Typical examples of previously reported negative-type liquid crystals containing a CF_2_CF_2_-carbocycle.

Owing to the fascinating molecular properties of organofluorine compounds exerted by the fluorine atom, our research group has devoted sustained effort to the development of novel biologically active fluorinated substances and high-functional fluorinated materials thus far [7–9]. Our recent interest inspired by the discovery of fluorinated liquid-crystalline (LC) molecules [10–11] led to the rational molecular design and synthesis of a family of novel fluorinated LC molecules that possess large negative dielectric anisotropy (Δε). In fact, as shown in Figure 1, tricyclic molecules containing a CF_2_CF_2_ carbocycle, e.g., the 5,5,6,6-tetrafluorocyclohexa-1,3-diene [12] or the 1,1,2,2-tetrafluorocyclohexane motif [13], were successfully synthesized and found to exhibit a large negative Δε value (−7.3 for **1c** and −9.4 for *trans*-**2c**) [14]. This indicated that these compounds are promising candidates for vertical alignment (VA)-type display materials.

In spite of their valuable utility, synthetic procedures for generating the aforementioned CF_2_CF_2_-containing LC molecules inevitably require a multistep protocol, viz. eight steps for **1** and nine steps for **2**, which is a substantial drawback for the practical application of these compounds. Therefore, for practical use of fluorine-containing LC molecules, the development of more efficient synthetic protocols is highly necessary. Herein, an improved short-step synthetic protocol for obtaining promising LC molecules containing the CF_2_CF_2_ fragment is demonstrated, where the improved methodology enables us to prepare the CF_2_CF_2_-containing cyclohexadiene **1a** and the corresponding cyclohexane **2c**, as selected examples, on the multigram scale.

## Results and Discussion

### Improved synthetic design

**Scheme 1:**
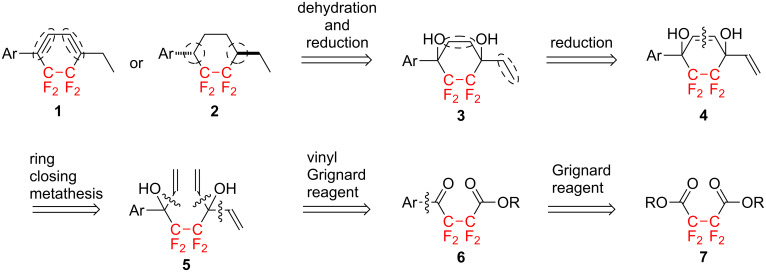
Improved short-step synthetic protocol for multicyclic mesogens **1** and **2**.

In order to establish an improved synthetic protocol, we initially designed a method for the multicyclic mesogens **1** and **2** containing a CF_2_CF_2_ carbocycle which is shown in Scheme 1.

The desired multicyclic molecules **1** with a tetrafluorocyclohexadiene or **2** with a tetrafluorocyclohexane moiety could be prepared starting from the same precursor, e.g., tetrafluorocyclohexane-1,4-diol **3**: through dehydration in the case of **1** or radical reduction through the corresponding bisxanthate derivative in the case of **2**. The required diol **3** could be obtained through a simultaneous hydrogenation of both, the cyclohexene and vinyl moieties of 1-aryl-4-vinyl-5,5,6,6-tetrafluorocyclohex-2-ene-1,4-diol **4**. The latter could be constructed through ring-closing metathesis of the corresponding precursor, e.g., 4,4,5,5-tetrafluoroocta-1,7-diene **5**, using a Grubbs' catalyst. The octa-1,7-diene **5** could be obtained through a nucleophilic addition of a vinylic Grignard reagent to the γ-keto ester **6**. Lastly, the γ-keto ester **6** could be prepared by an addition–elimination reaction of commercially available tetrafluorosuccinic acid diester **7**.

The designed reaction protocol enabled the construction of the target multicyclic molecules in only five or six steps, which is a more efficient protocol with three reaction steps less than the previous method. In addition, the present synthetic protocol involves several standard organic transformations, such as hydrogenation and dehydration, which are advantageous for a large-scale synthesis of the target compounds. Thus, we attempted a detailed examination of the short-step manipulations for obtaining the CF_2_CF_2_-containing multicyclic molecules **1** and **2**.

### Scope and limitation

**Scheme 2:**
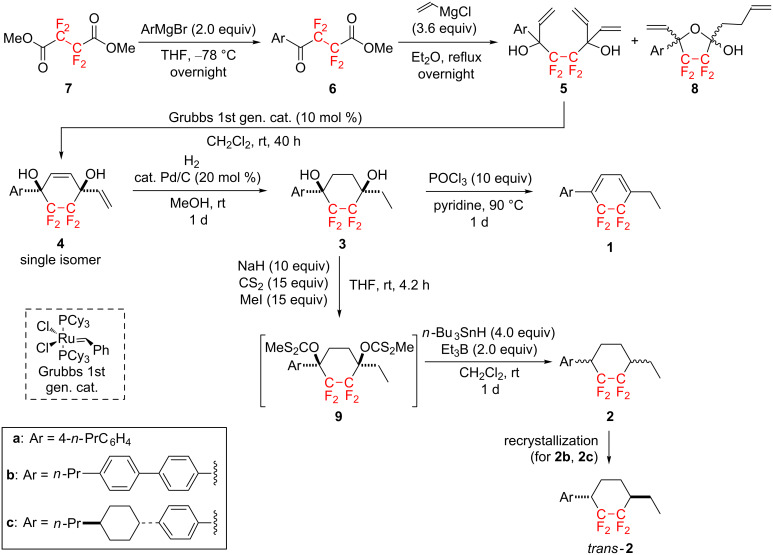
Short-step approach to CF_2_CF_2_-containing carbocycles.

The synthetic route was initiated by the sequential addition–elimination reaction of 4-*n*-propylphenylmagnesium bromide (4-*n*-PrC_6_H_4_MgBr) with commercially available dimethyl tetrafluorosuccinate (**7**, Scheme 2) and the results are summarized in Table 1.

**Table 1:** Yields of all reaction steps in Scheme 2.

	Isolated yield [%]					

	**6**	**5**/**8**	**4**	**3**	**1**	**2** (*trans/cis*)^a^

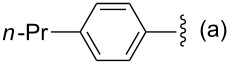	85	46/42	75	99	82	47 (79/21)
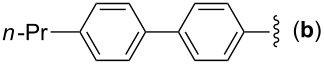	67	43/47	59	96	96	59^b^ (72/28^b^→100/0^c^)^d^
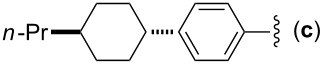	86	38/46	71	100	74	59^b^ (87/13^b^→100/0^c^)^d^

^a^Determined by ^19^F NMR. ^b^Before recrystallization. ^c^After recrystallization. ^d^Previously reported in [13].

**Figure 2:**
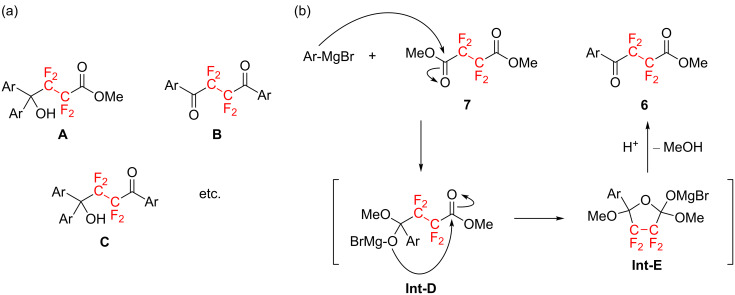
(a) Expected products of over-reaction in the Grignard reaction of dimethyl tetrafluorosuccinate (**7**) with 4-*n*-propylmagnesium bromide (reaction step **7**→**6**). (b) Mechanism for the **7**→**6** reaction step.

Thus, the treatment of 1.0 equiv of **7** with 2.0 equiv of 4-*n*-PrC_6_H_4_MgBr in THF at −78 °C overnight gave the corresponding γ-keto ester **6a** in 85% isolated yield. Interestingly, although using an excess amount of Grignard reagent in this reaction, no adducts by over-reactions, e.g., **A**, **B**, **C**, etc. (Figure 2a), were observed.

The suppression of the formation of the over-reacted products **A**–**C** may be brought about by the following possible reaction pathway (Figure 2b): (i) the nucleophilic attack of the Grignard reagent on the ester carbonyl functionality leads to the formation of the corresponding magnesium acetal **Int-D**, (ii) the alkoxide attacks another ester carbonyl moiety in the molecule to form the corresponding 5-membered ring acetal intermediate (**Int-E**) [15–17], after which immediate hydrolysis leads to the exclusive formation of the corresponding monosubstituted product **6**. The intermediate **Int-E** may be quite stable at −78 °C and in equilibrium with **Int-D** at the stated temperature because of the high electrophilicity of the carbonyl moiety derived from the strong electron-withdrawing effect of the perfluoroalkylene fragment [18–19]. Accordingly, the over-reactions did not occur, and the γ-keto ester **6a** was exclusively obtained after acid treatment of the reaction mixture.

**Scheme 3:**
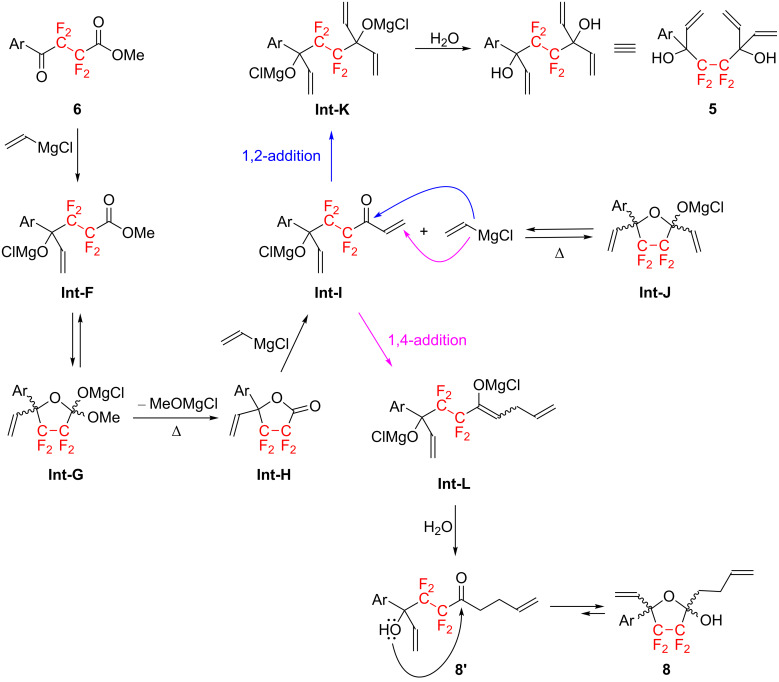
Mechanism for the reaction of γ-keto ester **6** with vinyl Grignard reagents.

As shown in Scheme 2, the isolated γ-keto ester **6a** was treated with a large excess (3.6 equiv) of the vinyl Grignard reagent in diethyl ether at reflux overnight to afford the corresponding octa-1,7-diene **5a** in only 46% yield. In this case, the 5-membered lactol derivative **8a** was also obtained in 42% yield as a side-product. A proposed reaction mechanism for the formation of **5a** and **8a** is shown in Scheme 3.

Thus, the nucleophilic addition reactions of vinylmagnesium chloride with the γ-keto ester **6** furnishes the corresponding magnesium alkoxide **Int-F**, which can be easily converted to the 5-membered ring intermediate, **Int-G**, as already discussed in the reaction of **7**→**6** (Figure 2b). However, the reaction at a higher temperature may lead to the elimination of MeOMgCl, generating the lactone **Int-H**. Accordingly, **Int-H** could undergo a second Grignard reaction with vinylmagnesium chloride present in the reaction mixture, resulting in the formation of **Int-I**. The latter intermediate, **Int-I** which is also in equilibrium with **Int-J** at reflux temperature, can react with the vinylic Grignard reagent through two possible pathways. As indicated by the blue arrow in Scheme 3, when vinylmagnesium chloride attacks the carbonyl carbon of **Int-I**, the corresponding adduct **Int-K** is formed in situ; this adduct can be smoothly converted into the desired octa-1,7-diene **5a** after acid hydrolysis. On the other hand, the β-carbon at the α,β-unsaturated carbonyl moiety of **Int-I** may be susceptible to attack by the vinylic Grignard reagent, as shown by the purple arrow in Scheme 3, because of the high electrophilicity and lack of steric hindrance. As a consequence, **Int-I** also undergoes conjugate addition reaction, a Michael addition reaction, giving rise to the corresponding magnesium enolate **Int-L**. The subsequent hydrolysis of **Int-L** then leads to the γ-hydroxyketone **8a'**, which easily tautomerizes into the more stable 5-membered hemiacetal **8a**.

From the above insight into the reaction mechanism, if the 1,2-addition reaction of **Int-I** proceeds in preference to the conjugate addition reaction, the desired octa-1,7-diene **5a** should be produced in higher yield. However, no significant improvement was observed after various attempts such as employing a more nucleophilic lithium reagent instead of the Grignard reagent [20] or the addition of a Lewis acid to the reaction mixture [21–22]. Fortunately, **5a** and **8a** are easily separable by silica gel column chromatography, and the obtained octa-1,7-diene **5a** was employed in the ensuing reaction without further attempts to improve its yield.

The subsequent ring-closing metathesis [23–25] of **5a** under the influence of less than 10 mol % of a Grubbs 1st generation catalyst did not proceed to completion, resulting in recovery of the starting material along with the desired adduct **4a**. Increasing the reaction temperature did not lead to satisfactory results. However, performing the reaction in the presence of 10 mol % of the catalyst for 40 h drove the reaction to completion. Intriguingly, the ring-closing metathesis proceeded in a highly diastereoselective manner and produced the corresponding cyclized adduct **4a** as a single isomer [26].

Finally, with compound **4a** in hand, the successive hydrogenation in the presence of 20 mol % of Pd/C in methanol was performed for 1 d and generated the corresponding tetrafluorinated cyclohexane-1,4-diol **3a** in quantitative yield. Compound **3a** could be converted to the cyclohexadiene **1a** in 82% isolated yield under the influence of a large amount of phosphorus oxychloride in pyridine at 90 °C for 1 d according to the previous literature [12]. On the other hand, **4a** could also be transformed into the corresponding bisxanthate derivative **9a** according to the literature procedure [13]. Without further purification, **9a** was treated with 4.0 equiv of *n*-Bu_3_SnH and 2.0 equiv of Et_3_B in dichloromethane at room temperature for 1 d to afford the desired reduction products **2a** as an inseparable diastereomeric mixture in a *trans*/*cis* ratio of ca. 80:20 in 47% isolated yield [27].

**Scheme 4:**
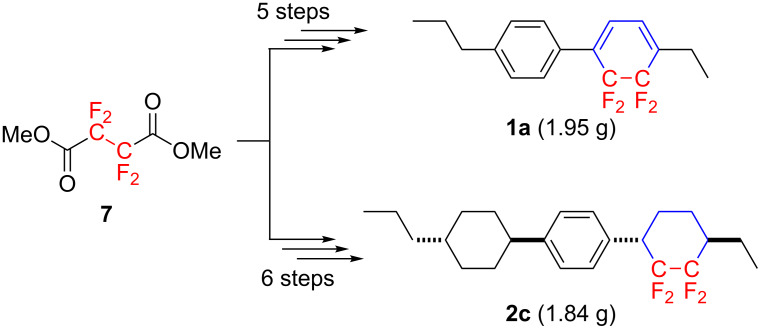
First multigram-scale preparation of CF_2_CF_2_-containing multicyclic mesogens.

With the established short-step protocol, we subsequently synthesized **1b**, **1c**, **2b**, and **2c** using the corresponding Grignard reagents, e.g., (4-*n*-PrC_6_H_4_)C_6_H_4_MgBr or 4-(*trans*-4-*n*-Pr-*c*-C_6_H_10_)C_6_H_4_MgBr, instead of 4-*n*-PrC_6_H_4_MgBr. As shown in Table 1, all reactions proceeded well to afford the corresponding adducts in acceptable to excellent yields. Tricyclic cyclohexanes containing the CF_2_CF_2_ fragment, e.g., **2b** and **2c**, were also obtained as a mixture of *trans*/*cis* diastereomers, but careful recrystallization enabled the isolation of the *trans* isomers in a pure form. A noteworthy advantage of the current synthetic protocol is that starting from the commercially available fluorine-containing substance **7**, multigram-scale preparation of the promising negative-type LC molecules could be firstly achieved and ca. 2.0 g each of tetrafluorocyclohexadiene **1a** and the cyclohexane derivative **2c** (Scheme 4) were obtained. This achievement may lead to a significant contribution to the LC display industry.

The stereochemistry in the ring-closing metathesis step **5**→**4** was determined as follows. As depicted in Table 2, it has been generally recognized that the melting points of *trans*-1,4-disubstituted cyclohexane-1,4-diols are much higher than those of the corresponding *cis*-counterparts [28].

**Table 2:** Melting points of various 1,4-disubstituted cyclohexane-1,4-diol derivatives.

	Melting point (mp) [°C]	
	
R	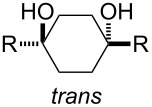	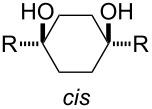

H	143	100–102
CH_3_	199–200	166–167
Ph	234.5–235	150.5–151
HC≡CCH_2_	193	131
CH_2_=CHCH_2_–	133	72

**Scheme 5:**
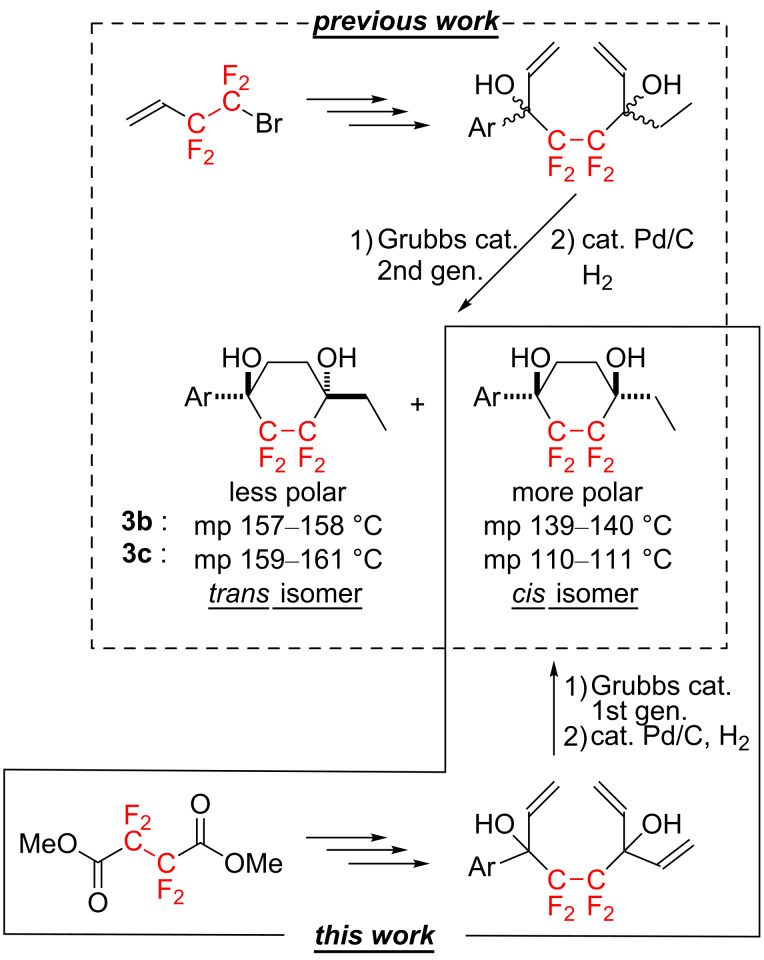
Stereochemical assignment of the ring-closing metathesis products.

As shown in Scheme 5, diastereomeric mixtures of *trans*- and *cis*-1-ethyl-2,2,3,3-tetrafluoro-4-[4-(4-*n*-propylphenyl)phenyl]cyclohexane-1,4-diol (**3b**) or 1-ethyl-2,2,3,3-tetrafluoro-4-[4-(*trans*-4-*n*-propylcyclohexyl)phenyl]cyclohexane-1,4-diol (**3c**) were obtained through an alternative procedure, starting from commercially available 4-bromo-3,3,4,4-tetrafluorobut-1-ene [12–13]. Fortunately, the *trans/cis* diastereomers **3b** and **3c** could be separated from each other through silica gel column chromatography. The careful thermal analyses of the diastereomers **3b** and **3c** [12] revealed that the melting points of the less polar products were approximately 20–50 °C higher than those of the more polar products, indicating that the former and the latter could be successfully assigned as the *trans*- and *cis*-isomer, respectively. On the other hand, the products **3b** and **3c** obtained in the present study were found to be identical to the more polar adducts based on comparison of various physical data, such as the ^1^H, ^13^C, ^19^F nuclear magnetic resonance (NMR) signals, retardation factors (*R*_f_ values), melting points, etc. From these analyses, **3b** and **3c** obtained by the present protocol were eventually determined to be the *cis*-isomer in both cases.

## Conclusion

In summary, we demonstrated the efficient short-step, gram-scale preparation of tetrafluorinated cyclohexadiene or cyclohexane derivatives, which are very promising negative-type LC molecules, starting from commercially available dimethyl 2,2,3,3-tetrafluorosuccinate (**7**). A total of only five or six steps were required for the synthesis of the cyclohexadiene or cyclohexane derivatives, respectively. It should also be noted that the present reaction pathways did not require specific techniques or sensitive reagents, and gram-scale preparation was successfully achieved. The present synthetic protocol is promising for the development of a wide range of negative-type LC molecules containing CF_2_CF_2_ carbocycles by the selection of the starting Grignard reagent and should contribute to further evolution of VA-type LC display molecules.

## Supporting Information

File 1 Experimental procedures, characterization data, and copies of ^1^H, ^13^C and ^19^F NMR spectra.
