# Supplementary material for: Gram-scale preparation of negative-type liquid crystals with a CF2CF2-carbocycle unit via an improved short-step synthetic protocol
Source: Beilstein J Org Chem. 2018 Jan 15;14:148–54. doi: 10.3762/bjoc.14.10 (PMC5789429; doi:10.3762/bjoc.14.10)

Supporting Information

for

Gram-scale preparation of negative-type liquid crystals with a CF_2CF_2-carbocycle unit via an improved short-step synthetic protocol

Tatsuya Kumon, Shohei Hashishita, Takumi Kida, Shigeyuki Yamada, Takashi Ishihara and Tsutomu Konno*

Address: Faculty of Molecular Chemistry and Engineering, Kyoto Institute of Technology, Matsugasaki, Sakyo-ku, Kyoto 606-8585, Japan

Email: Tsutomu Konno - konno@kit.ac.jp

*Corresponding author

Experimental procedures, characterization data, and copies of ^{1}H, ^{13}C and ^{19}F NMR spectra

Experimental

General procedures

Infrared spectra (IR) were determined as liquid films on a NaCl plate or by the KBr disk method with a JASCO FT/IR-4100 type A spectrometer and all spectra are reported in wavenumbers (cm^{-1}). ^{1}H and ^{13}C NMR spectra were measured with a JEOL JNM-AL 400 NMR spectrometer (^{1}H: 400 MHz and ^{13}C: 100 MHz) in a chloroform-*d* ($CDCl_3$) solution and the chemical shifts are reported in parts per million (ppm) using the residual proton or carbon in the NMR solvent. ^{19}F NMR (376.05 MHz) spectra were measured with a JEOL JNM-AL 400 NMR spectrometer in a $CDCl_3$ solution with trichlorofluoromethane ($CFCl_3$, δ_F = 0 ppm) as an internal standard. High-resolution mass spectra (HRMS) were taken on a JEOL JMS-700MS spectrometer with fast atom bombardment (FAB) ionization method.

Materials

All reactions were carried out using dried glassware with a magnetic stirrer bar under an atmosphere of argon and routinely monitored by ^{19}F NMR spectroscopy or thin-layer chromatography (TLC). Ethanol and DMF employed as solvents were freshly distilled using magnesium (Mg) or calcium hydride (CaH_2), respectively, as a drying agent prior to use. All chemicals were of reagent grade and, if necessary, were purified in the usual manner. Column chromatography was carried out on silica gel (Wako gel® C-200) and TLC was performed on silica gel TLC plates (Merck, Silica gel 60 F_{254}).

Typical procedure for the preparation of methyl 2,2,3,3-tetrafluoro-4-oxo-4-[4-(4-propylphenyl)phenyl]butanoate (6b)

To a solution of dimethyl 2,2,3,3-tetrafluorosuccinate (**7**, 7.4 g, 30 mmol) in THF (60 mL) was added a THF solution of 4-(4-propylphenyl)phenylmagnesium bromide, prepared from 4-(4-propylphenyl)phenyl bromide (60 mmol) and magnesium turnings (66 mmol) at −78 °C. Then, the mixture was stirred at that temperature overnight. The reaction mixture was poured into aqueous NH_4Cl solution, then the whole was extracted with EtOAc three times. The combined organic layers were dried over anhydrous Na_2SO_4, filtered, and concentrated in vacuo. The residue was purified by silica gel column chromatography to afford the γ-keto ester (**6b**, 7.7 g, 20 mmol, 67% yield).

Methyl 2,2,3,3-tetrafluoro-4-oxo-4-(4-propylphenyl)butanoate (6a)

Yield 85% (7.1 g, 23 mmol); Yellow liquid; IR (neat): ν 2963, 2935, 2874, 1605, 1569, 1419, 1261, 1132, 1105, 1038, 863, cm^{-1}; HRMS (FAB+) calcd for $C_{14}H_{15}F_4O_3$ $[M+H]^+$: 307.0957, found: 307.0956; ^1H NMR ($CDCl_3$): δ 0.95 (t, J = 7.7 Hz, 3H), 1.68 (sext., J = 7.7 Hz, 2H), 2.68 (t, J = 7.7 Hz, 2H), 3.95 (s, 3H), 7.34 (d, J = 8.4 Hz, 2H), 8.02 (d, J = 8.4 Hz, 2H); ^{13}C NMR ($CDCl_3$): δ 13.5, 24.0, 38.2, 53.8, 108.3 (tt, J = 266.8, 27.5 Hz), 111.3 (tt, J = 266.8, 28.9 Hz), 128.6, 129.2, 130.4, 151.9, 160.4 (t, J = 28.9 Hz), 185.1 (t, J = 27.5 Hz). ^{19}F NMR ($CDCl_3$, $CFCl_3$): δ –121.08 (s, 2F), –113.35 (s, 2F).

Methyl 2,2,3,3-tetrafluoro-4-oxo-4-[4-(4-propylphenyl)phenyl]butanoate (6b).

Yield 67% (7.7 g, 20 mmol); Yellow solid; m.p. = 56–57 °C; IR (KBr): ν 3012, 2958, 2912, 1597, 1436, 1260, 1162, 1107, 950 cm^{-1}; HRMS (FAB) calcd for $C_{20}H_{19}F_4O_3$ $[M+H]^+$: 383.1270, found: 383.1270; ^1H NMR ($CDCl_3$): δ 1.96 (t, J = 7.4 Hz, 3H), 1.69 (sext., J = 7.4 Hz, 2H), 2.65 (t, J = 7.4 Hz, 2H), 3.97 (s, 3H), 7.31 (d, J = 8.4 Hz, 2H), 7.57 (d, J = 8.0 Hz, 2H), 7.75 (d, J = 8.0 Hz, 2H), 7.66 (d, J = 8.4 Hz, 2H).^{13}C NMR ($CDCl_3$): δ 13.8, 24.4, 37.7, 54.0, 108.2 (tt, J = 262.7, 27.8 Hz), 111.2 (tt, J = 270.1, 28.5 Hz), 127.2, 127.3, 129.2, 130.8 (two carbons were overlaped), 136.4, 143.8, 148.2, 160.4 (t, J = 30.0 Hz), 184.9 (t, J = 27.1 Hz); ^{19}F NMR ($CDCl_3$, $CFCl_3$): δ –120.97 (t, J = 4.9 Hz, 2F), –113.46 (t, J = 4.9 Hz, 2F).

Methyl 2,2,3,3-tetrafluoro-4-oxo-4-[4-(*trans*-4-*n*-propylcyclohexyl)phenyl]butanoate (6c)

Yield 86% (4.5 g, 12 mmol); Yellow liquid; IR (KBr) 2955, 2370, 1696, 1165, 1030, 948, 869 cm^{-1}; HRMS (FAB+) calcd for $C_{20}H_{24}F_4O_2$ $[M]^+$: 388.1662, found: 388.1664; ^1H NMR ($CDCl_3$): δ 0.91 (t, J = 7.2 Hz, 3H), 1.07 (m, 2H), 1.20–1.40 (m, 5H), 1.42–1.52 (m, 2H), 1.90 (d, J = 12.0 Hz, 4H), 2.57 (t, J = 12.0 Hz, 1H), 3.95 (s, 3H), 7.37 (d, J = 8.4 Hz, 2H), 8.03 (d, J = 8.4 Hz, 2 H); ^{13}C NMR ($CDCl_3$): δ 14.3, 20.0, 33.4, 33.8, 36.9, 39.7, 45.0, 53.8, 108.3 (tt, J = 269.6, 27.5 Hz), 111.3 (tt, J = 269.5, 28.9 Hz), 127.6, 128.7, 130.5, 156.7, 160.4 (t, J = 28.9 Hz), 185.0 (t, J = 27.49 Hz); ^{19}F NMR ($CDCl_3$, $CFCl_3$): δ = –121.09 (t, J = 4.9 Hz, 2F), –113.37 (t, J = 4.9 Hz, 2F).

Typical procedure for the synthesis of

3-ethenyl-4,4,5,5-tetrafluoro-6-[4-(4-propylphenyl)phenyl]-1,7-octadiene-3,6-diol (5b).

To a solution of β-keto ester (**6b**, 5.8 g, 15 mmol) in ether (150 mL) was added a THF solution of vinylmagnesium chloride (2.1 M, 26 mL, 54 mmol) at room temperature and the reaction was stirred at reflux temperature overnight. Then, the mixture was poured into aqueous NH_4Cl solution and the whole was extracted with ether three times. The combined organic layers were dried over anhydrous Na_2SO_4, filtered, and concentrated in vacuo. The residue was purified by silica gel column chromatography to afford the desired diol (**5b**, 2.8 g, 6.5 mmol, 43% yield), together with the conjugated addition product (**8b**, 3.1 g, 7.1 mmol, 47% yield).

1-Ethenyl-4,4,5,5-tetrafluoro-6-(4-propylphenyl)-1,7-octadiene-3,6-diol (5a)

Yield 46% (3.8 g, 11 mmol); Yellow liquid; IR (neat): ν 3570, 3098, 2961, 1708, 1641, 1152, 1119, 935 cm^{-1}; HRMS (FAB) calcd for $C_{19}H_{21}F_4O_2$ $[M–H]^+$: 357.1478, found: 357.1470; ^1H NMR ($CDCl_3$): δ 0.93 (t, J = 7.5 Hz, 3H), 1.64 (sext., J = 7.5 Hz, 2H), 2.57 (t, J = 7.5 Hz, 2H), 3.30 (s, 1H), 4.24 (s, 1H), 5.36 (d, J = 10.8 Hz, 2H), 5.40 (d, J = 10.8 Hz, 1H), 5.50 (d, J = 17.2 Hz, 1H), 5.51 (d, J = 17.2 Hz, 1H), 5.52 (d, J = 17.2 Hz, 1H), 6.09 (ddd, J = 17.2, 10.8, 1.6 Hz, 1H), 6.17 (dd, J = 17.2, 10.8 Hz, 1H), 6.61 (ddd, J = 17.2, 10.8, 1.6 Hz, 1H), 7.16 (d, J = 8.2 Hz, 2H), 7.46 (d, J = 8.2 Hz, 2H); ^{13}C NMR ($CDCl_3$): δ 13.9, 24.4, 37.7, 77.4 (t, J = 25.2 Hz), 115.9, 117.2, 117.66, 117.71 (tt, J = 265.1, 28.5 Hz), 117.9 (tt, J =265.1, 29.3 Hz), 127.1, 128.1, 133.9 (d, J = 2.5 Hz), 134.6 (d, J = 1.6 Hz), 135.9, 137.1, 142.8, The

peaks of olefinic carbons and tertiary carbons could not be completely identified because they are overlapped with other carbons; ^{19}F NMR ($CDCl_3$, $CFCl_3$) δ –116.75 (d, J = 278.3 Hz, 1F), –116.33 (dd, J = 278.3, 4.9 Hz, 1F), –114.80 (d, J = 278.3 Hz, 1F), –113.48 (d, J = 278.3 Hz).

2-(3-Buten-1-yl)-5-ethenyl-4,4,5,5-tetrafluoro-5-(4-propylphenyl)tetrahydrofuran-2-ol (8a).

Yield 42% (3.5 g, 9.8 mmol); Diastereomeric ratio = *ca.* 1 : 1; Yellow liquid; IR (neat): ν 3588, 3516, 2962, 1643, 1513, 1166, 1000, 938 cm^{-1}; HRMS (FAB) calcd for $C_{19}H_{21}F_4O_2$ [M–H]$^+$: 357.1478, found: 357.1479; ^{1}H NMR ($CDCl_3$): δ 0.96 (t, J = 7.4 Hz, 3H), 1.65 (sext., J = 7.4 Hz, 2H), 2.01–2.13 (m, 2H), 2.45–3.28 (m, 4H), 3.00–3.27 (m, 1H), 5.50–5.97 (m, 6H), 7.23–7.39 (m, 4H); ^{13}C NMR ($CDCl_3$): δ 13.9, 24.5, 26.6, 33.3 and 34.3, 37.8, 84.3 (td, J = 22.7, 3.3 Hz) and 84.6 (td, J = 23.1, 3.3 Hz), 100.4 (ddd, J = 28.5, 20.7, 3.3 Hz) and 101.1 (ddd, J = 28.5, 20.7, 3.3 Hz), 115.5 (2C Overlapped) and 115.8 (2C Overlapped), 116.9 (2C Overlapped) and 117.6 (2C Overlapped), 125.5, 128.57 and 128.59, 133.9 and 134.5, 135.5 and 136.5, 137.7 and 137.8, 143.0 and 143.1; ^{19}F NMR ($CDCl_3$, $CFCl_3$): δ –132.84 (dd, J = 244.1, 14.7 Hz, 1F) and□–132.54 (ddd, J = 241.8, 14.7, 7.1 Hz, 1F),□–128.16 (dd, J = 245.2, 12.0 Hz, 1F)□and –123.83 (dd, J = 241.8, 14.7 Hz, 1F), –126.54 (dd, J = 241.8, 14.7 Hz, 1F) and –122.54 (dd, J = 244.1, 14.7 Hz, 1F), –119.89 (dd, J = 241.8, 12.0 Hz, 1F) and –110.80 (dd, J = 245.2, 14.7 Hz, 1F).

3-Ethenyl-4,4,5,5-tetrafluoro-6-[4-(4-propylphenyl)phenyl]-1,7-octadiene-3,6-diol (5b).

Yield 43% (2.8 g, 6.5 mmol); Yellow liquid; IR (neat): ν 3349, 2960, 1497, 1120, 1005, 936 cm^{-1}; HRMS (FAB+): calcd for $C_{25}H_{26}F_4O_2$ [M]$^+$: 434.1869, found: 434.1875; ^{1}H NMR ($CDCl_3$): δ 1.03 (t, J = 7.4 Hz, 3H), 1.73 (sext., J = 7.4 Hz, 2H), 2.68 (t, J = 7.4 Hz, 2H), 3.75 (s, 1H), 4.75 (s, 1H), 5.42 (d, J = 10.8 Hz, 1H), 5.43 (d, J = 10.8 Hz, 1H), 5.45 (d, J = 10.8 Hz, 1H), 5.57 (d, J = 17.2 Hz, 2H), 5.58 (d, J = 17.2 Hz, 1H), 6.15 (dd, J = 17.2, 10.8 Hz, 1H), 6.23 (dd, J = 17.2, 10.8 Hz, 1H), 6.69 (dd, J = 17.2, 10.8 Hz, 1H), 7.30 (d, J = 8.4 Hz, 2H), 7.56 (d, J = 8.4 Hz, 2H), 7.62 (d, J = 8.4 Hz, 2H), 7.68 (d, J = 8.4 Hz, 2H); ^{13}C NMR ($CDCl_3$): δ 13.8, 24.5, 37.6, 77.27 (t, J = 24.9 Hz), 77.33 (t, J = 24.9 Hz), 116.0, 117.1, 117.4, 117.6 (tt, J = 265.0, 28.5 Hz), 117.8 (tt, J = 264.3, 29.2 Hz), 126.3, 126.9, 127.4, 128.8, 133.7, 134.5, 137.1, 137.3, 137.8, 140.8, 142.0; ^{19}F NMR ($CDCl_3$, $CFCl_3$): δ –116.23 to –116.19 (m,

2F), –114.45 (d, J = 278.3 Hz, 1F), –113.36 (d, J = 278.3 Hz, 1F).

2-(3-Buten-1-yl)-5-ethenyl-4,4,5,5-tetrafluoro-5-[4-(4-propylphenyl)phenyl]tetrahydrofuran-2-ol (8b).

Yield 47% (3.1 g, 7.1 mmol); Yellow liquid; Diastereomeric ratio = *ca.* 1 : 1; IR (neat): ν 3515, 2961, 2871, 1643, 1497, 1331, 1166, 1003 cm^{-1}; HRMS (FAB) calcd for $C_{25}H_{26}F_4O_2$ $[M]^+$: 434.1869, found: 434.1874; ^1H NMR ($CDCl_3$): δ 0.99 (t, J = 7.3 Hz, 3H), 1.70 (sext., J = 7.3 Hz, 2H), 2.0–2.2 (m, 2H), 2.45–2.55 (m, 2H), 2.65 (t, J = 7.3 Hz, 2H), [3.05 (s, 0.5H) and 3.30 (d, J = 3.2 Hz, 0.5H)], 5.05–5.25 (m, 2H), [5.33 (d, J = 10.4 Hz, 0.5H) and 5.42 (d, J = 10.8 Hz, 0.5H)], [5.37–5.45 (m, 0.5H) and 5.60–5.68 (m, 0.5H)], 5.90–6.30 (m, 1H), [6.20 (ddd, J = 16.8, 10.4, 2.4 Hz, 0.5H) and 6.45 (ddd, J = 16.8, 10.8, 2.0 Hz)], 7.27 (d, J = 7.6 Hz, 2H), 7.45–7.55 (m, 4H), 7.60–7.64 (m, 2H); ^{13}C NMR ($CDCl_3$): δ 13.8, 24.5, [26.36 and 26.41], [33.1 and 34.1], 37.7, [84.1 (t, J = 22.7 Hz) and 84.5 (t, J = 24.2 Hz)], [100.0–100.6 (m) and 100.7–101.3 (m)], 112.9–120.6 (m, 2C), [115.5 and 115.8], [117.1 and 117.8], [125.8 and 125.9], 126.9, 128.9, [135.1 and 135.2], [135.6 and 135.7], 136.2, [137.5 and 137.63], [137.68 and 137.71], [141.2 and 141.3], 142.2; ^{19}F NMR ($CDCl_3$, $CFCl_3$): δ [–132.78 (dd, J = 243.7, 13.5 Hz, 1F) and –126.58 (dd, J = 243.7, 13.5 Hz, 1F)], [–132.61 (ddd, J = 242.2, 15.0, 6.8 Hz, 1F) and –127.87 (dm, J = 242.2 Hz, 1F)], [–123.54 (dd, J = 240.7, 13.5 Hz, 1F) and –119.8 (dd, J = 240.7, 13.5 Hz, 1F)] [–122.73 (dd, J = 243.7, 12.8 Hz, 1F) and –110.62 (dd, J = 243.7, 13.9 Hz, 1F)].

3-Ethenyl-4,4,5,5-tetrafluoro-6-[4-(*trans*-4-propylcyclohexyl)phenyl]-1,7-octadiene-3,6-diol (5c)

Yield 38% (1.9 g, 4.4 mmol); Yellow liquid; IR (neat): ν 2921, 2850, 1641, 1513, 1414, 1122, 997, 935, 853 cm^{-1}; HRMS (FAB+) calcd for $C_{25}H_{32}F_4NaO_2$ $[M+Na]^+$: 463.2236, found: 463.2226; ^1H NMR ($CDCl_3$): δ 0.90 (t, J = 7.2 Hz, 3 H), 0.98–1.08 (m, 2H), 1.18–1.37 (m, 5H), 1.38–1.48 (m, 2H), 1.87 (t, J = 10.8 Hz, 4H), 2.45 (tt, J = 12.2, 3.1 Hz, 1H), 3.33 (s, 1H), 4.25 (s, 1H), 5.36 (d, J = 10.8 Hz, 2H), 5.40 (d, J = 10.8 Hz, 1H), 5.50 (d, J = 17.2 Hz, 1H), 5.51 (d, J = 17.2 Hz, 1H), 5.52 (d, J = 17.2 Hz, 1H), 6.10 (ddd, J = 17.2, 10.8, 2.0 Hz, 1 H), 6.17 (dd, J = 17.2, 10.8 Hz, 1H), 6.61 (dd, J = 17.2, 10.8 Hz, 1H), 7.19 (d, J = 8.4 Hz, 2H), 7.46 (d, J = 8.4 Hz, 2H); ^{13}C NMR ($CDCl_3$): δ 14.5, 20.1, 33.6, 34.3, 37.1, 39.8, 44.2, 77.3 (t, J = 24.4 Hz), 77.3 (t, J = 24.4 Hz), 115.8, 117.1, 117.7 (tt, J = 265.4, 28.7 Hz), 117.7,

117.9 (tt, J = 265.4, 28.7 Hz), 126.4, 127.1, 133.9, 134.5, 136.0, 137.0, 147.8; ^{19}F NMR ($CDCl_3$, $CFCl_3$): δ –116.78 (d, J = 278.3 Hz, 1F), –116.00 (dd, J = 278.3, 7.5 Hz, 1F), –114.86 (d, J = 278.3 Hz, 1F), –113.46 (dt, J = 278.3, 7.1 Hz, 1F).

2-(3-Buten-1-yl)-5-ethenyl-4,4,5,5-tetrafluoro-5-[4-(*trans*-4-propylcyclohexyl)phenyl]tetrahydrofuran-2-ol (8c)

Yield 46% (2.4 g, 5.3 mmol); Diastereomeric ratio = *ca.* 1 : 1; Yellow liquid; IR (neat): ν 3590, 3080, 2922, 1643, 1513, 1448, 1331, 1166, 999, 821 cm^{-1}; HRMS (FAB+) calcd for $C_{25}H_{32}F_4O_2$ $[M]^+$: 440.2338, found: 440.2342; ^{1}H NMR ($CDCl_3$): δ 0.91 (t, J = 7.2 Hz, 3H), 1.01–1.10 (m, 2H), 1.20–1.39 (m, 5H), 1.40–1.50 (m, 2H), 1.88 (t, J = 10.0 Hz, 4H), 2.00–2.14 (m, 2H), 2.46 (m, 3H), 3.24 (s, 1H) and 3.25 (s, 1H), 5.05–5.62 (m, 4H), 5.90–5.99 (m, 1H), 6.12–6.45 (m, 1H), 7.22–7.38 (m, 4H); ^{13}C NMR ($CDCl_3$): δ 14.7, 20.4, 26.7, 33.4, 33.9, 34.6, 37.4, 40.1, 44.6, 84.5 (t, J = 22.3 Hz) and 84.8 (dt, J = 22.3, 3.3 Hz), 100.6 (ddd, J = 25.7, 21.5, 3.3 Hz) and 101.3 (ddd, J = 24.8, 19.9, 3.3 Hz), 113.0–121.5 (m, 2C), 115.8 and 116.0, 117.0 and 117.7, 125.6, 127.2, 134.2 (d, J = 2.0 Hz) and 134.7 (d, J = 2.5 Hz), 135.7 (d, J = 5.8 Hz) and 136.7 (d, J = 3.3 Hz), 137.9 and 138.0, 148.35 and 148.41, The peaks of methylene carbons could not be completely identified because they are overlapped with other carbons; ^{19}F NMR ($CDCl_3$, $CFCl_3$): δ –132.90 (dd, J = 241.8, 14.7 Hz, 1F) and –132.57 (ddd, J = 236.5, 14.7, 7.5 Hz, 1F), –128.09 (ddd, J = 236.5, 14.7, 4.9 Hz, 1F) and –123.90 (dd, J = 242.3, 14.7 Hz, 1F), –126.52 (dd, J = 243.0, 14.7 Hz, 1F) and –122.42 (dd, J = 241.9, 14.7 Hz, 1F), –119.89 (dd, J = 242.3, 14.7 Hz, 1F) and –110.71 (dd, J = 243.0, 14.7 Hz, 1F).

Typical procedure for the preparation of *cis*-1-ethenyl-5,5,6,6-tetrafluoro-4-[4-(4-propylphenyl)phenyl]-2-cyclohexene-1,4-diol (4b)

To a solution of 10 mol % of Grubbs' 1st generation catalyst (0.37 g, 0.50 mmol) in CH_2Cl_2 (45 mL) was added 1,7-octadiene-3,6-diol (**5b**, 2.0 g, 4.6 mmol) at room temperature and stirred at that temperature for 40 h. Then, the reaction mixture was passed through a short plug of silica gel using EtOAc as an eluent and concentrated in vacuo to give the crude material. Purification by silica gel column chromatography afforded the desired product **4b** (1.1 g, 2.7 mmol, 59% yield).

***cis*-1-Ethenyl-5,5,6,6-tetrafluoro-4-(4-propylphenyl)-2-cyclohexene-1,4-diol (4a).**

Yield 75% (2.7 g, 8.0 mmol); Single isomer; Brown solid; m.p.: 80.8–81.5 °C; IR (KBr): ν 3366, 2960, 2872, 1638, 1414, 1294, 1172, 1094, 985, 876 cm^{-1}; HRMS (FAB) calcd for $C_{17}H_{18}F_4O_2$ $[M]^+$: 330.1243, found: 330.1248; ^1H NMR ($CDCl_3$): δ 0.98 (t, *J* = 7.2 Hz, 3H), 1.68 (sext., *J* = 7.2 Hz, 2H), 2.63 (t, *J* = 7.2 Hz 2H), 3.41 (s, 1H), 3.55 (s, 1H), 5.45 (d, *J* = 10.8 Hz, 1H), 5.63 (d, *J* = 17.6 Hz, 1H), 5.90 (dd, *J* = 10.8, 5.2 Hz, 1H), 5.99–6.05 (m, 2H), 7.23 (d, *J* = 8.0 Hz, 2H), 7.39 (d, *J* = 8.0 Hz, 2H); ^{13}C NMR ($CDCl_3$): δ 13.9, 24.4, 37.7, 60.9, 74.5–75.0 (m), 113.8 (dddd, *J* = 265.7, 253.7, 28.1, 22.4 Hz), 115.2 (dddd, *J* = 265.7, 252.1, 30.5, 23.2 Hz), 119.0, 127.4 (d, *J* = 1.6 Hz), 128.3, 130.7 (d, *J* = 2.5 Hz), 131.5 (d, *J* = 4.1 Hz), 133.5, 133.6 (d, *J* = 4.1 Hz), 143.7; ^{19}F NMR ($CDCl_3$, $CFCl_3$): δ –132.08 (dd, *J* = 265.4, 19.6 Hz, 1F), –130.64 (dd, *J* = 265.4, 17.1 Hz, 1F), –122.25 (dd, *J* = 265.4, 17.1 Hz, 1F), –116.11 (ddd, *J* = 265.4, 19.6, 4.9 Hz, 1F).

***cis*-1-Ethenyl-5,5,6,6-tetrafluoro-4-[4-(4-propylphenyl)phenyl]-2-cyclohexene-1,4-diol (4b).**

Yield 59% (1.1 g, 2.7 mmol); Single isomer; Brown solid; m.p.: 139–140 °C; IR (KBr): ν 3359, 3027, 2956, 1655, 1499, 1356, 1243, 1149, 1109, 1098, 986, 885 cm^{-1}; HRMS (FAB+) calcd for $C_{23}H_{22}F_4O_2$ $[M]^+$: 406.1556, found: 406.1553; ^1H NMR ($CDCl_3$): δ 0.98 (t, *J* = 7.2 Hz, 3H), 1.68 (sext., *J* = 7.2 Hz, 2H), 2.59 (d, *J* = 5.2 Hz, 1H), 2.63 (t, *J* = 7.2 Hz, 2H), 2.74 (d, *J* = 4.0 Hz, 1H), 5.49 (d, *J* = 10.7 Hz, 1H), 5.67 (d, *J* = 16.8 Hz, 1H), 5.92–6.09 (m, 3H), 7.26 (d, *J* = 8.0 Hz, 2H), 7.50–7.53 (m, 4H), 7.61 (d, *J* = 8.8 Hz, 2H); ^{13}C NMR ($CDCl_3$): δ 13.8, 24.5, 37.7, 74.4–75.0 (m, 2C), 111.0–119.1 (m, 2C), 126.6, 127.0, 127.8, 128.9, 130.7, 131.65, 131.69, 133.4 (t, *J* = 5.0 Hz), 134.6, 137.6, 141.9, 142.3; ^{19}F NMR ($CDCl_3$, $CFCl_3$): δ –132.24 to –131.45 (m, 1F), –130.93 to –130.17 (m, 1F), –122.33 (ddd, *J* = 263.6, 19.6, 4.9 Hz, 1F), –116.18 (ddd, *J* = 268.5, 17.3, 6.1 Hz, 1F).

***cis*-1-Ethenyl-5,5,6,6-tetrafluoro-4-[4-(*trans*-4-propylcyclohexyl)phenyl]-2-cyclohexene-1,4-diol (4c)**

Yield 71% (1.3 g, 3.1 mmol); Single isomer; Brown solid; m.p.: 144.2–145.5 °C; IR (KBr): ν 3348, 2922, 2859, 1655, 1509, 1446, 1150, 1007, 965, 878 cm^{-1}; HRMS (FAB+) calcd for $C_{23}H_{28}F_4NaO_2$ $[M+Na]^+$: 435.1923, found: 435.1932; ^1H NMR ($CDCl_3$): δ 0.90 (t, *J* = 7.2 Hz, 3H), 0.99–1.10 (m, 2H),

1.18–1.38 (m, 5H), 1.39–1.49 (m, 2H), 1.88 (t, *J* = 10.4 Hz, 4H), 2.48 (tt, *J* = 12.6, 3.6 Hz, 1H), 2.59 (d, *J* = 2.8 Hz, 1H), 2.69 (d, *J* = 3.6 Hz, 1H), 5.46 (d, *J* = 10.8 Hz, 1H), 5.64 (d, *J* = 16.8 Hz, 1H), 5.91 (ddd, *J* = 9.2, 5.2, 1.6 Hz, 1H), 5.98–6.05 (m, 2H), 7.24 (d, *J* = 8.2 Hz, 2H), 7.37 (d, *J* = 8.2 Hz, 2H); ^{13}C NMR ($CDCl_3$): δ 14.5, 20.2, 33.6, 34.30, 34.33, 37.1, 39.8, 44.4, 74.7 (d, *J* = 20.7 Hz), 75.0 (d, *J* =19.8 Hz), 114.1 (dddd, *J* = 265.8, 242.6, 30.2, 22.7 Hz), 115.3 (dddd, *J* = 265.8, 242.6, 30.2, 22.7 Hz), 119.1, 126.8, 127.1, 127.4, 131.0 (d, *J* = 2.5 Hz), 131.6 (d, *J* = 2.2 Hz), 133.6 (t, *J* = 5.0 Hz), 149.0: The peak of methylene groups could not be identified because they were overlapping with other carbon signals; ^{19}F NMR ($CDCl_3$, $CFCl_3$): δ –132.10 (dd, *J* = 264.2, 19.6 Hz, 1F),–130.73 (dd, *J* = 264.2, 19.6 Hz, 1F), –122.24 (dd, *J* = 264.2, 17.3 Hz, 1F), –166.02 (ddd, *J* = 264.2, 19.6, 7.1 Hz, 1F).

Typical procedure for the preparation of *cis*-1-ethyl-2,2,3,3-tetrafluoro-4-[4-(4-propylphenyl)phenyl]cyclohexane-1,4-diol (3b)

To a solution of 10% Pd/C (0.76 g, 0.72 mmol) in MeOH (150 mL) was added the RCM product **4b** (1.5 g, 3.6 mmol) at room temperature. The reaction flask was filled with hydrogen and the mixture stirred at room temperature for over 1 day. Then, the reaction mixture was passed through a short plug of silica gel using EtOAc as an eluent and concentrated in vacuo to give the crude material. Purification by silica gel column chromatography afforded the desired product **3b** (1.4 g, 3.4 mmol, 96% yield).

***cis*-1-Ethyl-2,2,3,3-tetrafluoro-4-(4-propylphenyl)cyclohexane-1,4-diol (3a)**

Yield: 99% (2.7 g, 8.0 mmol); White solid; m.p. = 104.2–104.8 °C; IR (KBr) 3388, 2964, 2874, 1618, 1466, 1275, 1125, 1045, 982 cm^{-1}; HRMS (FAB+) calcd for $C_{17}H_{22}F_4O_2$ $[M]^+$: 334.1556, found: 334.1555; ^{1}H NMR ($CDCl_3$): δ 0.95 (t, *J* = 7.2 Hz, 3H), 1.04 (t, *J* = 7.2 Hz, 3H), 1.65 (sext., *J* = 7.2 Hz, 2H), 1.85 (q, *J* = 7.2 Hz, 2H), 1.19–2.03 (m, 3H), 2.10 (t, *J* = 14.0 Hz, 1H), 2.23 (t, *J* = 13.2 Hz, 1H), 2.60 (t, *J* = 7.6 Hz, 3H), 7.22 (d, *J* = 7.8 Hz, 2H), 7.45 (d, *J* = 7.8 Hz, 2H); ^{13}C NMR ($CDCl_3$): δ 6.6, 14.0, 23.8, 24.5, 26.7 (d, *J* = 4.1 Hz), 30.3 (d, *J* = 4.1 Hz), 37.7, 74.8–75.5 (m, 2C), 115.4 (dddd, *J* = 268.2, 247.3, 28.9, 21.0 Hz), 117.3 (dddd, *J* = 268.2, 247.3, 28.9, 21.0 Hz), 126.6, 128.5, 135.5, 143.5; ^{19}F NMR ($CDCl_3$, $CFCl_3$): δ –135.05 (d, *J* = 274.5 Hz, 1F), –132.63 (d, *J* = 258.7 Hz, 1F), –117.79 (d, *J* = 258.7 Hz 1F), –113.74 (d, *J* = 274.5 Hz, 1F).

***cis*-1-Ethyl-2,2,3,3-tetrafluoro-4-[4-(4-propylphenyl)phenyl]cyclohexane-1,4-diol (3b)**

[Known compound] Yield 96% (1.4 g, 3.4 mmol); White solid; m.p.: 139–140 °C; Analytical data (^{1}H NMR, ^{13}C NMR, ^{19}F NMR, IR, and m.p.) were in excellent agreement with reported data [1].

***cis*-1-Ethyl-2,2,3,3-tetrafluoro-4-[4-(*trans*-4-propylcyclohexyl)phenyl]cyclohexane-1,4-diol (3c)**

[Known compound] Yield 100% (1.3 g, 3.1 mmol); White solid; m.p.: 110–111 °C; Analytical data (^{1}H NMR, ^{13}C NMR, ^{19}F NMR, IR, and m.p.) were in excellent agreement with reported data [1].

Typical procedure for the synthesis of

1-ethyl-5,5,6,6-tetrafluoro-4-[4-(*trans*-4-propylcyclohexyl)phenyl]-1,3-cyclohexadiene (1c) [1]

To a solution of the diol **3c** (78 mg, 0.19 mmol) in pyridine (5 mL) was added dropwise phosphorus oxychloride (0.2 mL, 2.0 mmol) at room temperature. Then, the reaction mixture was heated at 90 °C for 24 h. After cooling of the reaction mixture to room temperature, the mixture was poured into aqueous HCL (3 N) and extracted with ether three times. The combined organic layers were dried over anhydrous Na_2SO_4, filtered, and concentrated in vacuo. The residue was purified by silica gel column chromatography to give the corresponding tetrafluorocyclohexadiene derivative **1c** (55 mg, 0.14 mmol, 74% yield).

1-Ethyl-5,5,6,6-tetrafluoro-4-(4-propylphenyl)-1,3-cyclohexadiene (1a)

Yield 82% (2.0 g, 6.5 mmol); Colorless liquid; IR (neat): ν 3031, 2965, 2874, 1611, 1513, 1464, 1268, 1128, 1077, 907, 885 cm^{-1}; HRMS (FAB+) calcd for $C_{17}H_{18}F_4$ $[M]^+$: 298.1345, found: 298.1348; ^{1}H NMR ($CDCl_3$): δ 0.97 (t, J = 7.5 Hz, 3H), 1.19 (t, J = 7.3 Hz, 3H), 1.66 (sext., J = 7.5 Hz, 2H), 2.39 (q, J = 7.3, 2H), 2.63 (t, J = 7.5 Hz, 2H), 6.07 (d, J = 6.2 Hz, 1H), 6.32 (d, J = 6.2 Hz, 1H), 7.21 (d, J = 8.4 Hz, 2H), 7.39 (d, J = 8.4 Hz, 2H); ^{13}C NMR ($CDCl_3$): δ 11.6, 13.9, 21.8, 24.6, 37.9, 114.2 (tt, J = 251.4, 26.7 Hz), 114.3 (tt, J = 251.4, 26.7 Hz), 123.4 (tt, J = 9.1, 2.5 Hz), 125.6 (tt, J = 9.1, 2.5 Hz), 127.1, 128.8, 130.6, 134.1 (t, J = 22.1 Hz), 137.0 (t, J = 22.1 Hz), 143.8; ^{19}F NMR ($CDCl_3$, $CFCl_3$): δ –126.63 (d, J = 4.9 Hz, 2F), –123.30 (d, J = 7.5 Hz, 2F).

4-Ethyl-5,5,6,6-tetrafluoro-1-[4-(4-*n*-propylphenyl)phenyl]cyclohexa-1,3-diene (1b)

[Known compound] Yield 96%; m.p.: 103–105 °C; Analytical data (^{1}H NMR, ^{13}C NMR, ^{19}F NMR, IR, and m.p.) were in excellent agreement with reported dat [1].

1-Ethyl-5,5,6,6-tetrafluoro-4-[4-(*trans*-4-propylcyclohexyl)phenyl]-1,3-cyclohexadiene (1c)

[Known compound] Yield 74%; m.p.: 62–63 °C; Analytical data (^{1}H NMR, ^{13}C NMR, ^{19}F NMR, IR, and m.p.) were in excellent agreement with reported dat [1].

Typical procedure for the synthesis of

***trans*-1-ethyl-2,2,3,3-tetrafluoro-4-[4-(4-propylphenyl)phenyl]cyclohexane (2b)** [2]

Cyclohexane-1,4-diol (**3b**, 0.16 g, 0.38 mmol) was added to flask containing NaH (0.15 g, 3.8 mmol, 60% dispersion oil) in THF (5.0 mL) and stirred for 30 min at room temperature. Then, CS_2 (0.23 mL, 3.8 mmol) was added to the reaction mixture and stirred for further 2 h at room temperature. After the addition of MeI (0.23 mL, 3.8 mmol) to the mixture and stirring for another 2 h the reaction was quenched with water and extracted with Et_2O three times. The combined organic layers were washed with brine, dried over anhydrous Na_2SO_4 and concentrated in vacuo. The residue was dissolved in CH_2Cl_2 (2.4 mL), then Bu_3SnH (0.40 mL, 1.5 mmol) and Et_3B (1.0 mol/L in hexane, 0.72 mL, 0.72 mmol) were added to the flask at room temperature. After stirring for 24 h, the reaction was quenched with water and extracted with CH_2Cl_2 three times. The combined organic layers were washed with brine, dried over anhydrous Na_2SO_4 and concentrated in vacuo. The residue was purified by silica gel column chromatography (hexane/ethyl acetate 20:1) to obtain the corresponding cyclohexane **2b** as a diastereomeric mixture. The mixture was purified by threefold recrystallization from MeOH/Et_2O to afford the pure *trans*-isomer (0.038 g, 0.099 mmol, *trans*/*cis* = 100/0, 25% yield).

1-Ethyl-2,2,3,3-tetrafluoro-(4-propylphenyl)cyclohexane (2a)

Yield 47% (0.055 g, 0.182 mmol); *trans* : *cis* = 79 : 21; Colorless liquid; IR (neat): ν 2961, 2874, 1516, 1459, 1284, 1146, 1063, 1014 cm^{-1}; HRMS (FAB+): calcd for $[M]^+$ $C_{17}H_{22}F_4$: 302.1658, Found: 302.1666; ^{1}H NMR ($CDCl_3$): δ 0.96 (t, J = 7.2 Hz, 3H), 1.03 (t, J = 7.6 Hz, 3H), 1.25–1.45 (m, 2H),

1.65 (sext., J = 7.6 Hz, 2H), 1.75–2.30 (m, 5H), 2.59 (t, J = 7.6 Hz, 2H), 3.06–□.28 (m, 1H), 7.17 (d, J = 8.0 Hz, 2H), 7.23 (d, J = 8.0 Hz, 2H); ^{13}C NMR ($CDCl_3$): (*tran*s isomer) δ 11.6, 13.9, 19.5, 24.5, 26.0, 27.6, 37.7, 42.7 (t, J = 19.7 Hz), 46.7 (t, J = 19.8 Hz), 114.0□122.5 (m, 2C), 128.4, 129.1, 132.3, 142.4; (*ci*s isomer) δ 12.5, 13.6, 18.2, 23.9, 26.8, 27.8, 43.8 (t, J = 19.6 Hz); ^{19}F NMR ($CDCl_3$, $CFCl_3$): (*trans* isomer)□ δ –129.0 to –128.1 (m, 1F), –128.0 to –127.2 (m, 1F), –125.9 to –125.8 (m, 2F); (*cis* isomer) δ –127.3 to –126.5 (m, 2F).

***trans*-1-Ethyl-2,2,3,3-tetrafluoro-4-[4-(*trans*-4-propylphenyl)phenyl]cyclohexane (2b)**

[Known compound] m.p.: 117–118 °C ; Analytical data (^{1}H NMR, ^{13}C NMR, ^{19}F NMR, IR, and m.p.) were in excellent agreement with reported data [2].

***trans*-1-Ethyl-2,2,3,3-tetrafluoro-4-[4-(*trans*-4-propylcyclohexyl)phenyl]cyclohexane (2c)**

[Known compound] m.p.: 95–96 °C; Analytical data (^{1}H NMR, ^{13}C NMR, ^{19}F NMR, IR, and m.p.) were in excellent agreement with reported data [2].

[References]

[1] Yamada, S.; Hashishita, S.; Asai, T.; Ishiahra, T.; Konno, T. *Org. Biomol. Chem.* **2017**, *15*, 1495–1509.

[2] Yamada, S.; Hashishita, S.; Konishi, H.; Nishi, Y.; Kubota, T.; Asai, T.; Ishihara, T.; Konno, T. *J. Fluorine Chem.* **2017**, *200*, 47–58.

*^{1}H NMR spectrum of methyl 2,2,3,3-tetrafluoro-4-oxo-4-(4-propylphenyl)butanoate (**6a**)*

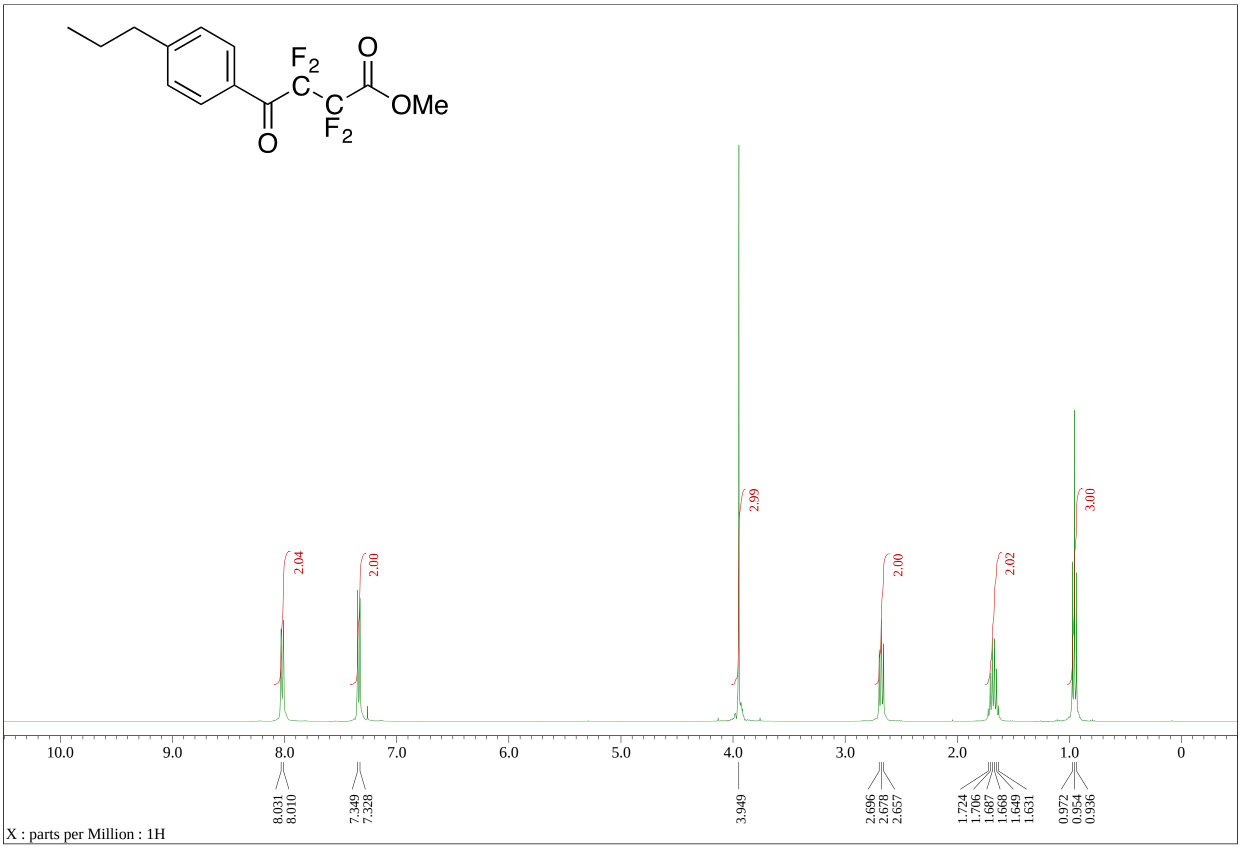

*^{13}C NMR spectrum of methyl 2,2,3,3-tetrafluoro-4-oxo-4-(4-propylphenyl)butanoate (**6a**)*

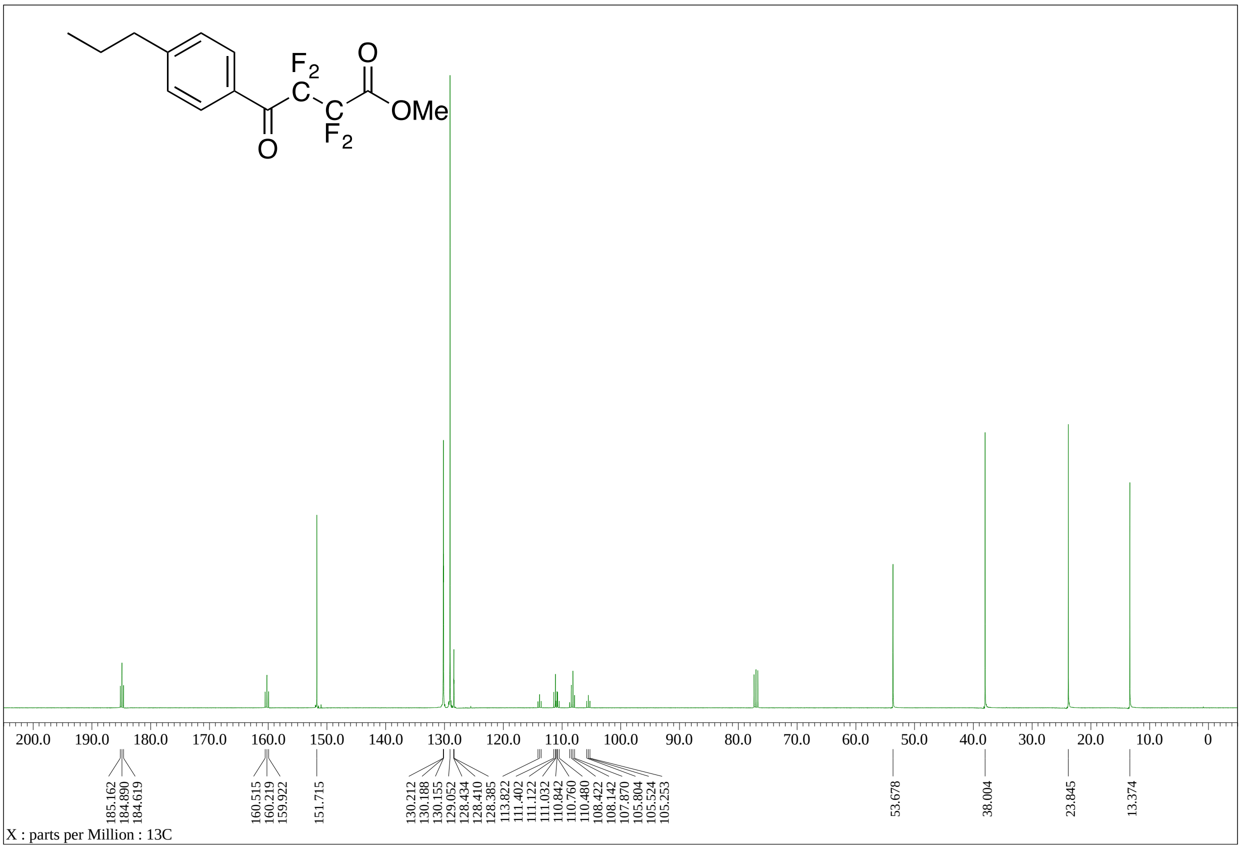

*^{19}F NMR spectrum of methyl 2,2,3,3-tetrafluoro-4-oxo-4-(4-propylphenyl)butanoate (**6a**)*

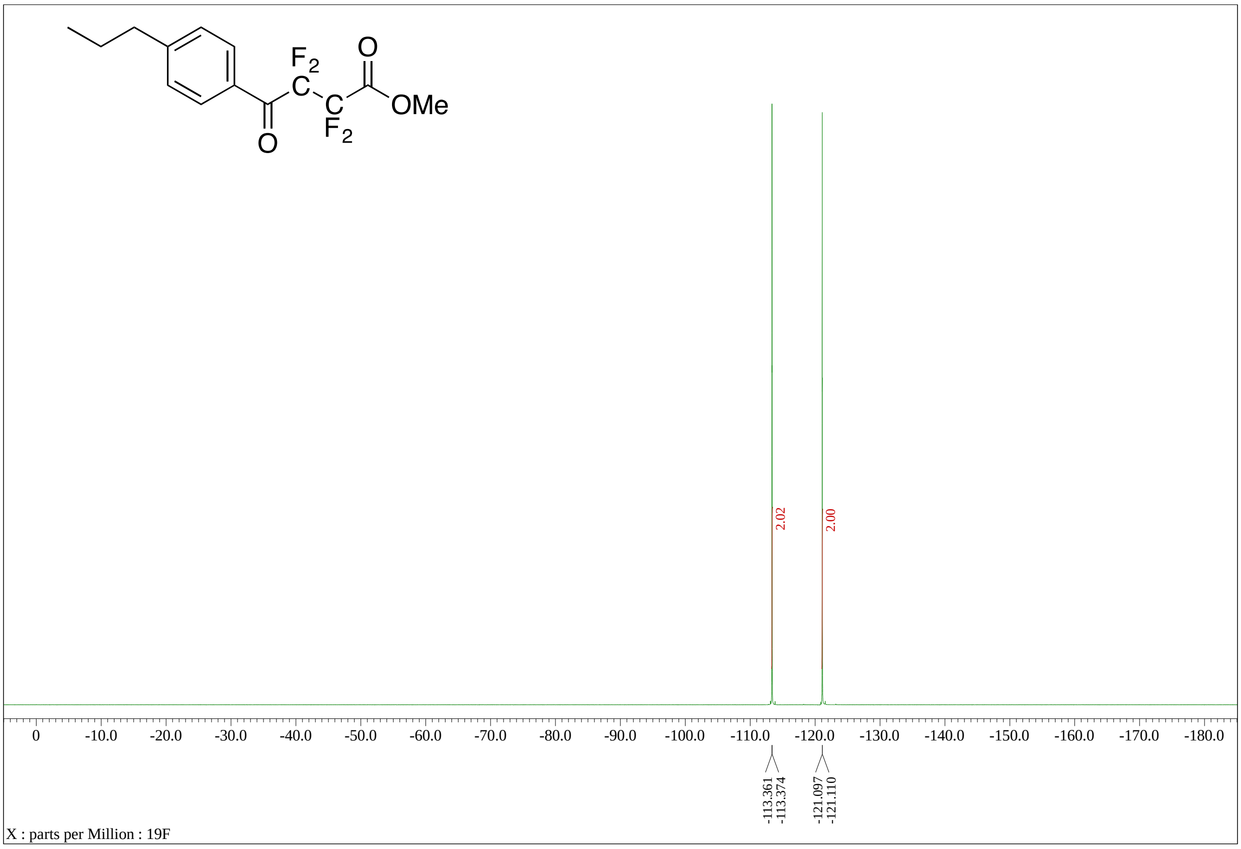

*^{1}H NMR spectrum of methyl 2,2,3,3-tetrafluoro-4-oxo-4-[4-(4-propylphenyl)phenyl]butanoate (**6b**)*

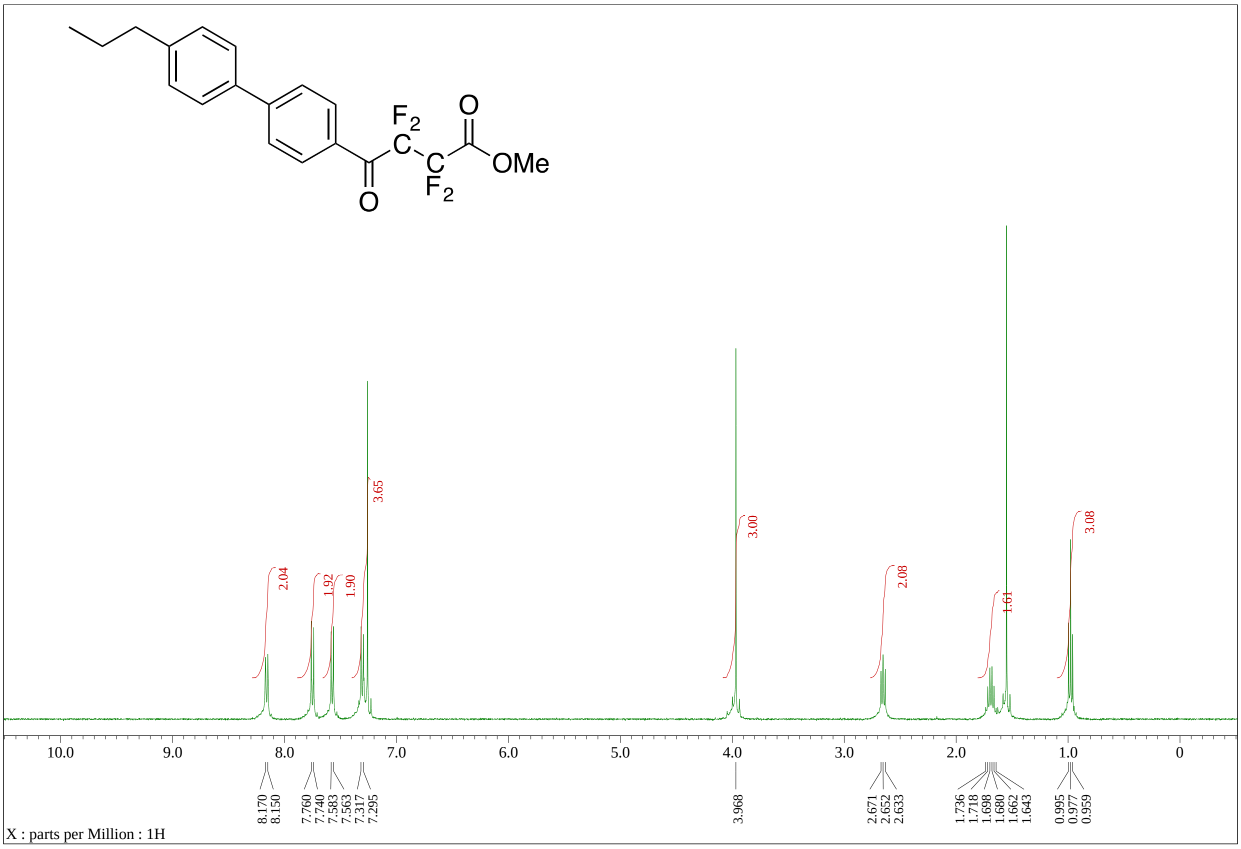

*^{13}C NMR spectrum of methyl 2,2,3,3-tetrafluoro-4-oxo-4-[4-(4-propylphenyl)phenyl]butanoate (**6b**)*

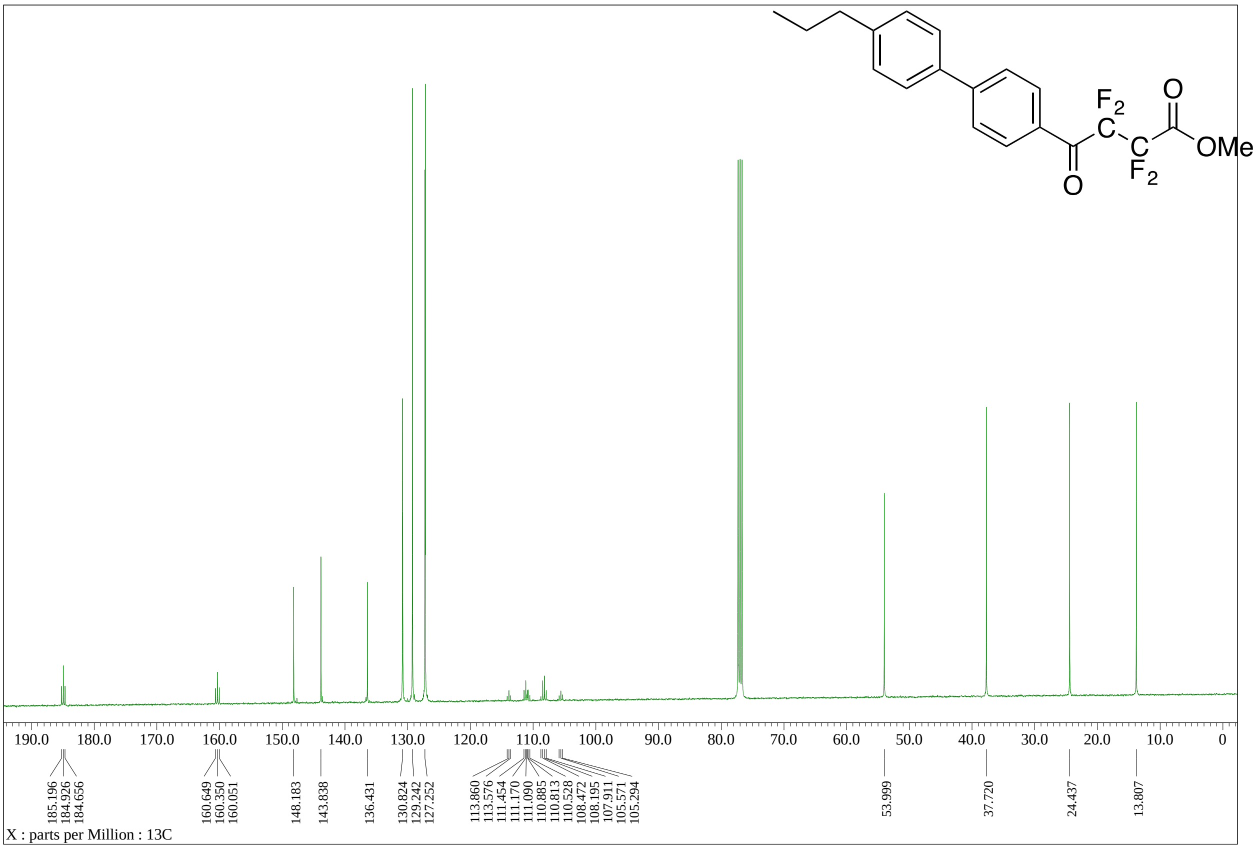

*^{19}F NMR spectrum of methyl 2,2,3,3-tetrafluoro-4-oxo-4-[4-(4-propylphenyl)phenyl]butanoate (**6b**)*

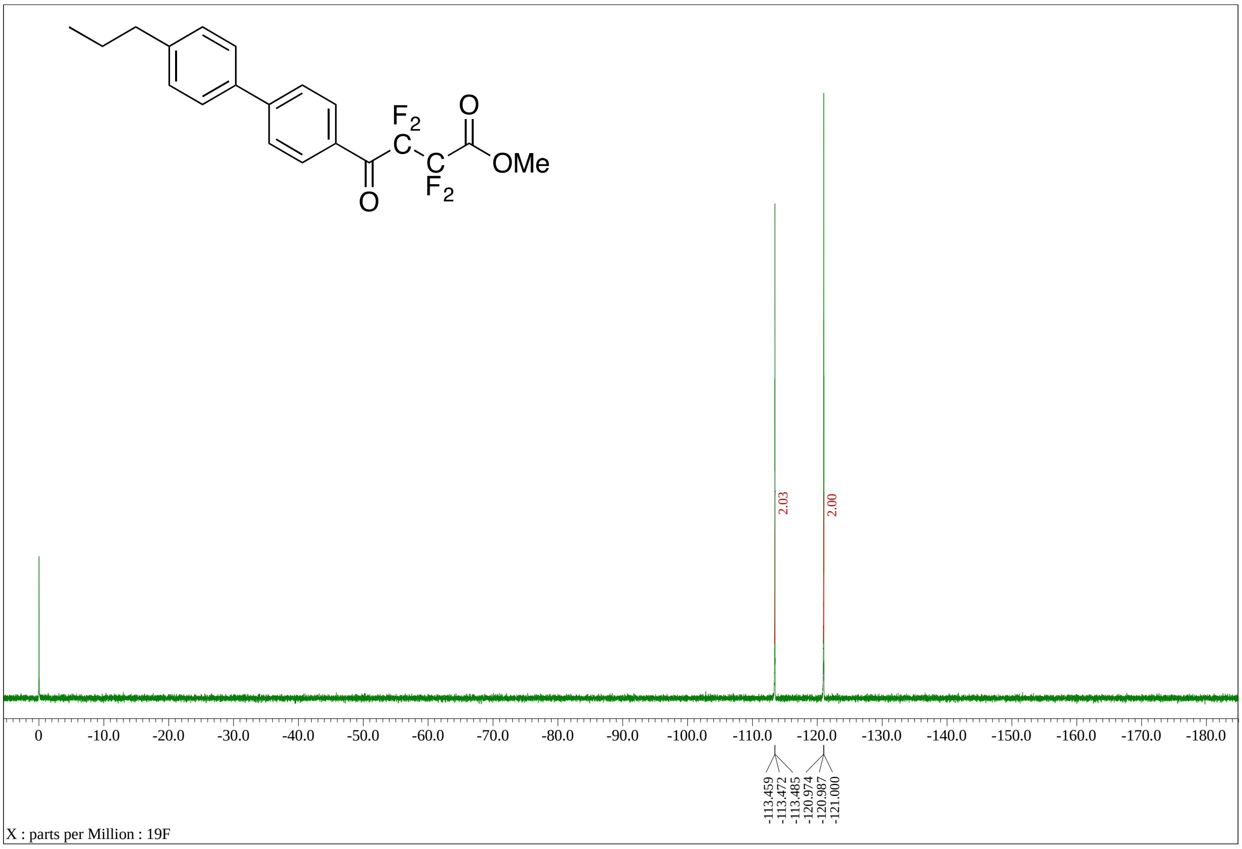

*^{1}H NMR spectrum of methyl 2,2,3,3-tetrafluoro-4-oxo-4-[4-(trans-4-propylcyclohexyl)phenyl]butanoate (**6c**)*

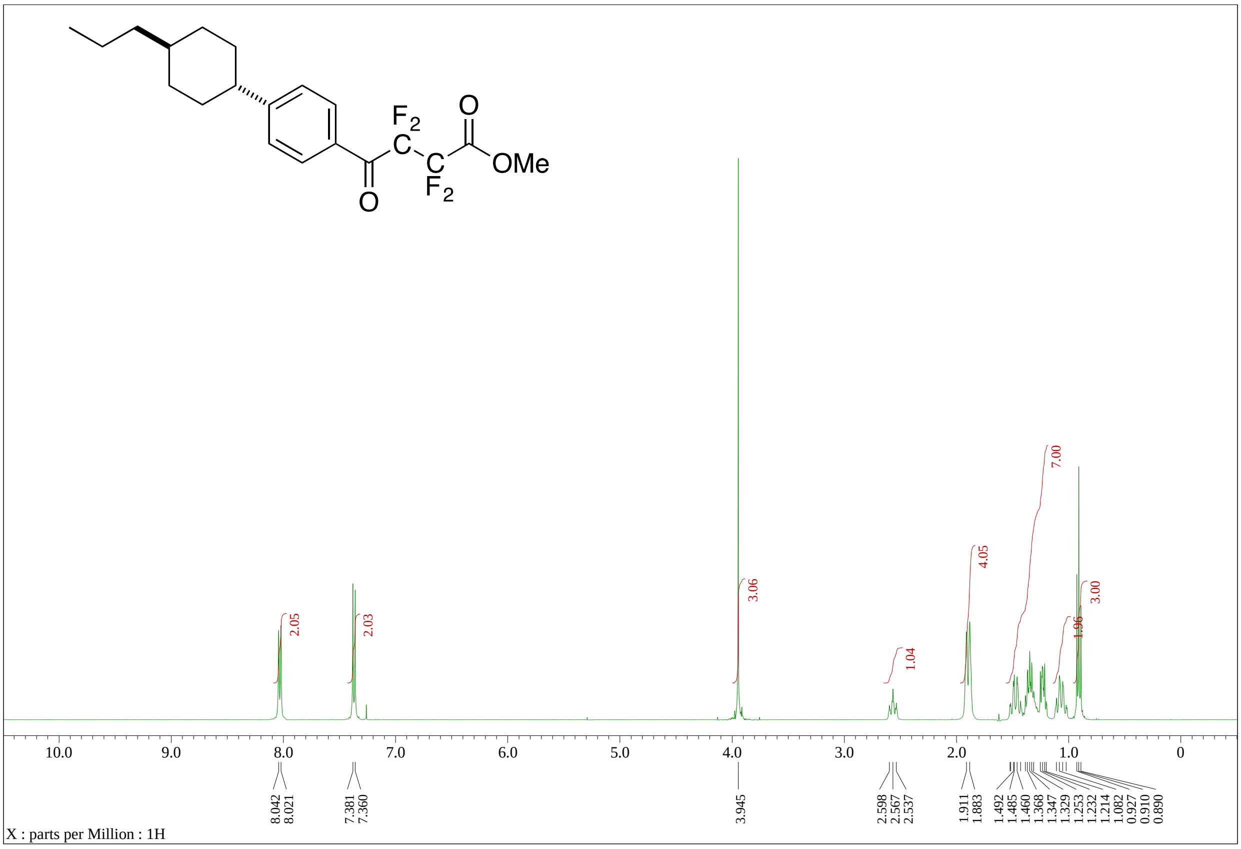

*^{13}C NMR spectrum of methyl 2,2,3,3-tetrafluoro-4-oxo-4-[4-(trans-4-propylcyclohexyl)phenyl]butanoate (**6c**)*

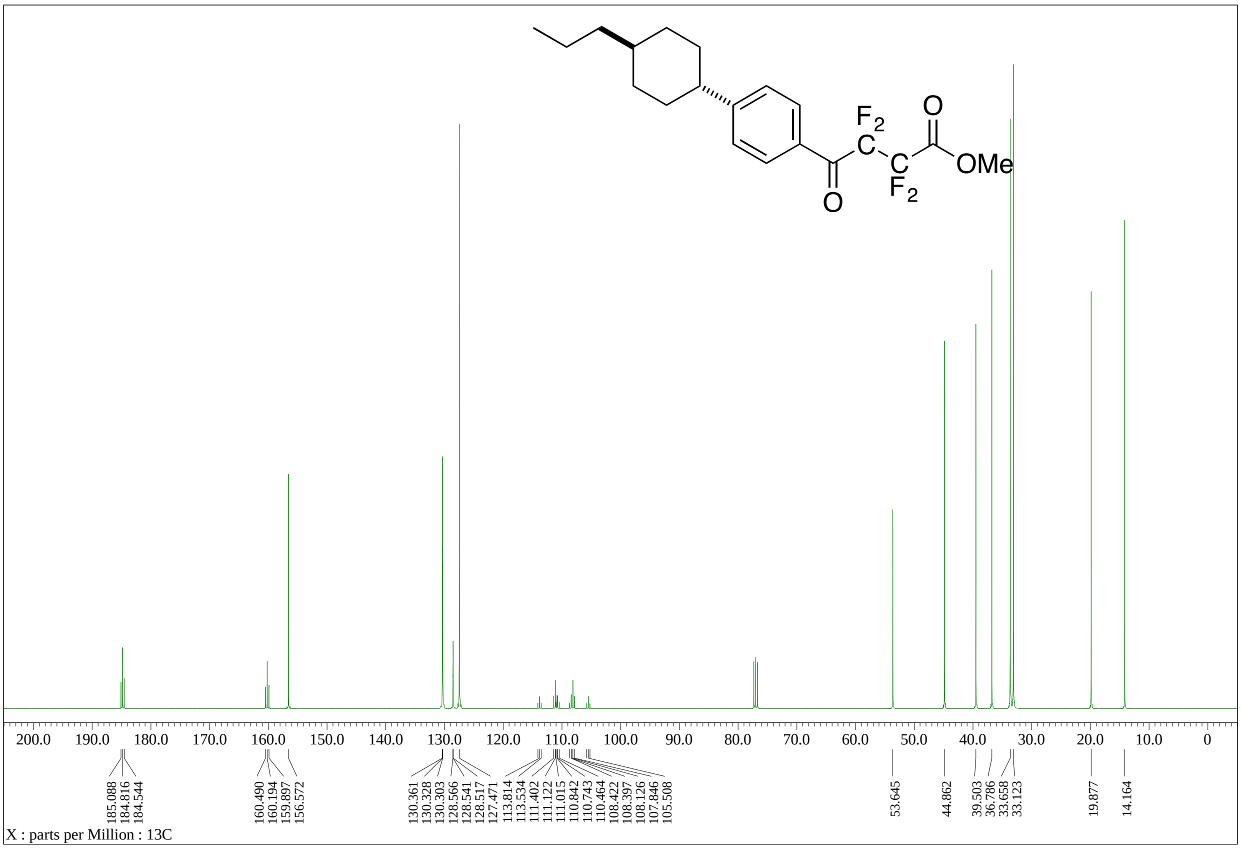

^{19}F NMR spectrum of methyl 2,2,3,3-tetrafluoro-4-oxo-4-[4-(trans-4-propylcyclohexyl)phenyl]butanoate (**6c**)

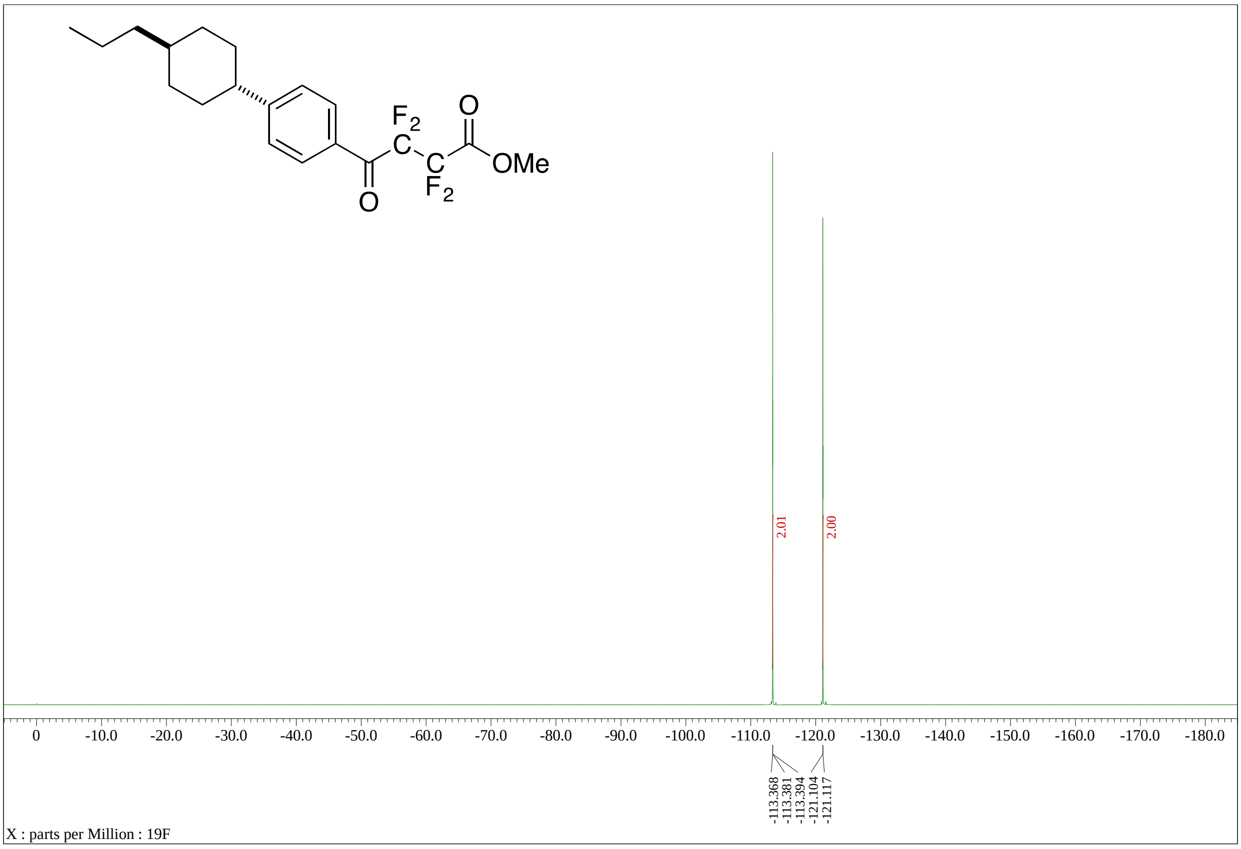

^{1}*H NMR spectrum of 3-Ethenyl-4,4,5,5-tetrafluoro-6-(4-propylphenyl)-1,7-octadiene-3,6-diol (**5a**)*

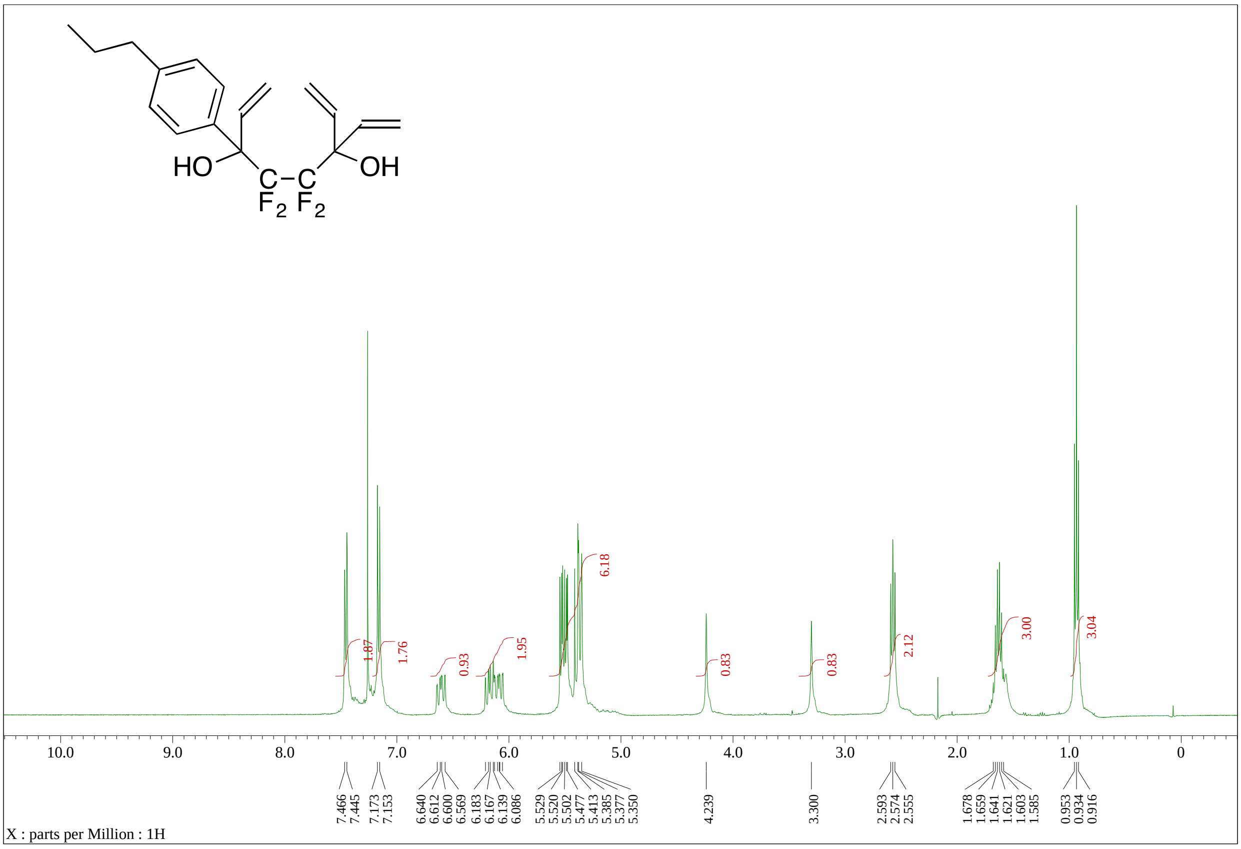

^{13}*C NMR spectrum of 3-Ethenyl-4,4,5,5-tetrafluoro-6-(4-propylphenyl)-1,7-octadiene-3,6-diol (**5a**)*

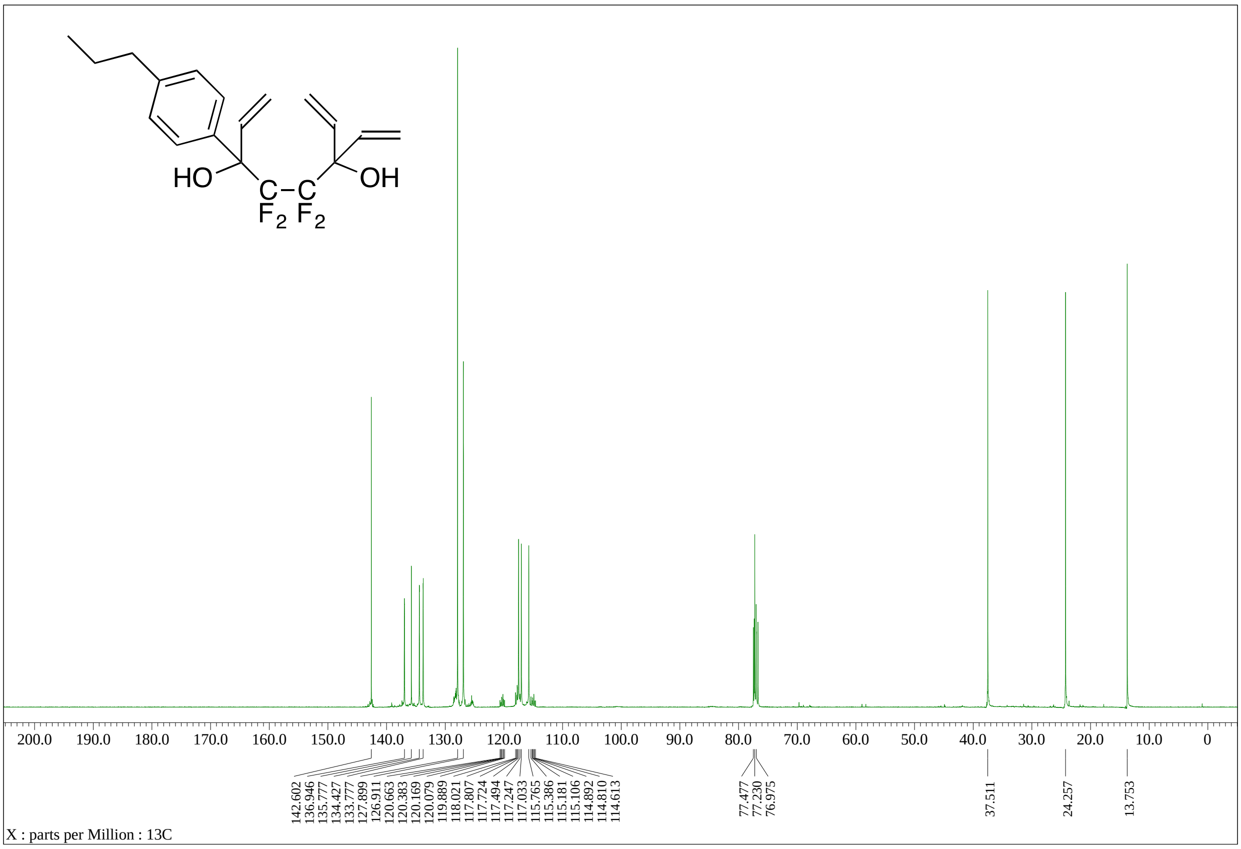

^{19}*F NMR spectrum of 3-Ethenyl-4,4,5,5-tetrafluoro-6-(4-propylphenyl)-1,7-octadiene-3,6-diol* (***5a***)

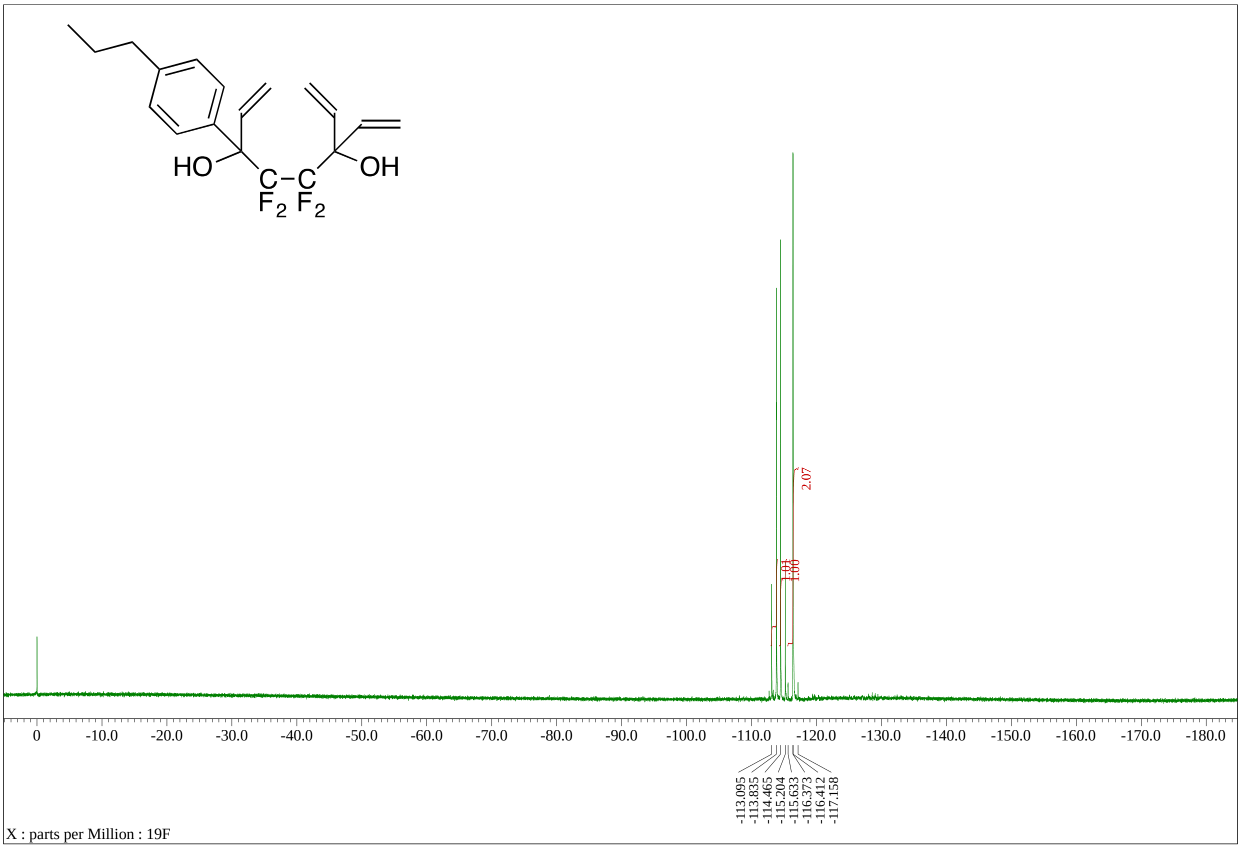

^{1}H NMR spectrum of 2-(3-Buten-1-yl)-5-ethenyl-4,4,5,5-tetrafluoro-5-(4-propylphenyl)tetrahydroduran-2-ol (**8a**)

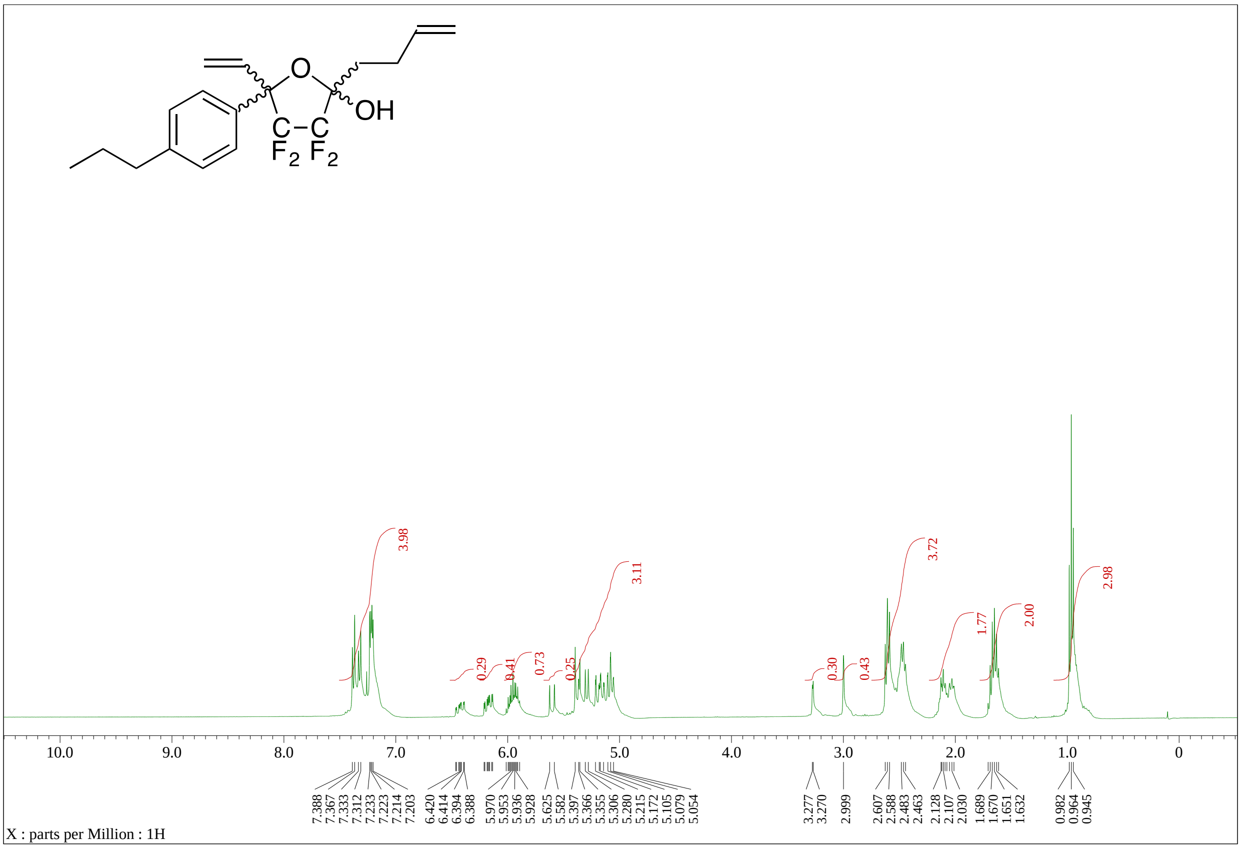

^{13}C NMR spectrum of 2-(3-Buten-1-yl)-5-ethenyl-4,4,5,5-tetrafluoro-5-(4-propylphenyl)tetrahydroduran-2-ol (**8a**)

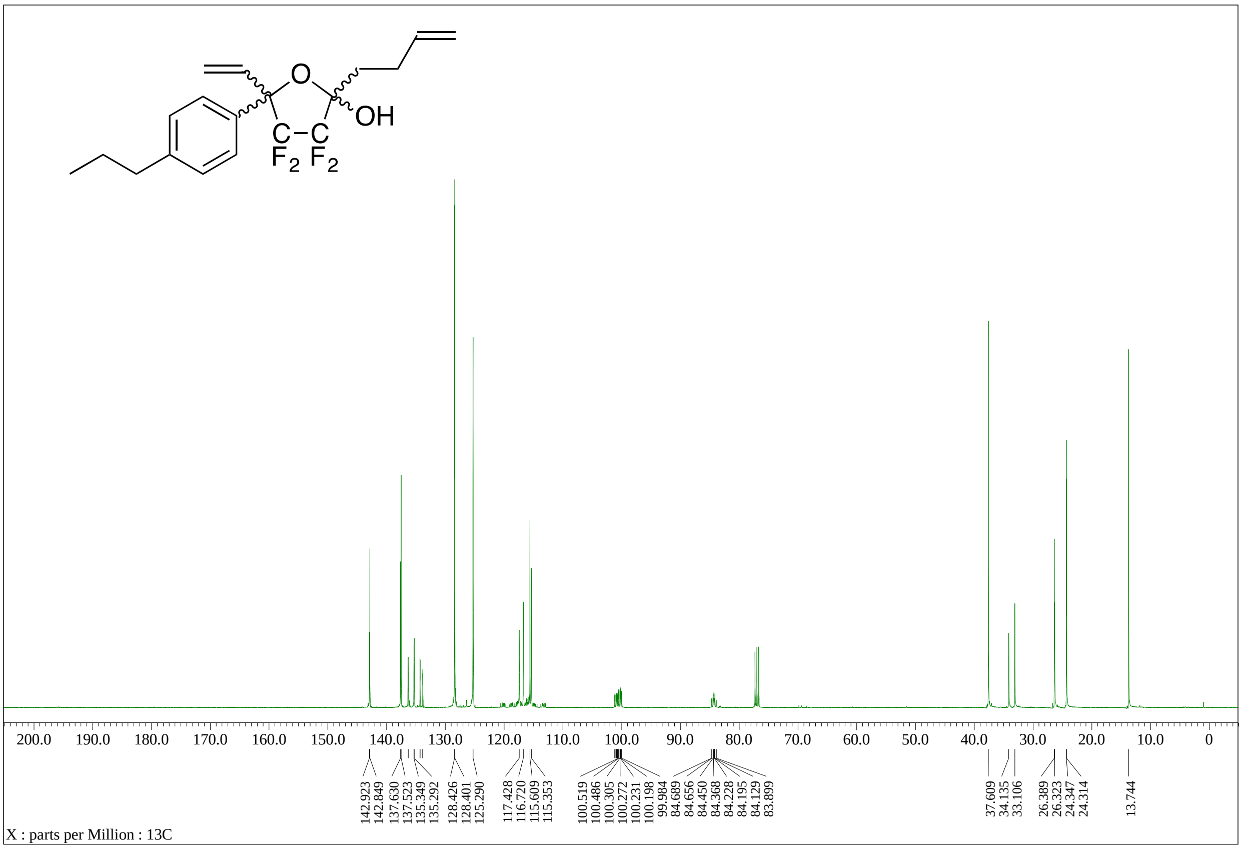

^{19}F NMR spectrum of 2-(3-Buten-1-yl)-5-ethenyl-4,4,5,5-tetrafluoro-5-(4-propylphenyl)tetrahydroduran-2-ol (**8a**)

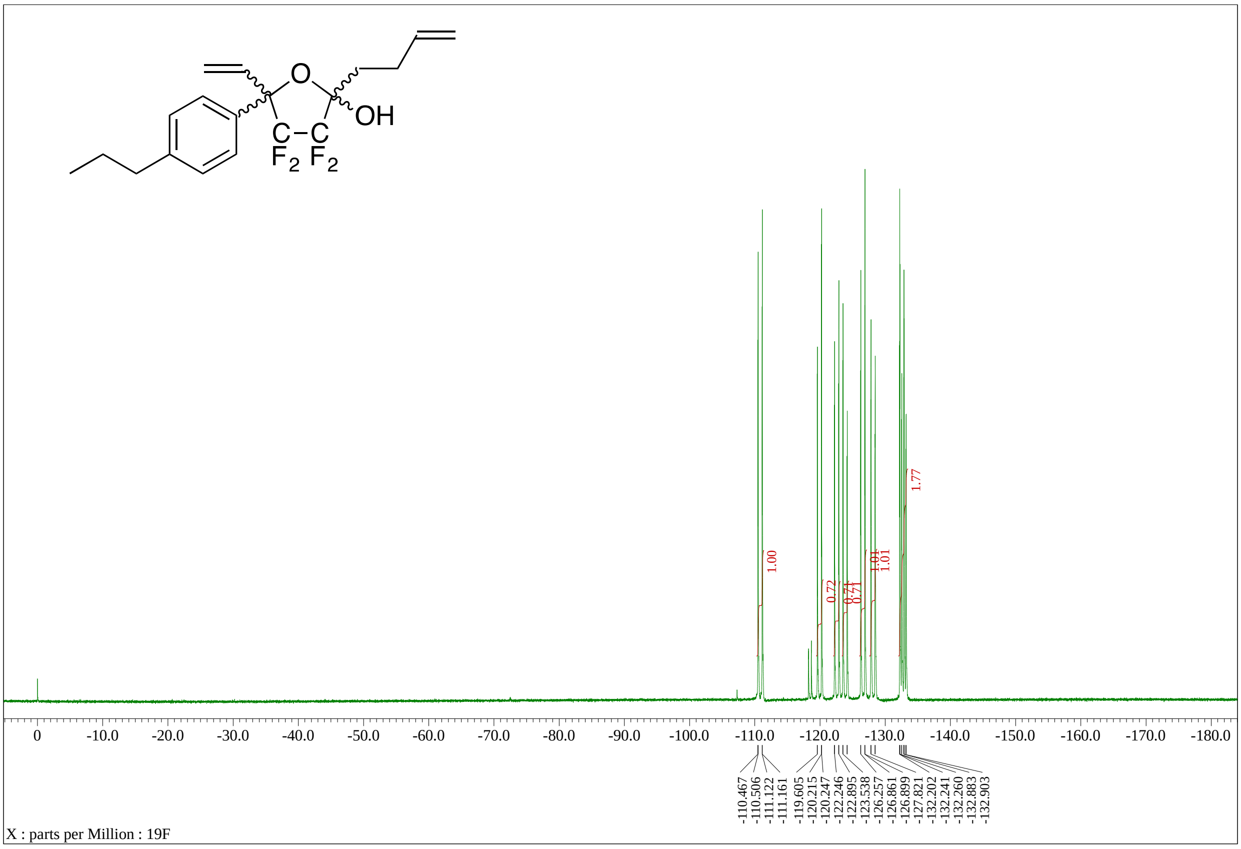

*^{1}H NMR spectrum of 3-Ethenyl-4,4,5,5-tetrafluoro-6-[4-(4-propylphenyl)phenyl]-1,7-octadiene-3,6-diol (**5b**)*

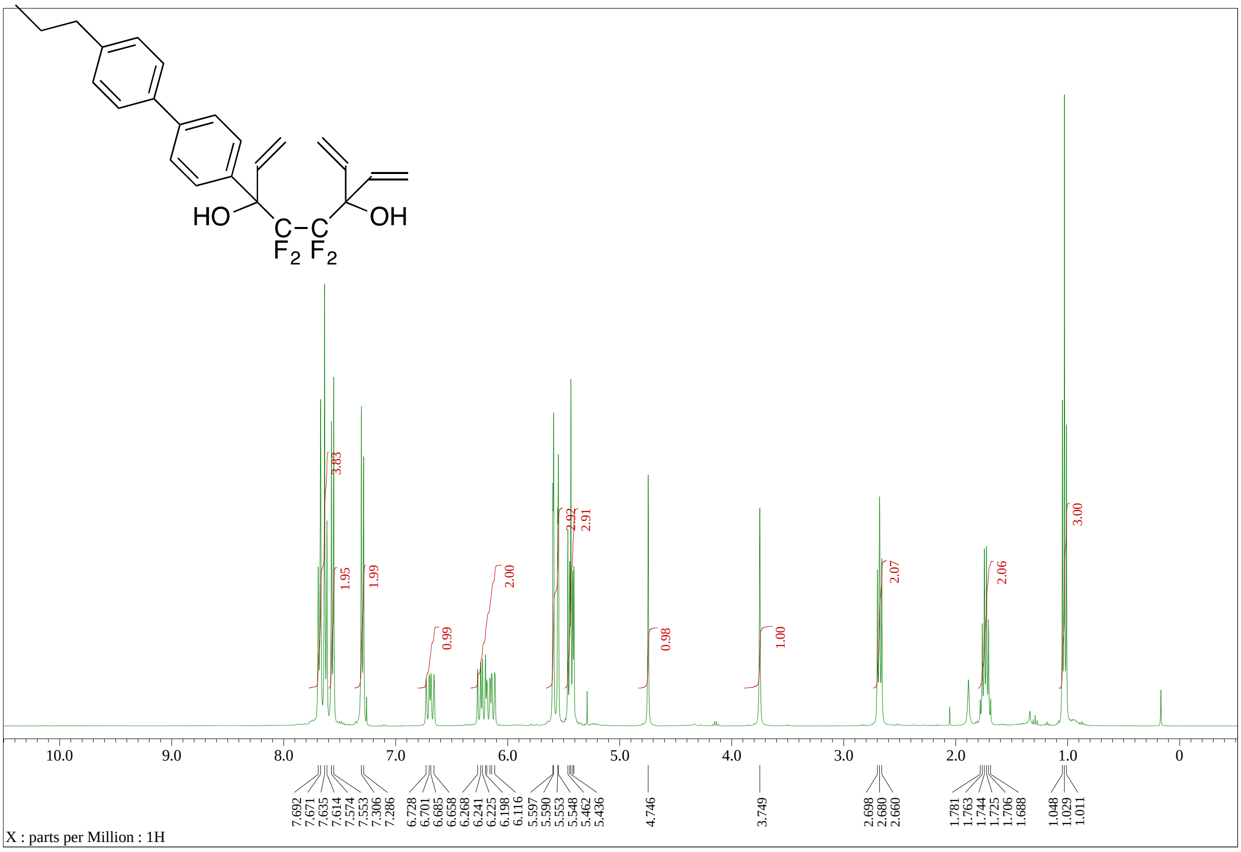

*^{13}C NMR spectrum of 3-Ethenyl-4,4,5,5-tetrafluoro-6-[4-(4-propylphenyl)phenyl]-1,7-octadiene-3,6-diol (**5b**)*

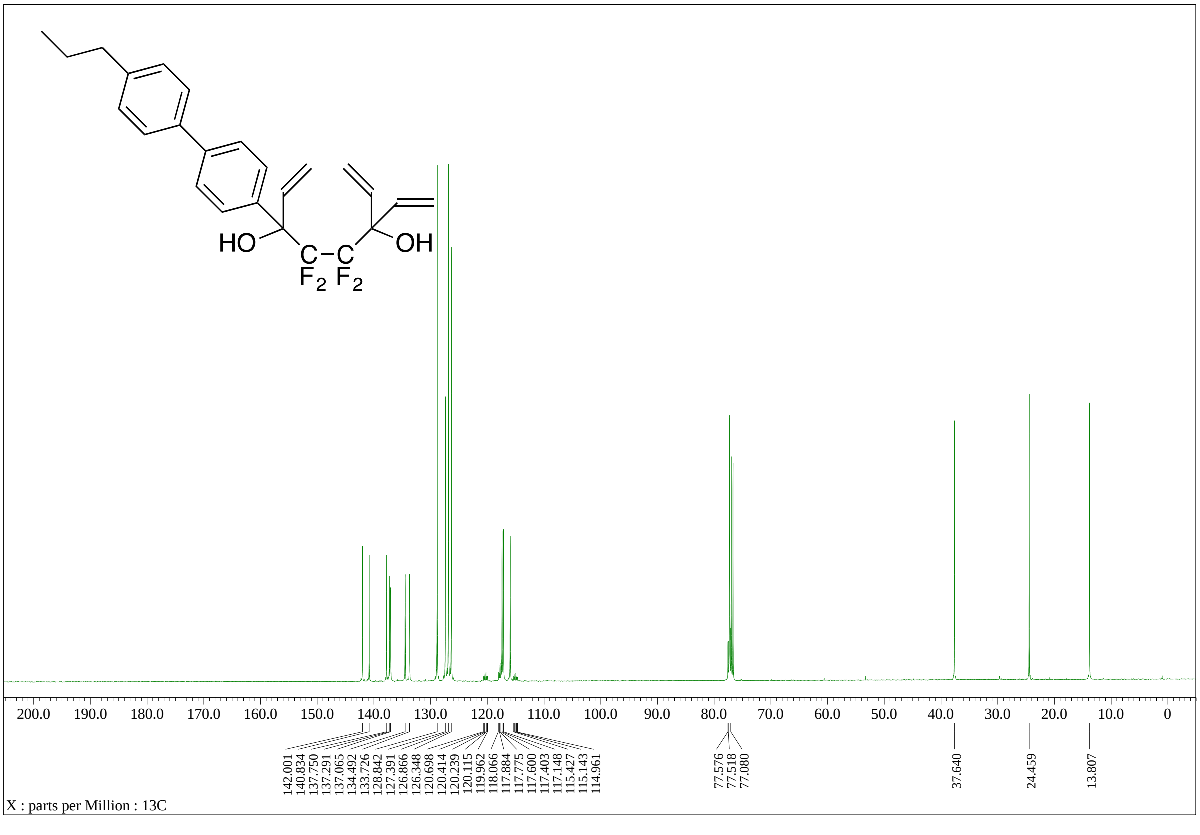

*^{19}F NMR spectrum of 3-Ethenyl-4,4,5,5-tetrafluoro-6-[4-(4-propylphenyl)phenyl]-1,7-octadiene-3,6-diol (**5b**)*

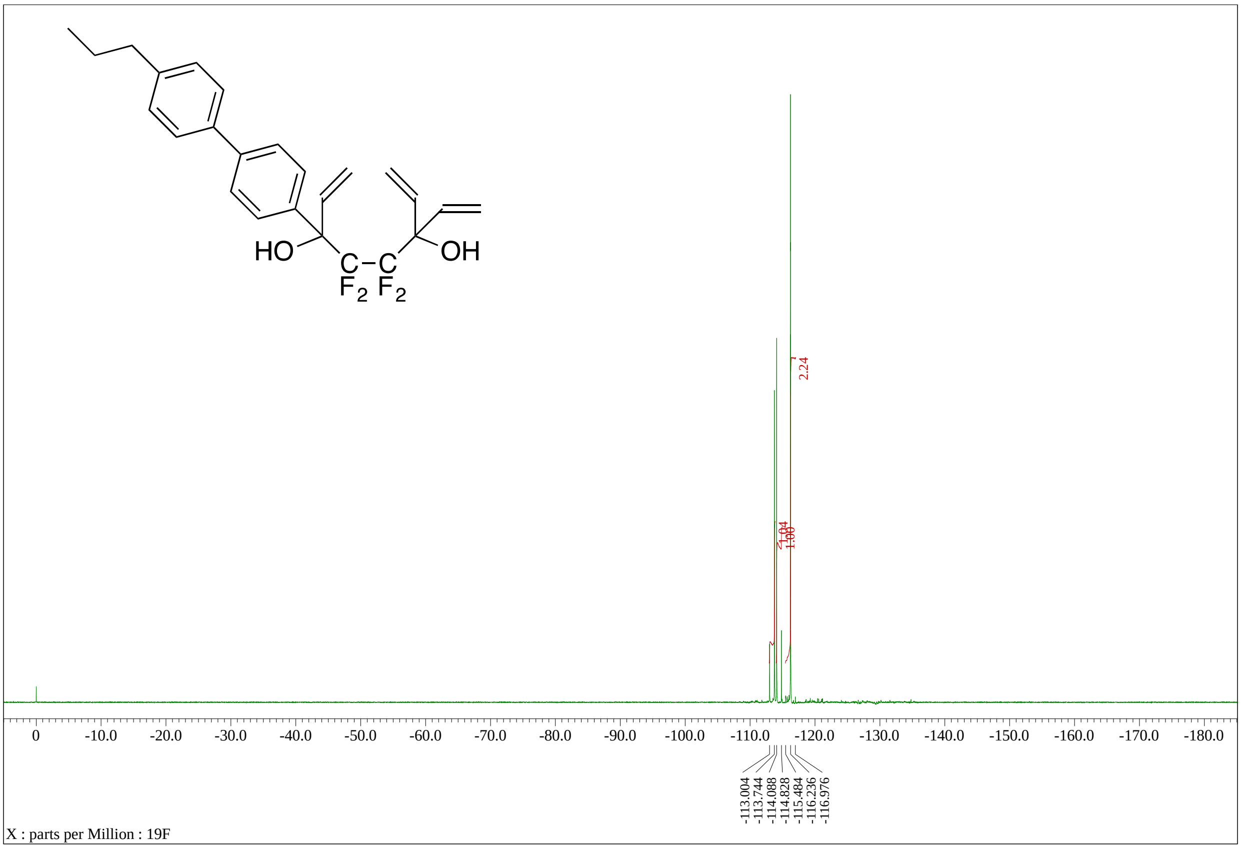

^{1}H NMR spectrum of 2-(3-Buten-1-yl)-5-ethenyl-4,4,5,5-tetrafluoro-5-[4-(4-propylphenyl)phenyl] tetrahydroduran-2-ol ***(8b)***

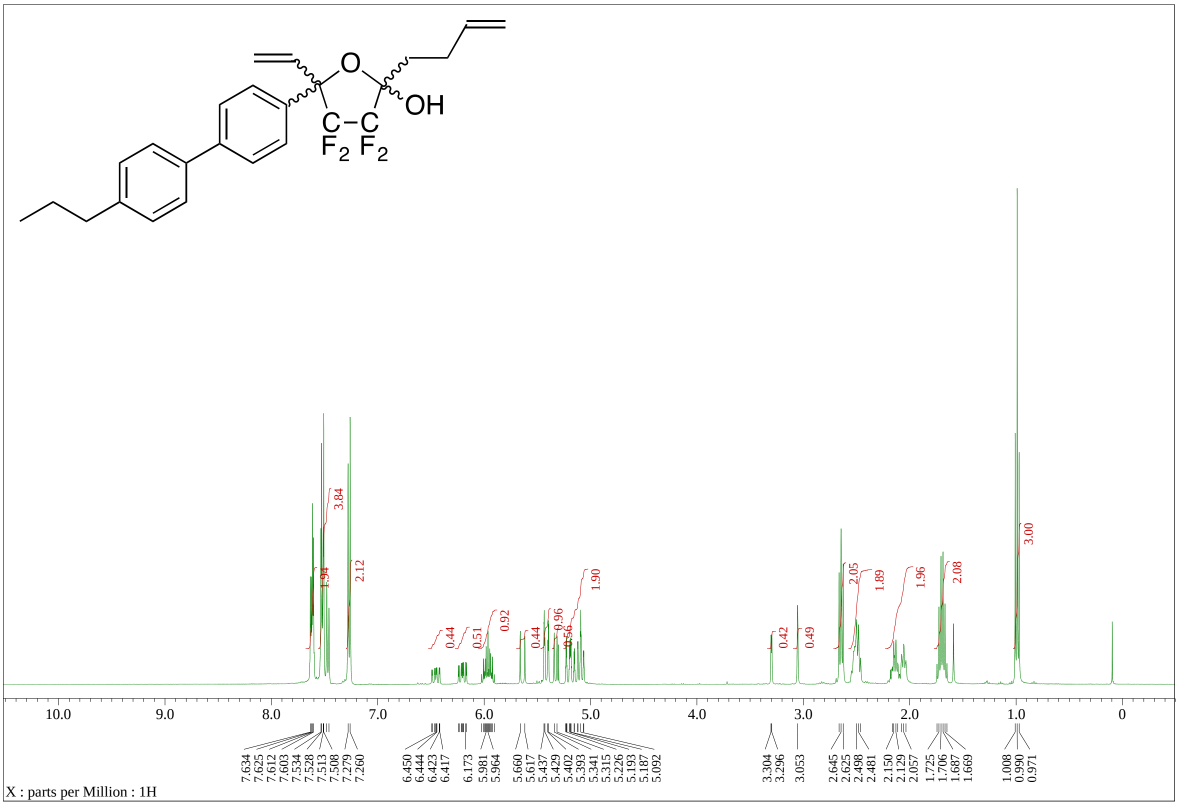

^{13}C NMR spectrum of 2-(3-Buten-1-yl)-5-ethenyl-4,4,5,5-tetrafluoro-5-[4-(4-propylphenyl)phenyl] tetrahydroduran-2-ol ***(8b)***

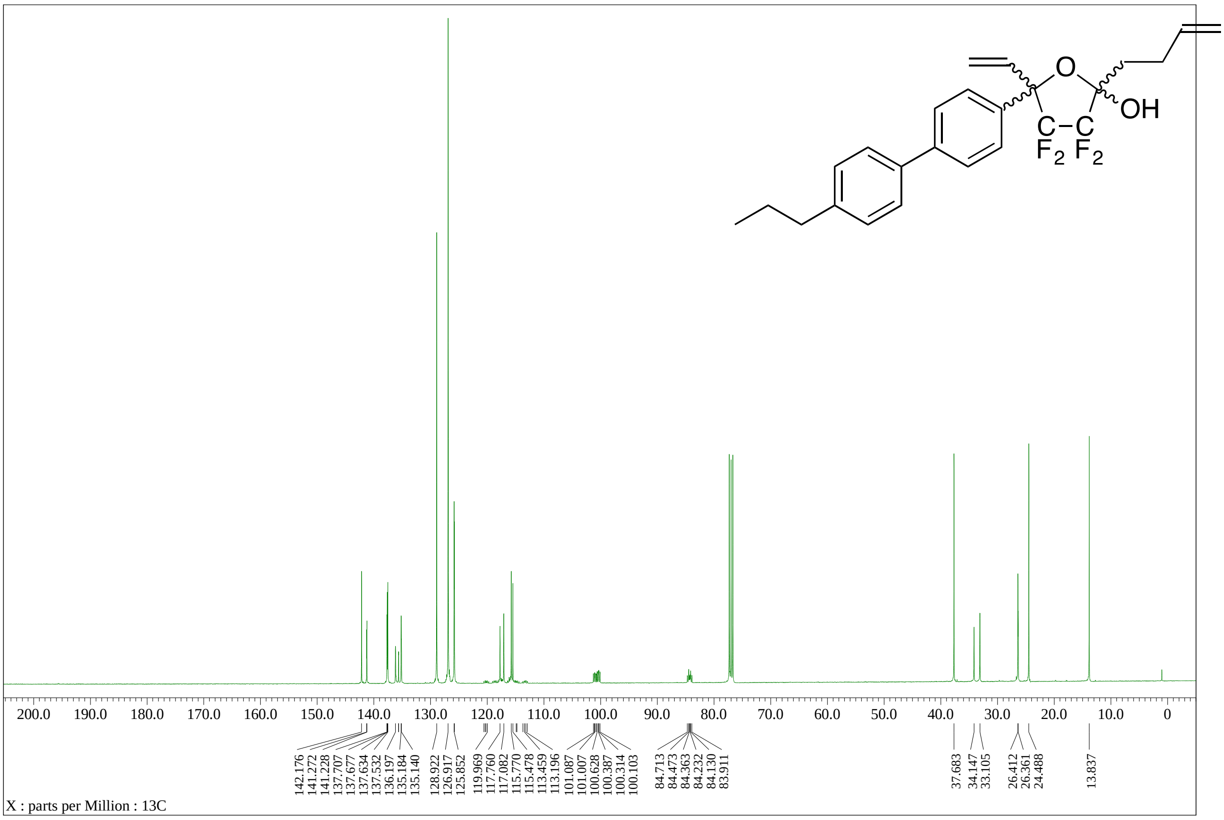

*^{19}F NMR spectrum of 2-(3-Buten-1-yl)-5-ethenyl-4,4,5,5-tetrafluoro-5-[4-(4-propylphenyl)phenyl] tetrahydroduran-2-ol (**8b**)*

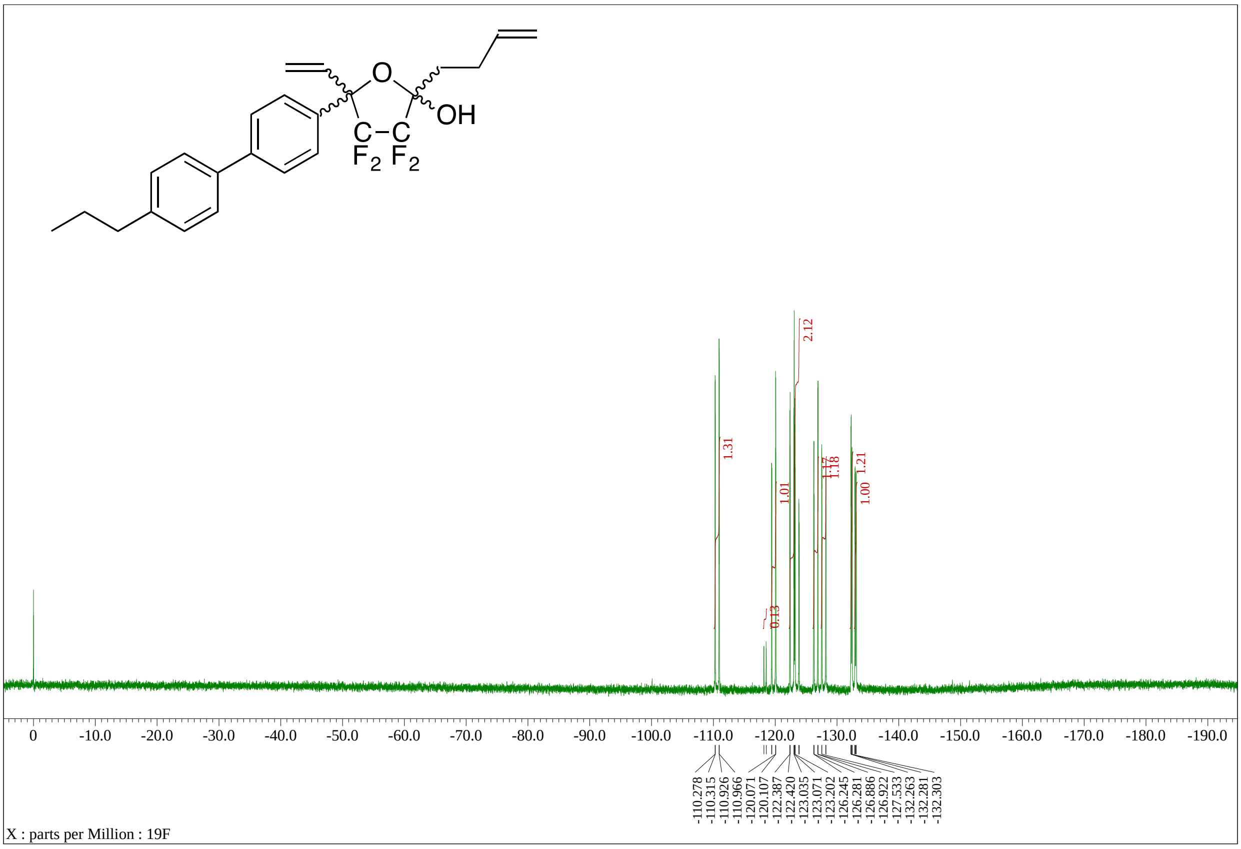

*^{1}H NMR spectrum of 3-Ethenyl-4,4,5,5-tetrafluoro-6-[4-(trans-4-propylcyclohexyl)phenyl]-1,7-octadiene -3,6-diol (**5c**)*

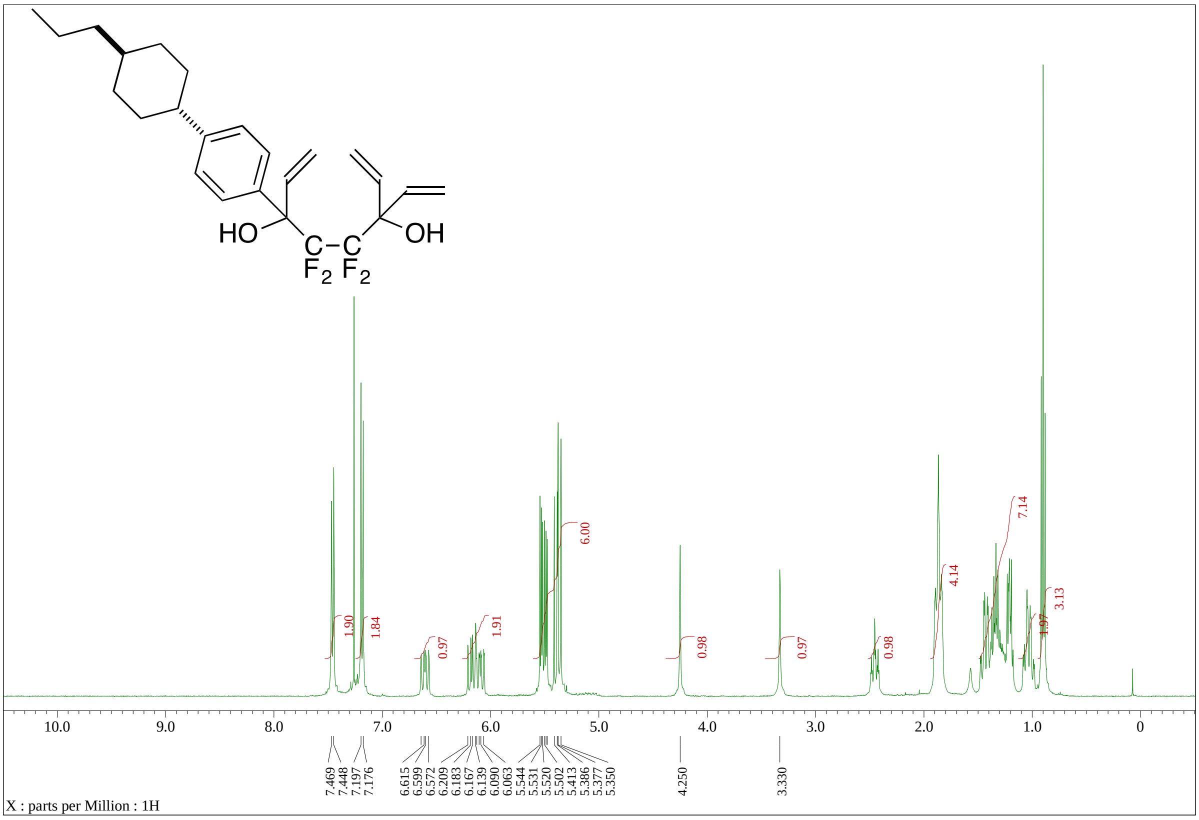

*^{13}C NMR spectrum of 3-Ethenyl-4,4,5,5-tetrafluoro-6-[4-(trans-4-propylcyclohexyl)phenyl]-1,7-octadiene -3,6-diol (**5c**)*

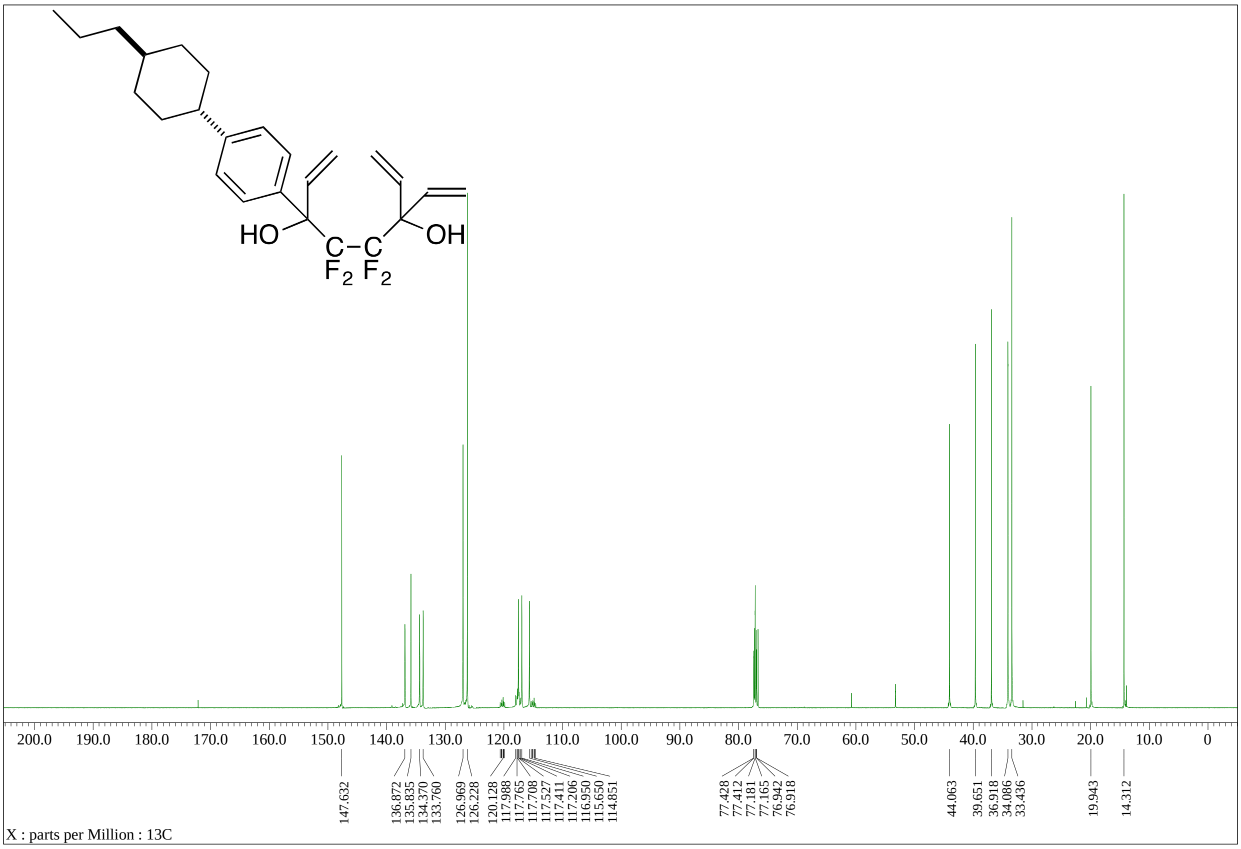

^{19}F NMR spectrum of 3-Ethenyl-4,4,5,5-tetrafluoro-6-[4-(trans-4-propylcyclohexyl)phenyl]-1,7-octadiene -3,6-diol ***(5c)***

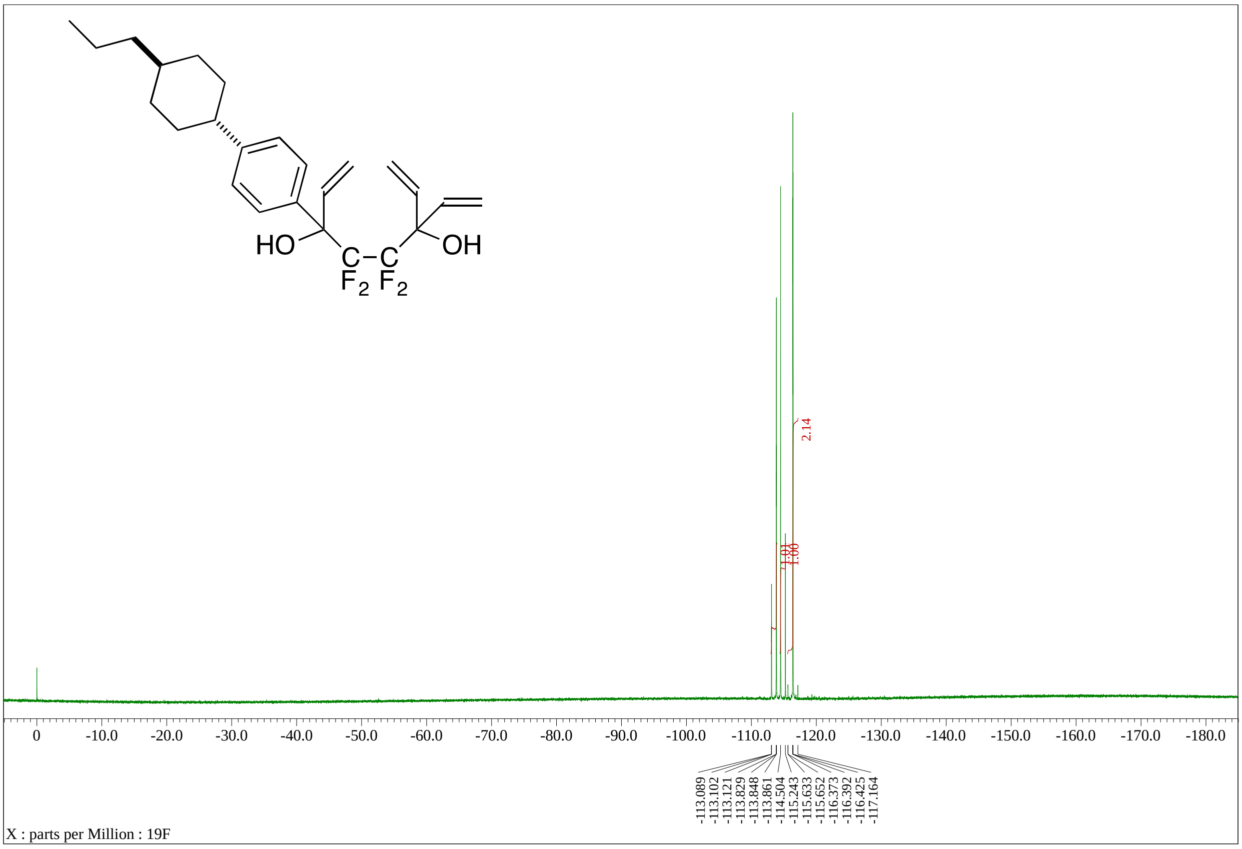

^{1}H NMR spectrum of 2-(3-Buten-1-yl)-5-ethenyl-4,4,5,5-tetrafluoro-5-[4-(trans-4-propylcyclohexyl)phenyl] tetrahydrofuran-2-ol (**8c**)

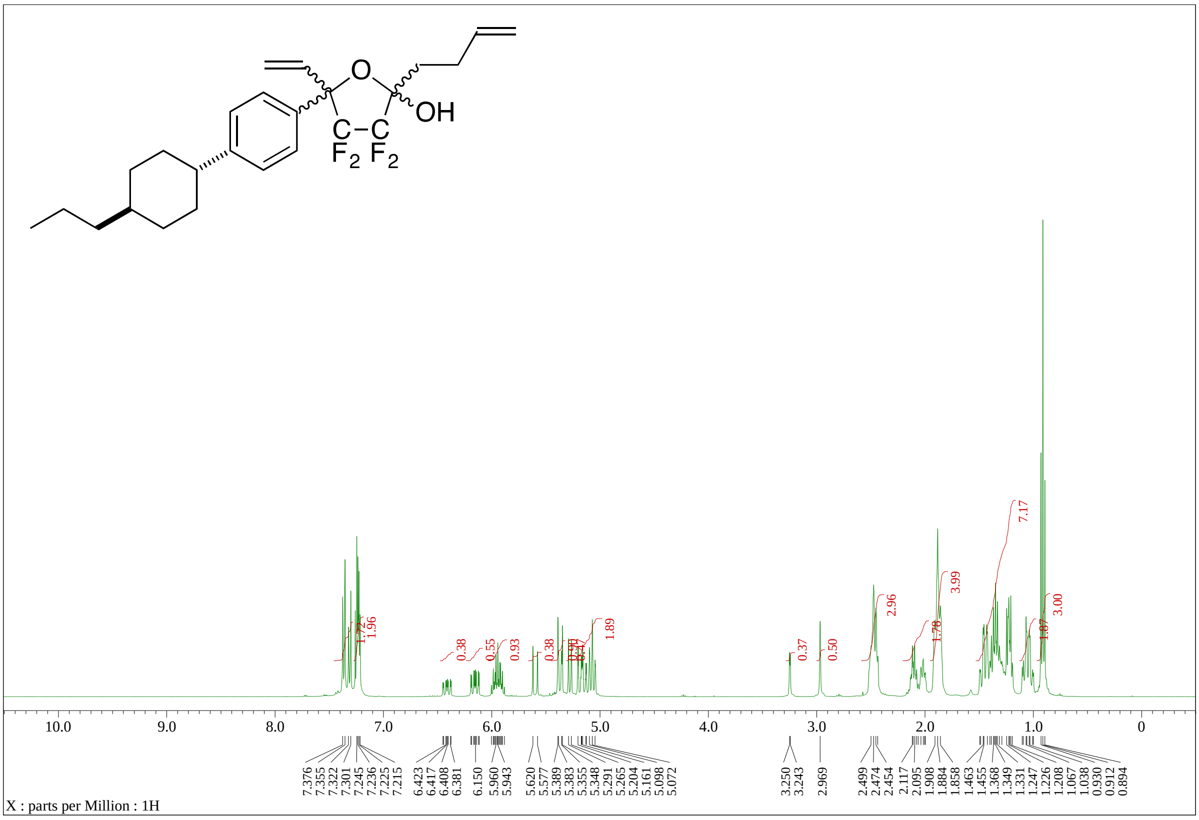

^{13}C NMR spectrum of 2-(3-Buten-1-yl)-5-ethenyl-4,4,5,5-tetrafluoro-5-[4-(trans-4-propylcyclohexyl)phenyl] tetrahydrofuran-2-ol (**8c**)

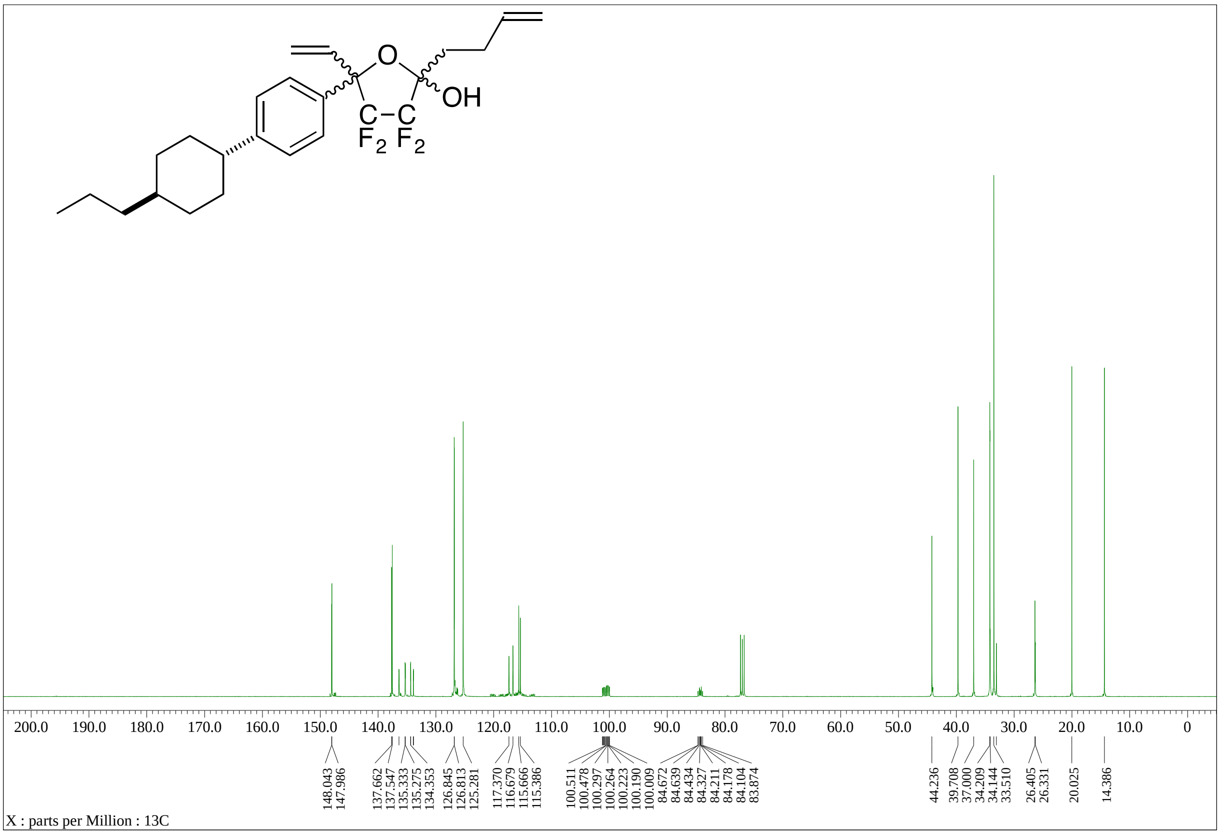

*^{19}F NMR spectrum of 2-(3-Buten-1-yl)-5-ethenyl-4,4,5,5-tetrafluoro-5-[4-(trans-4-propylcyclohexyl)phenyl] tetrahydrofuran-2-ol (**8c**)*

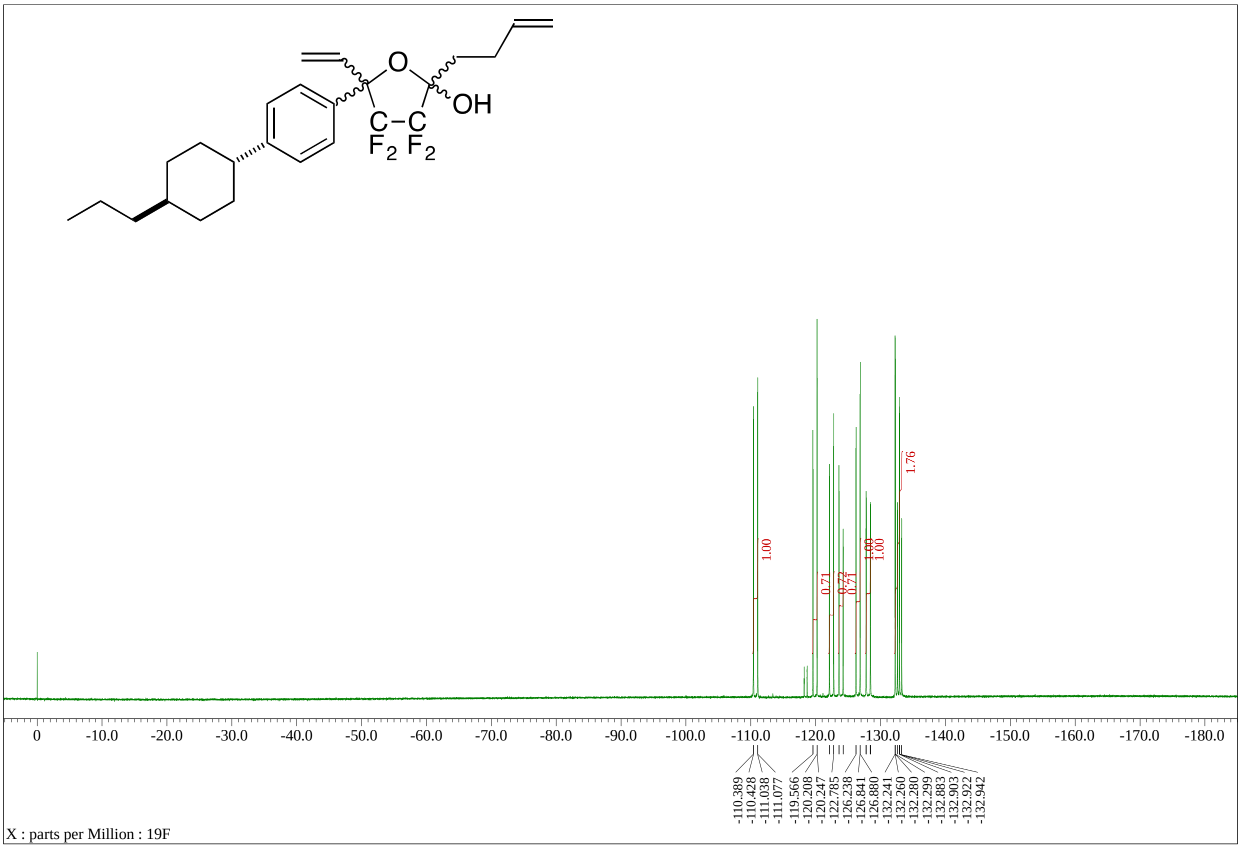

^{1}H NMR spectrum of 1-Ethenyl-5,5,6,6-tetrafluoro-4-(4-propylphenyl)-2-cyclohexene-1,4-diol (**4a**)

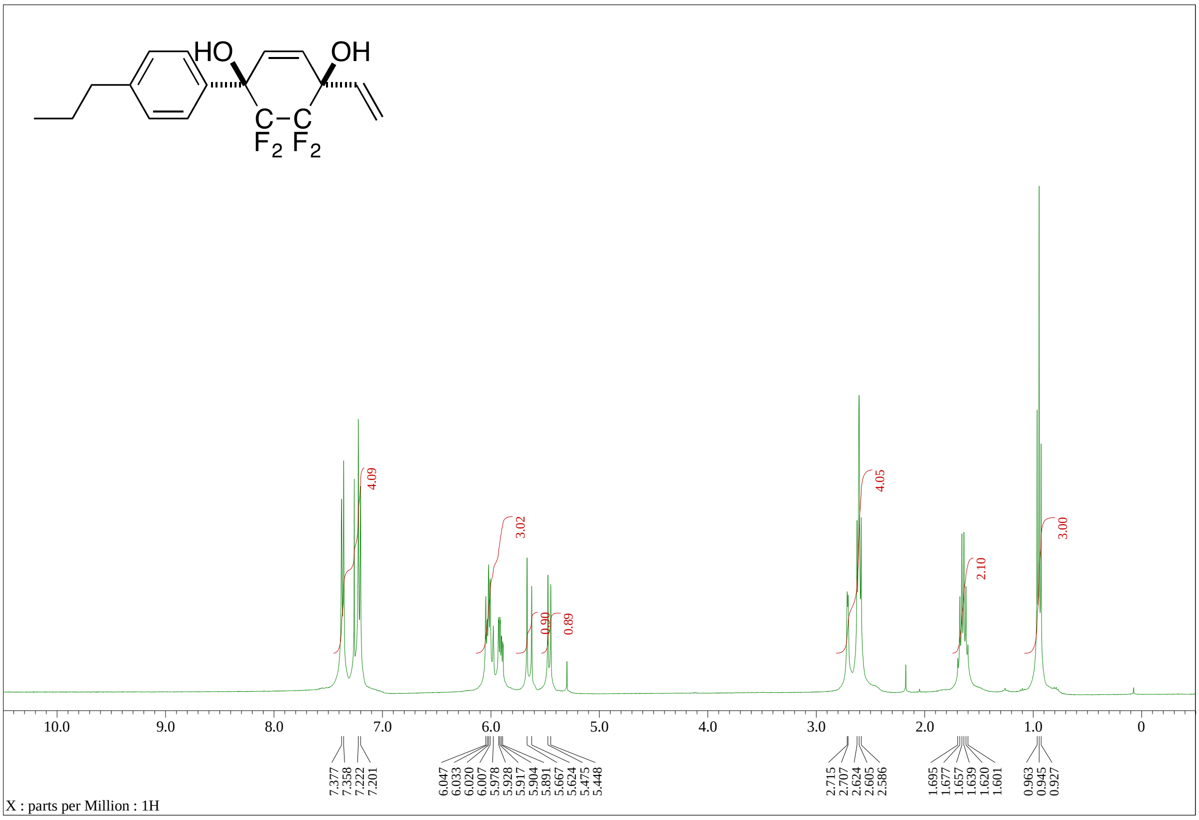

^{13}C NMR spectrum of 1-Ethenyl-5,5,6,6-tetrafluoro-4-(4-propylphenyl)-2-cyclohexene-1,4-diol (**4a**)

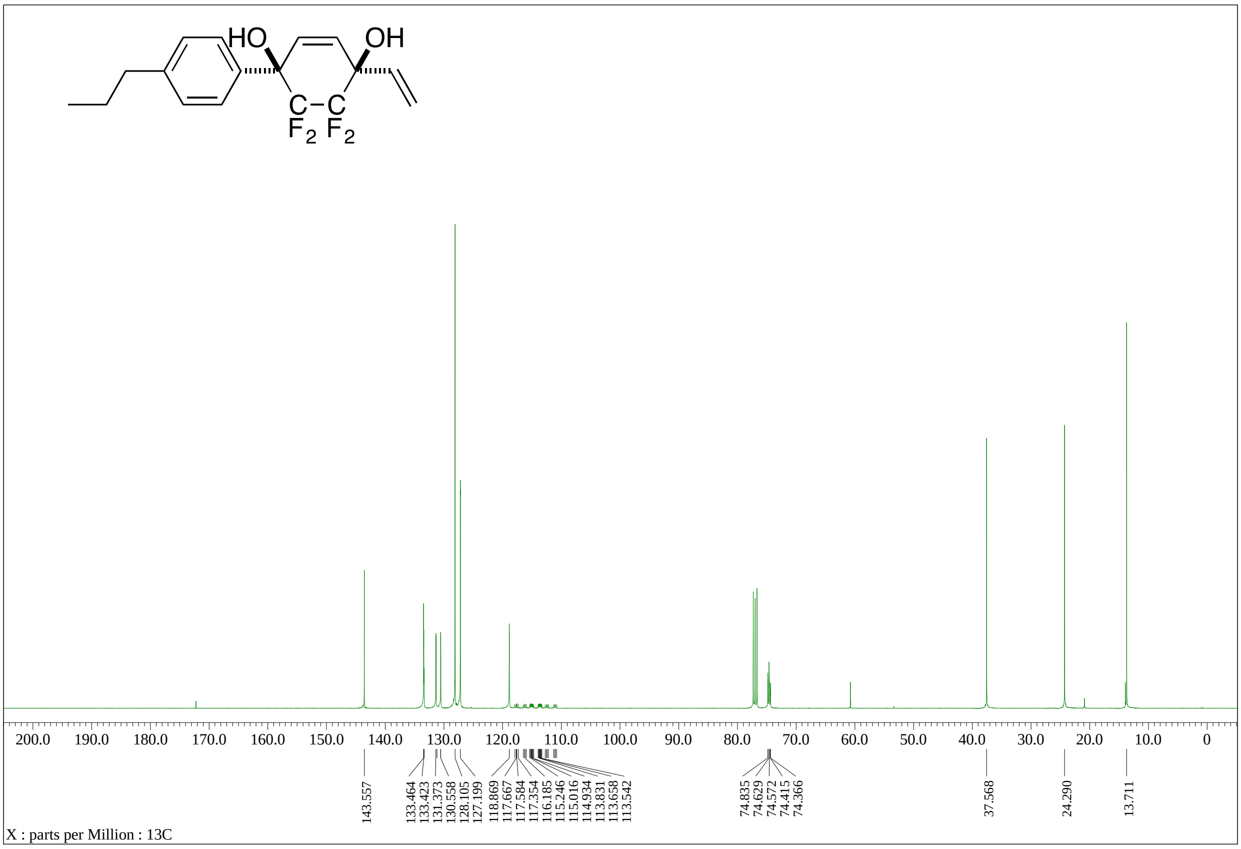

*^{19}F NMR spectrum of 1-Ethenyl-5,5,6,6-tetrafluoro-4-(4-propylphenyl)-2-cyclohexene-1,4-diol (**4a**)*

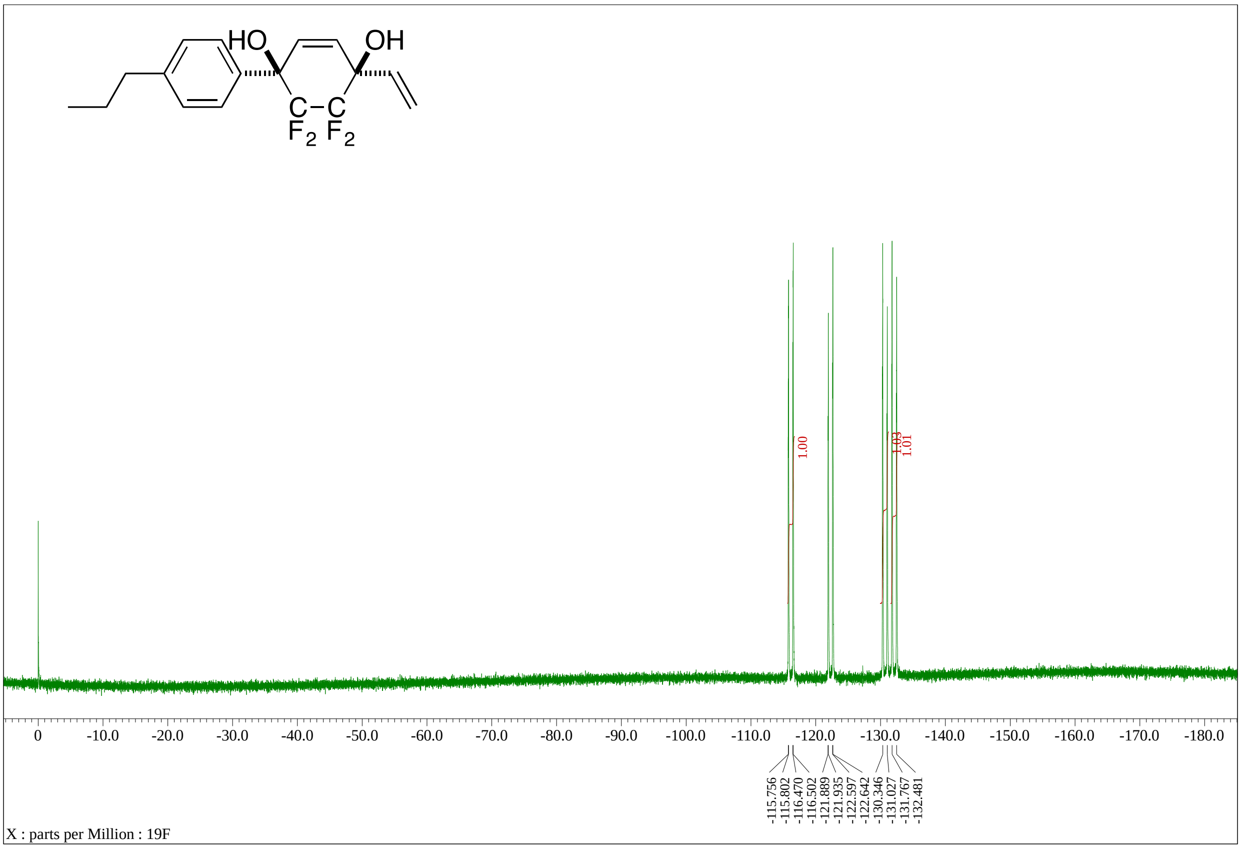

^{1}H NMR spectrum of 1-Ethenyl-5,5,6,6-tetrafluoro-4-[4-(4-propylphenyl)phenyl]-2-cyclohexene-1,4-diol (**4b**)

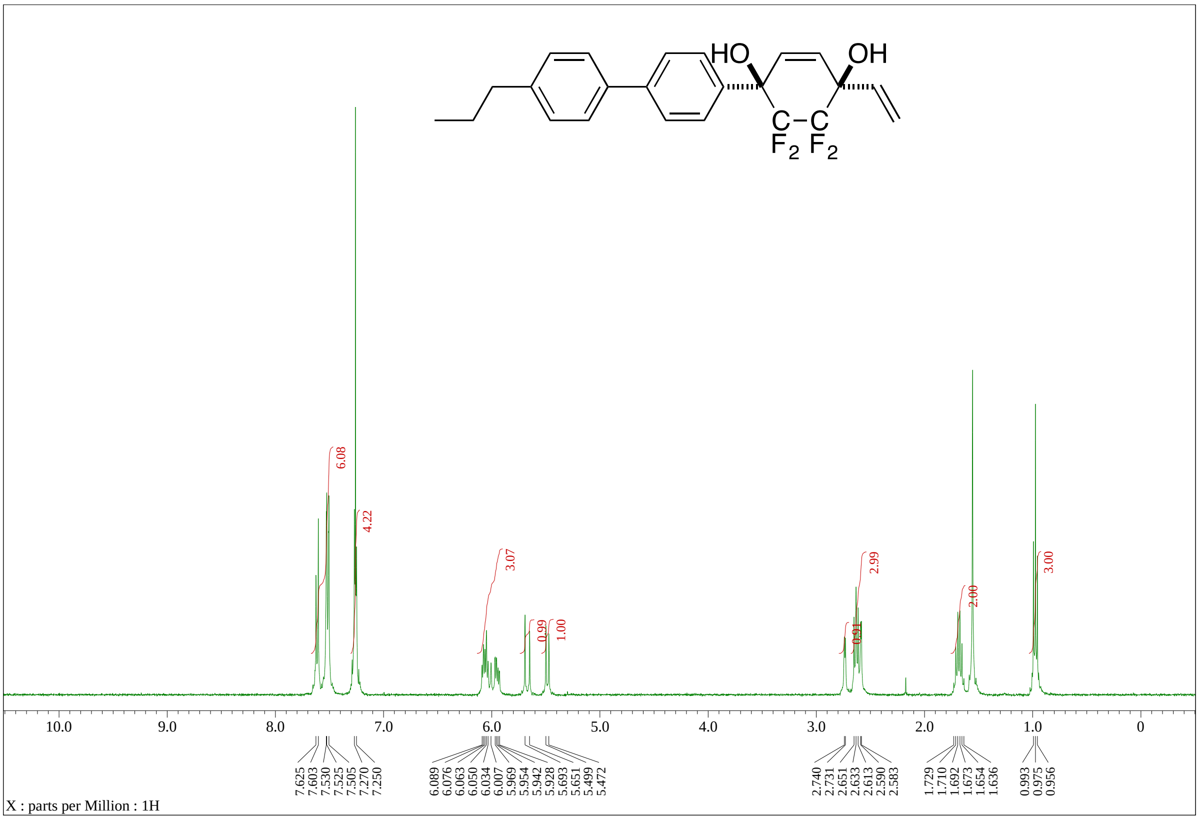

^{13}C NMR spectrum of 1-Ethenyl-5,5,6,6-tetrafluoro-4-[4-(4-propylphenyl)phenyl]-2-cyclohexene-1,4-diol (**4b**)

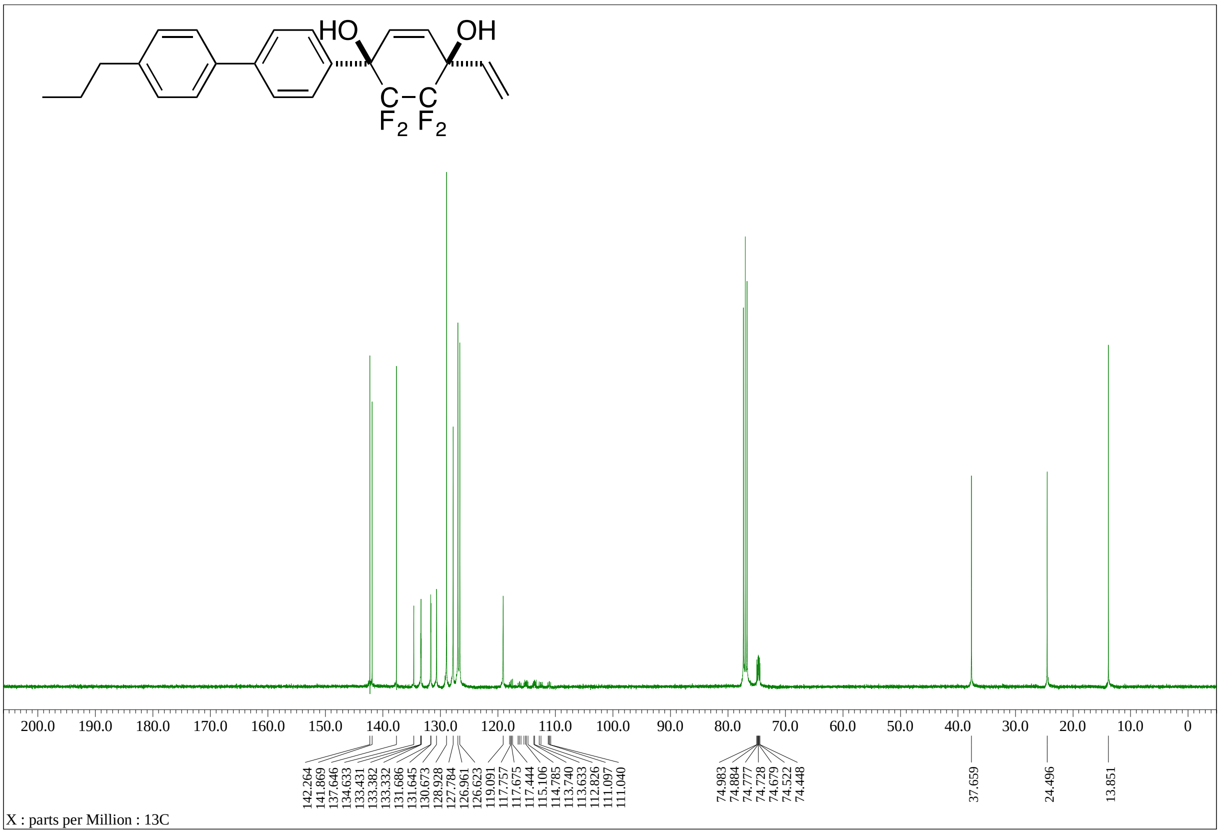

*^{19}F NMR spectrum of 1-Ethenyl-5,5,6,6-tetrafluoro-4-[4-(4-propylphenyl)phenyl]-2-cyclohexene-1,4-diol (**4b**)*

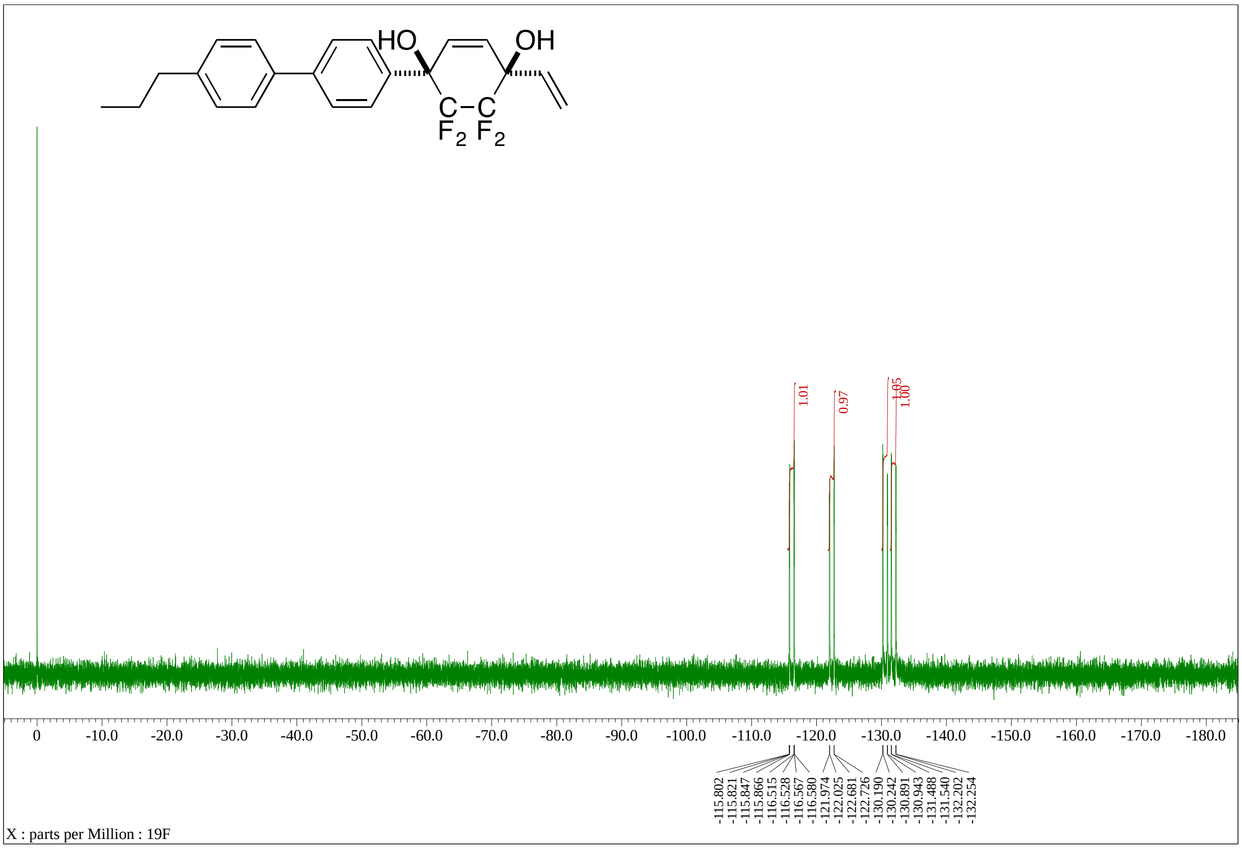

^{1}H NMR spectrum of 1-Ethenyl-5,5,6,6-tetrafluoro-4-[4-(trans-4-propylcyclohexyl)phenyl]-2-cyclohexene -1,4-diol ***(4c)***

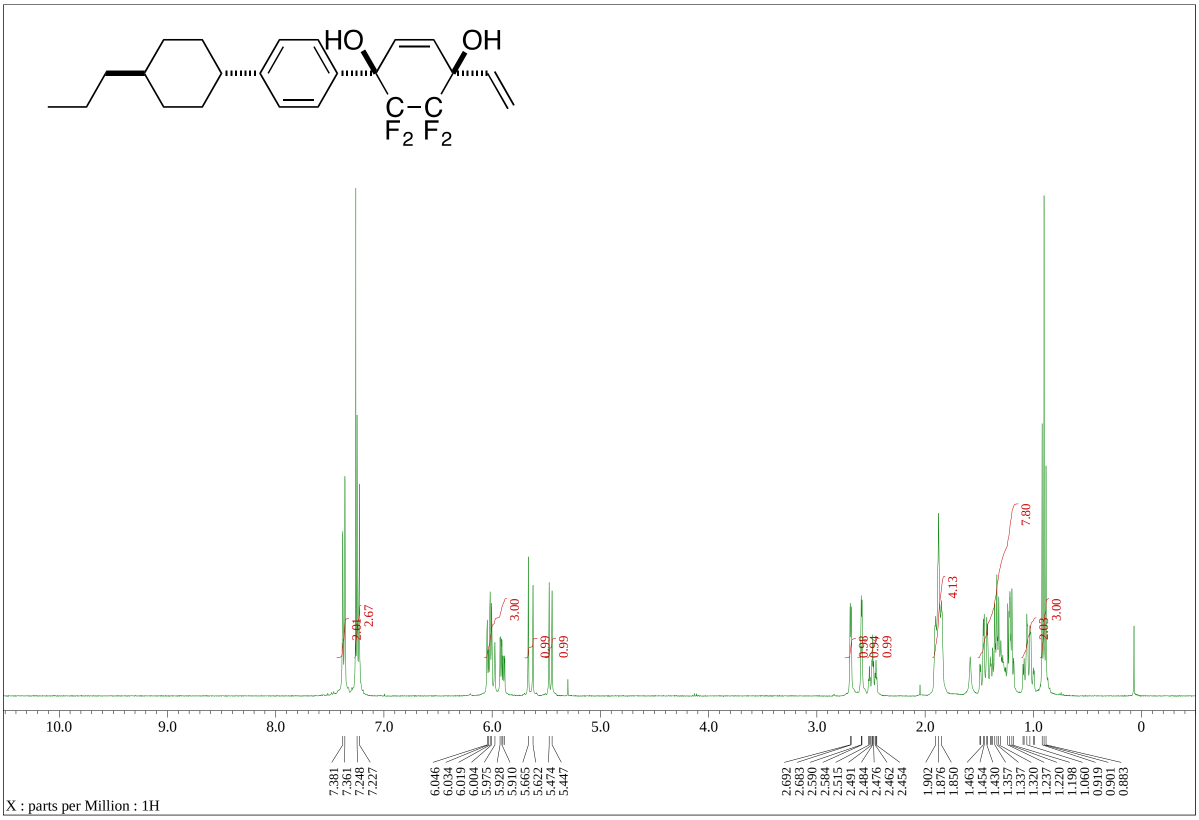

^{13}C NMR spectrum of 1-Ethenyl-5,5,6,6-tetrafluoro-4-[4-(trans-4-propylcyclohexyl)phenyl]-2-cyclohexene -1,4-diol ***(4c)***

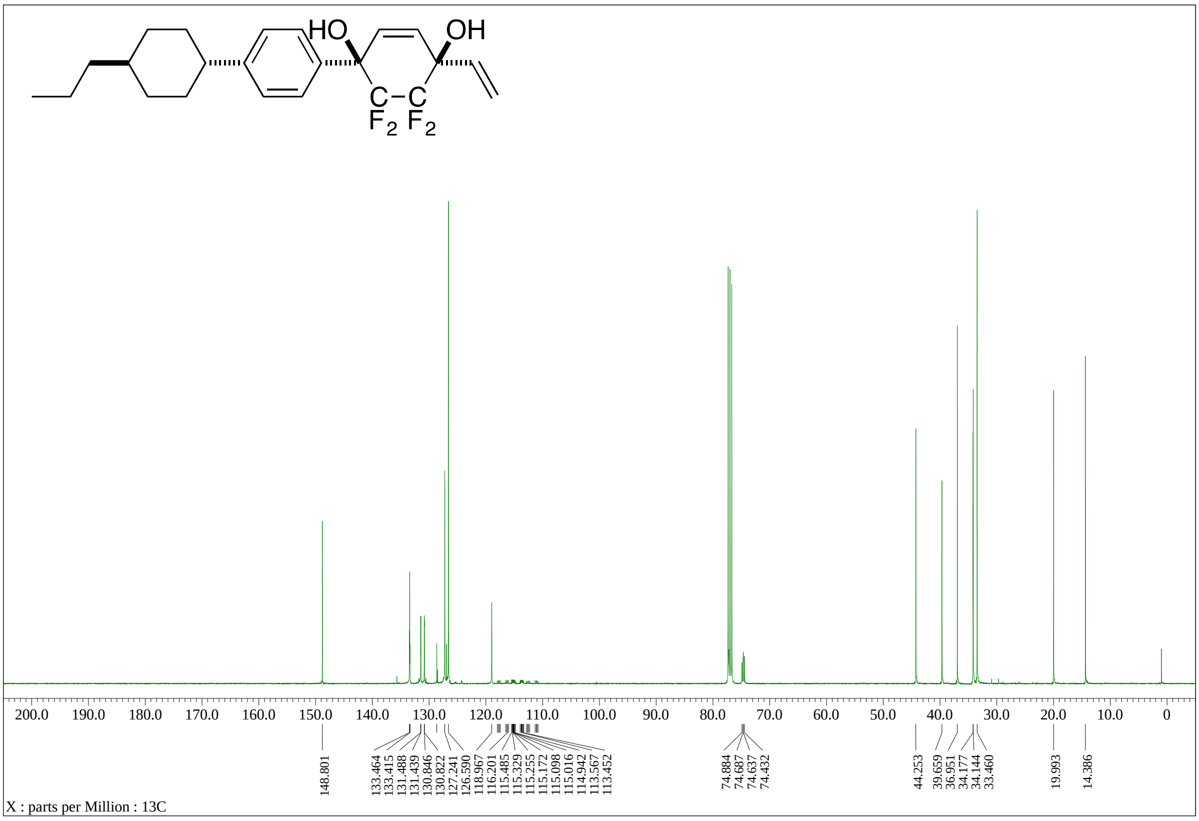

*^{19}F NMR spectrum of 1-Ethenyl-5,5,6,6-tetrafluoro-4-[4-(trans-4-propylcyclohexyl)phenyl]-2-cyclohexene -1,4-diol (**4c**)*

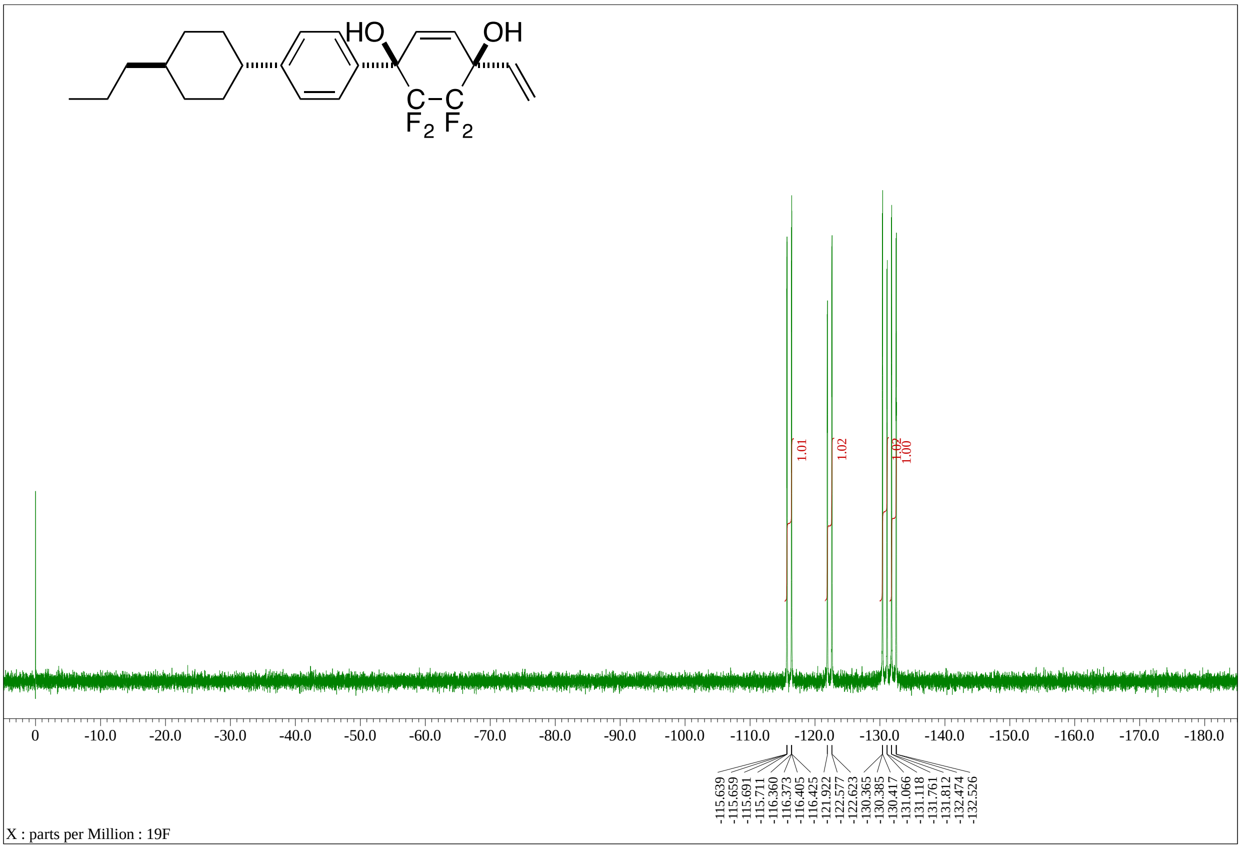

*^{1}H NMR spectrum of cis-1-Ethyl-2,2,3,3-tetrafluoro-4-(4-propylphenyl)cyclohexane-1,4-diol (**3a**)*

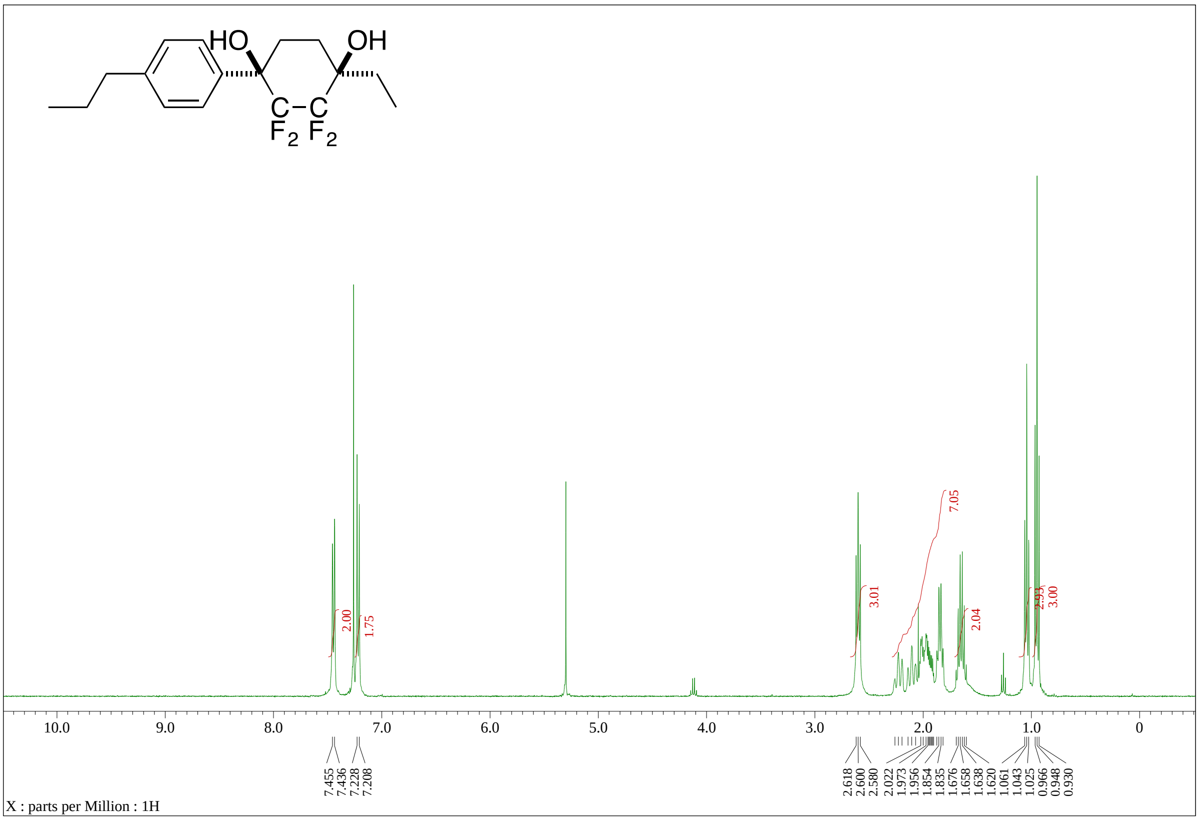

*^{13}C NMR spectrum of cis-1-Ethyl-2,2,3,3-tetrafluoro-4-(4-propylphenyl)cyclohexane-1,4-diol (**3a**)*

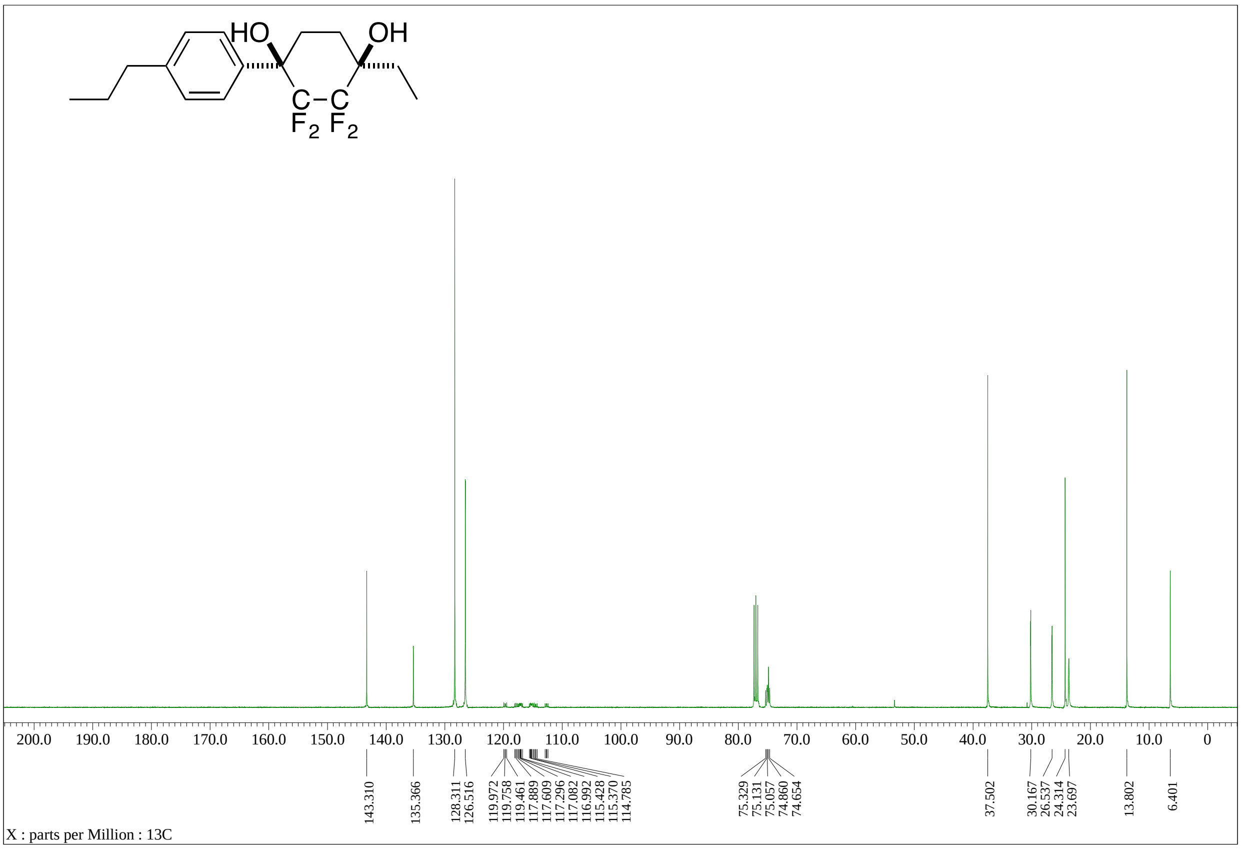

*^{19}F NMR spectrum of cis-1-Ethyl-2,2,3,3-tetrafluoro-4-(4-propylphenyl)cyclohexane-1,4-diol (**3a**)*

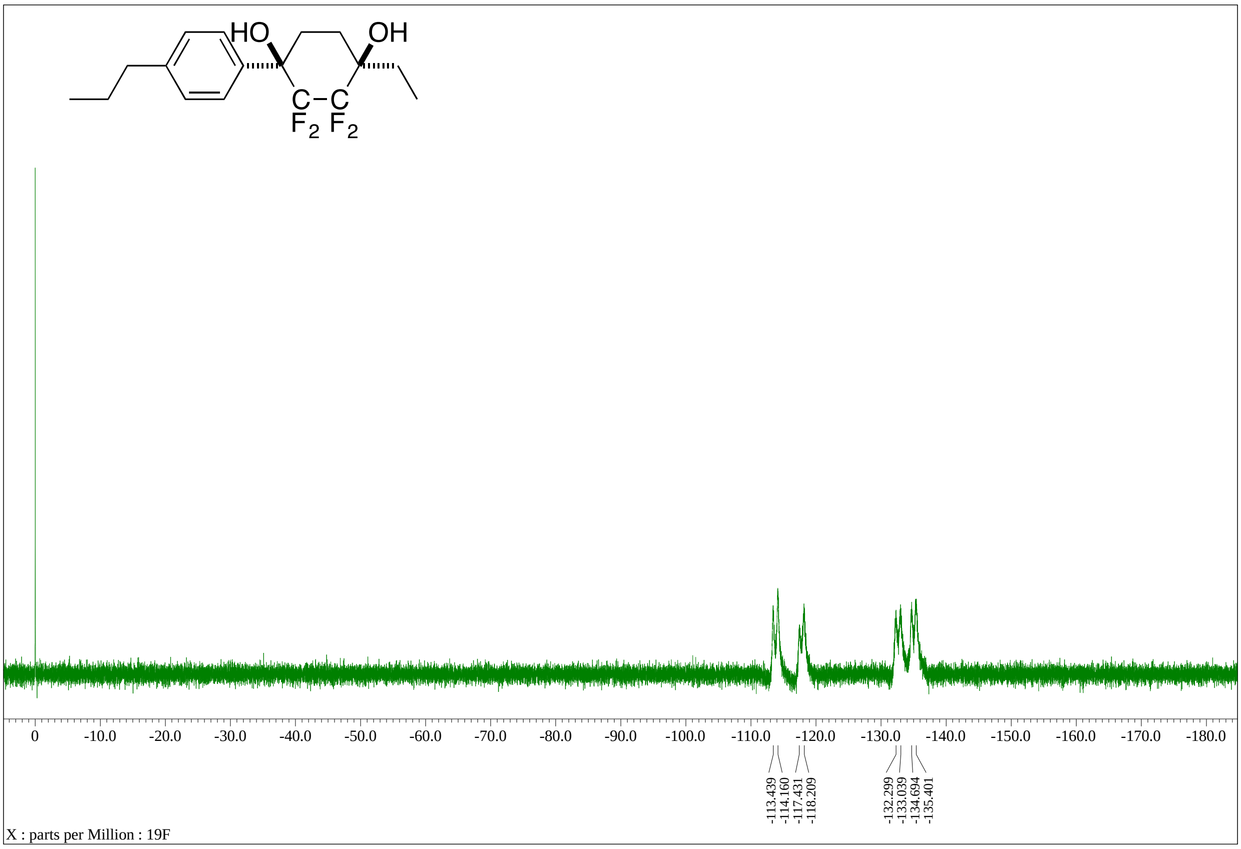

^{1}H NMR spectrum of 1-Ethyl-5,5,6,6-tetrafluoro-4-(4-propylphenyl)-1,3-cyclohexadiene (**1a**)

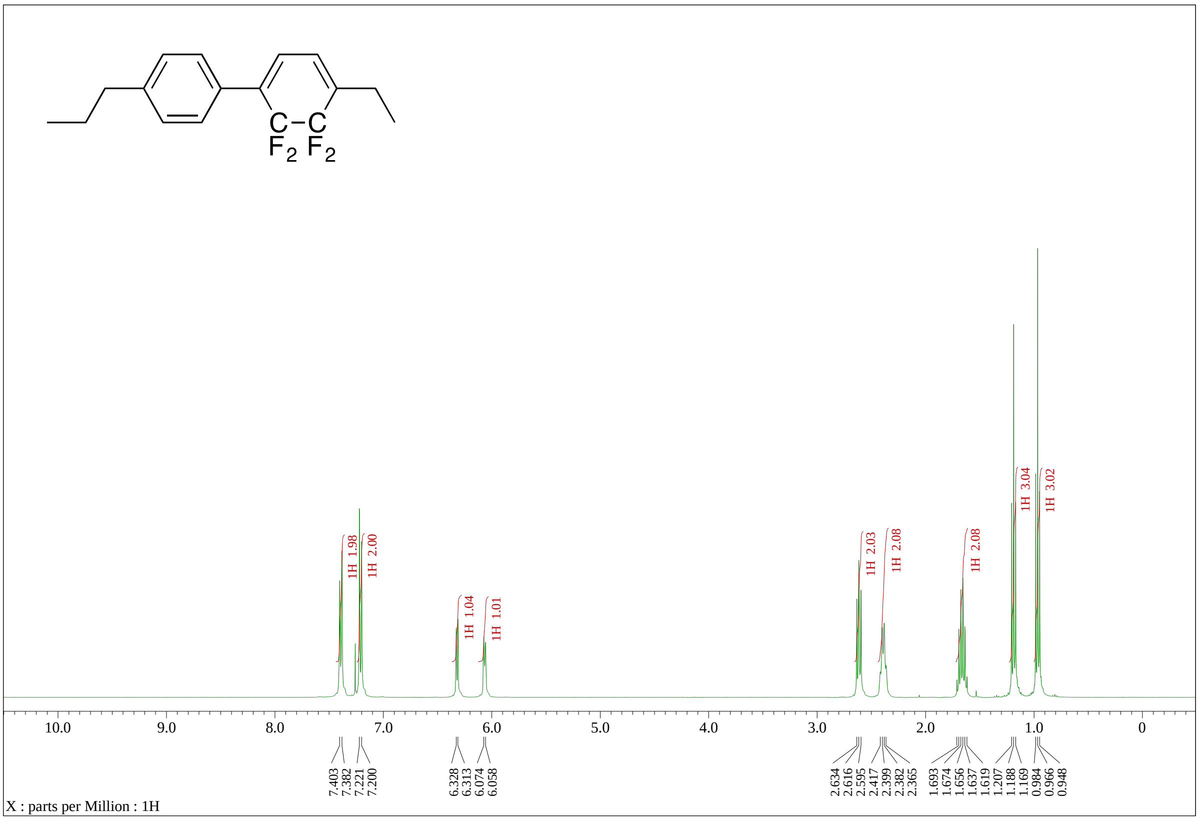

^{13}C NMR spectrum of 1-Ethyl-5,5,6,6-tetrafluoro-4-(4-propylphenyl)-1,3-cyclohexadiene (**1a**)

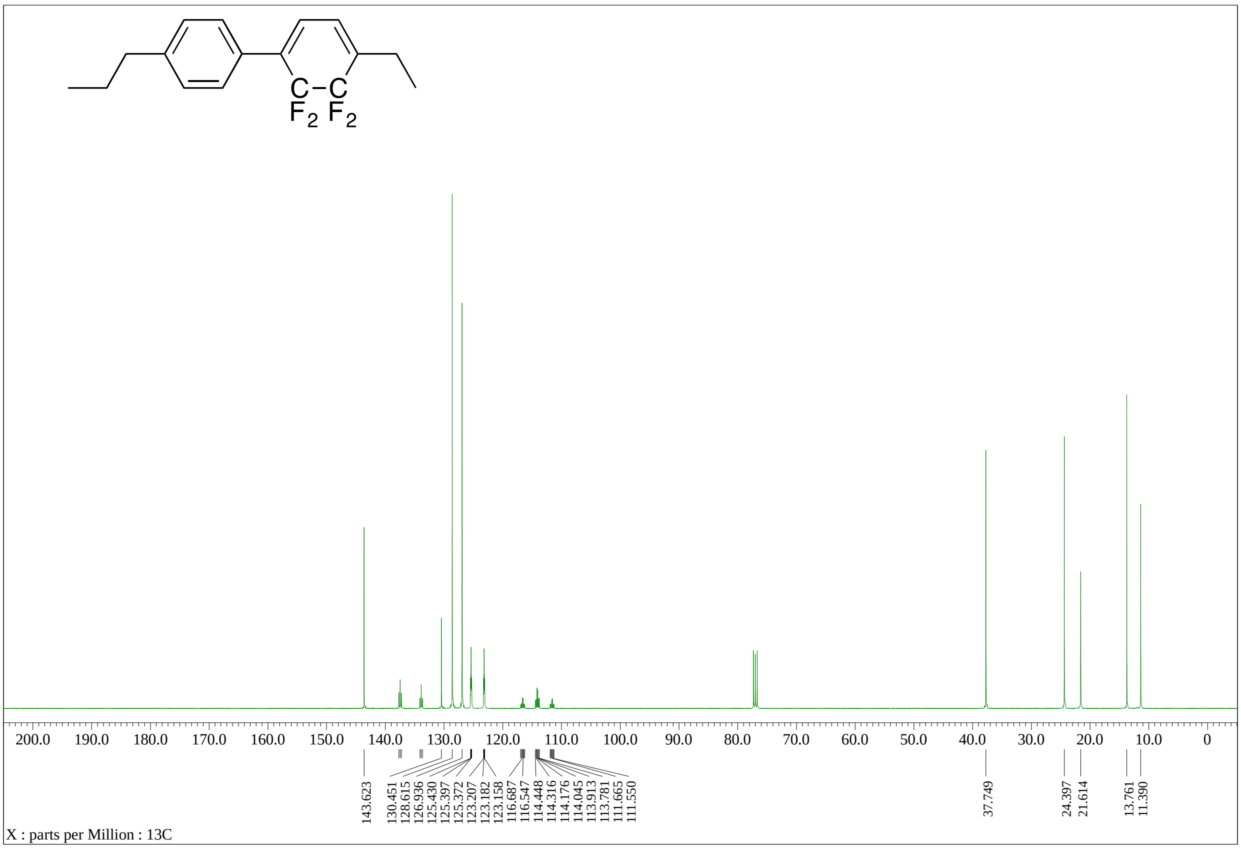

^{19}F *NMR spectrum of 1-Ethyl-5,5,6,6-tetrafluoro-4-(4-propylphenyl)-1,3-cyclohexadiene (**1a**)*

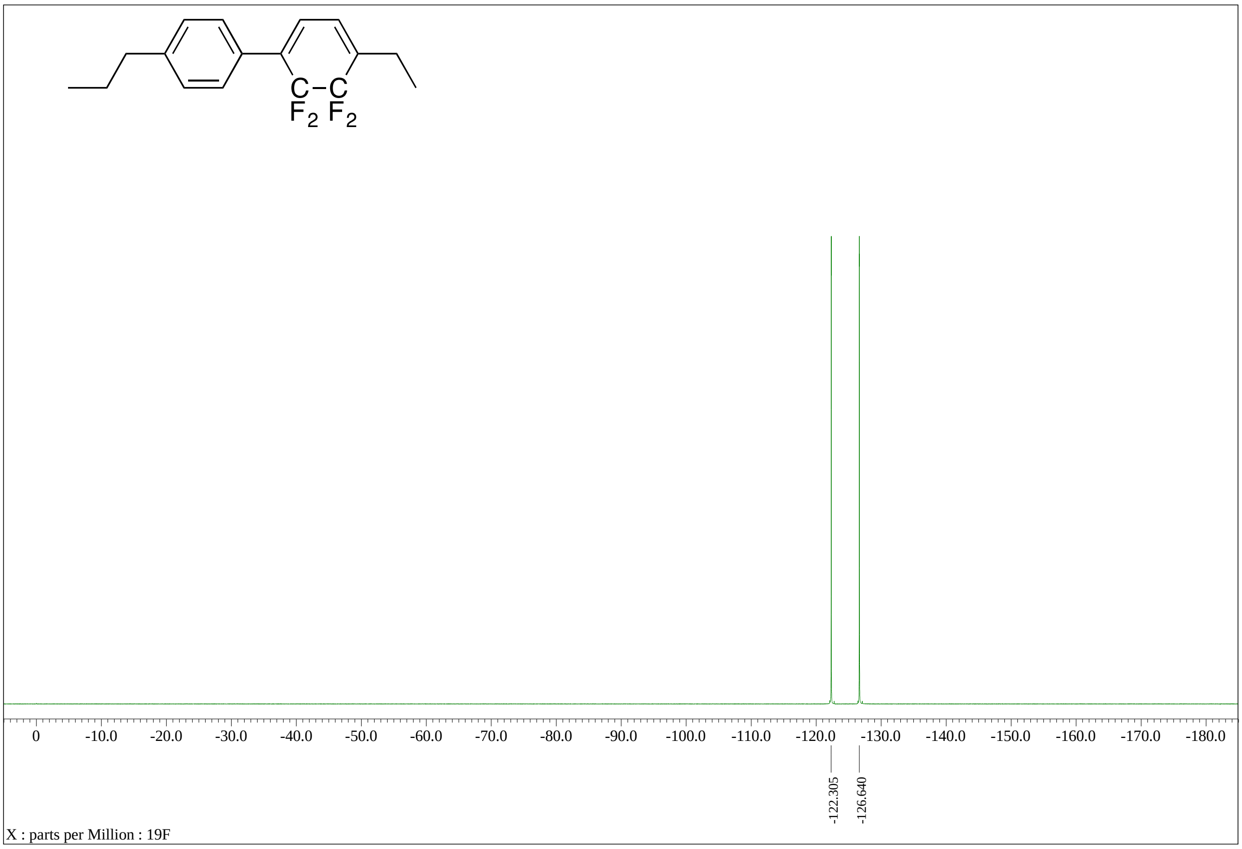

*^{1}H NMR spectrum of 1-ethyl-2,2,3,3-tetrafluoro-(4-propylphenyl)cyclohexane (**2a**)*

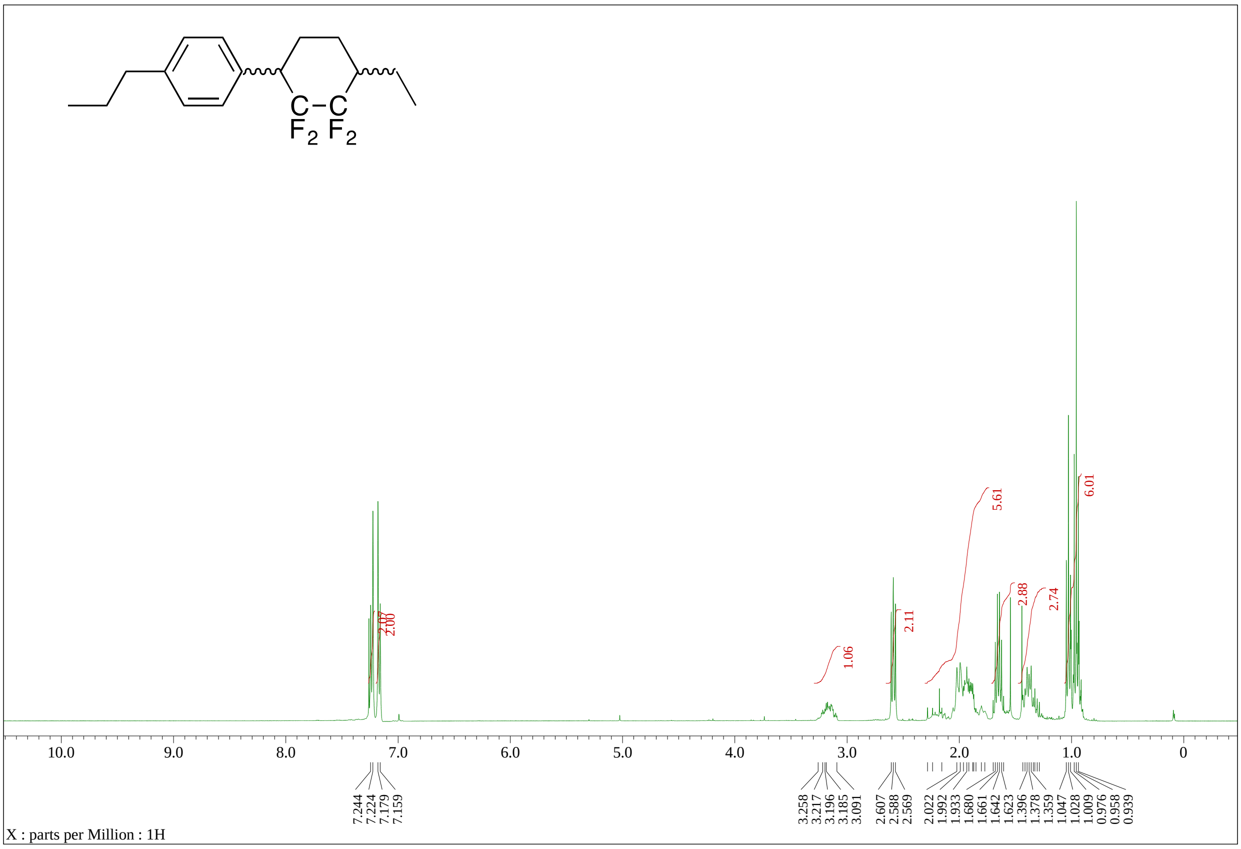

*^{13}C NMR spectrum of 1-Ethyl-2,2,3,3-tetrafluoro-(4-propylphenyl)cyclohexane (**2a**)*

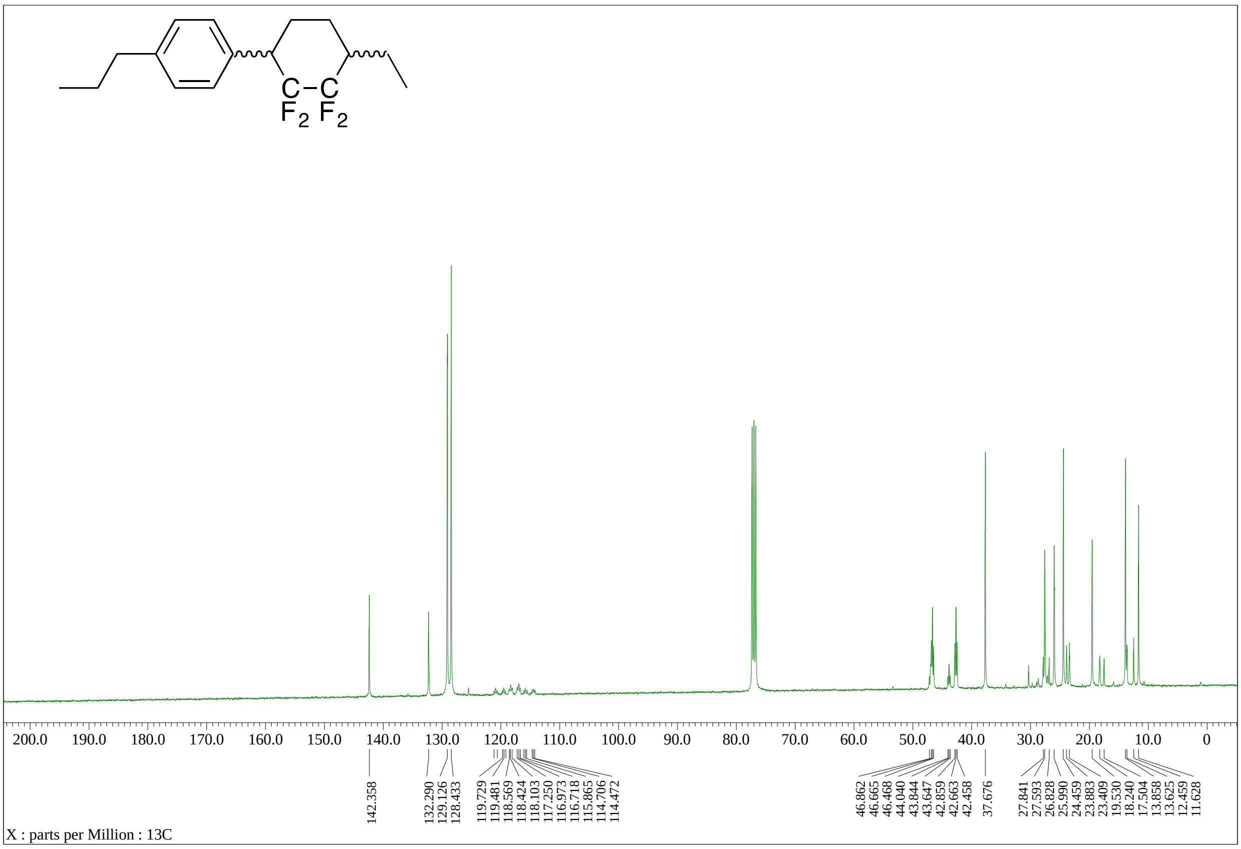

^{19}F NMR spectrum of 1-Ethyl-2,2,3,3-tetrafluoro-(4-propylphenyl)cyclohexane (**2a**)

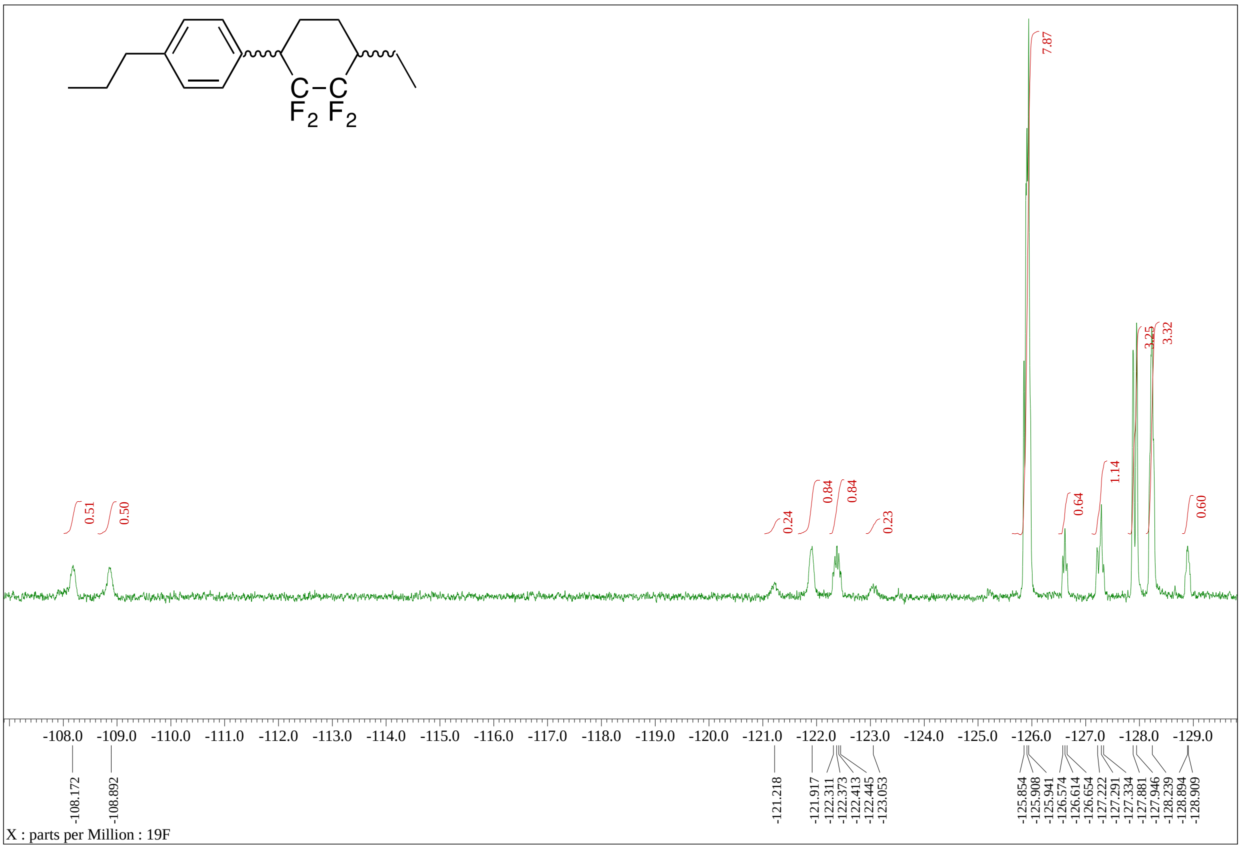

Supplement: File 1 — Experimental procedures, characterization data, and copies of 1H, 13C and 19F NMR spectra. [file Beilstein_J_Org_Chem-14-148-s001.pdf]
